# The R2R3-MYB Transcription Factor Gene Family in Maize

**DOI:** 10.1371/journal.pone.0037463

**Published:** 2012-06-07

**Authors:** Hai Du, Bo-Run Feng, Si-Si Yang, Yu-Bi Huang, Yi-Xiong Tang

**Affiliations:** 1 Maize Research Institute, Sichuan Agricultural University, Chengdu, Sichuan, China; 2 Key Laboratory of Biology and Genetic Improvement of Maize in Southwest Region, Maize Research Institute of Sichuan Agricultural University, Ministry of Agriculture, Chengdu, Sichuan, China; 3 Biotechnology Research Institute, Chinese Academy of Agricultural Sciences, Beijing, China; National Taiwan University, Taiwan

## Abstract

MYB proteins comprise a large family of plant transcription factors, members of which perform a variety of functions in plant biological processes. To date, no genome-wide characterization of this gene family has been conducted in maize (*Zea mays*). In the present study, we performed a comprehensive computational analysis, to yield a complete overview of the R2R3-MYB gene family in maize, including the phylogeny, expression patterns, and also its structural and functional characteristics. The MYB gene structure in maize and *Arabidopsis* were highly conserved, indicating that they were originally compact in size. Subgroup-specific conserved motifs outside the MYB domain may reflect functional conservation. The genome distribution strongly supports the hypothesis that segmental and tandem duplication contribute to the expansion of maize MYB genes. We also performed an updated and comprehensive classification of the R2R3-MYB gene families in maize and other plant species. The result revealed that the functions were conserved between maize MYB genes and their putative orthologs, demonstrating the origin and evolutionary diversification of plant MYB genes. Species-specific groups/subgroups may evolve or be lost during evolution, resulting in functional divergence. Expression profile study indicated that maize R2R3-MYB genes exhibit a variety of expression patterns, suggesting diverse functions. Furthermore, computational prediction potential targets of maize microRNAs (miRNAs) revealed that miR159, miR319, and miR160 may be implicated in regulating maize R2R3-MYB genes, suggesting roles of these miRNAs in post-transcriptional regulation and transcription networks. Our comparative analysis of R2R3-MYB genes in maize confirm and extend the sequence and functional characteristics of this gene family, and will facilitate future functional analysis of the MYB gene family in maize.

## Introduction

MYB transcription factor contains a conserved DNA-binding domain (DBD), which is homologous to the DBD of animal c-Myb [1]. This domain is generally composed of 1–4 imperfect repeats [2], [3]. Each repeat is approximately 50 amino acids in length and encoded by 3 α-helices. When bound to specific promoter sequences, the second and third helices form a helix-turn-helix (HTH) structure [2], [4]; the third α-helix is thought to play a recognition role in binding to a short DNA sequence [5]. Moreover, each repeat contains regularly spaced tryptophan residues, forming a tryptophan cluster in the 3-dimensional HTH structure [6]. The first plant MYB gene was isolated from maize, which encodes a c-MYB-like transcription factor involved in anthocyanin biosynthesis [7]. Subsequently, an increasing member of plant R2R3-MYB genes have been identified in a lot of plants. Substantial data now exist on the roles of MYB transcription factors in monocotyledonous and also in dicotyledonous plants [4], [8]–[12]. The most common type of plant MYB transcription factor is R2R3-MYB (containing 2 repeats).

Up to now, numerous plant R2R3-MYB genes (R2R3-MYBs) have been well annotated. The extensive expansion of this family in plants suggests that its members perform diverse functions in plant-specific processes. However, the only species for which the functions of the MYB gene family have been widely studied, based on the available genome sequence, is *Arabidopsis*. Recently, the increasing availability of plant genome sequences has facilitated a better understanding of this large gene family [13], [14]. Nevertheless, relatively few members of the maize R2R3-MYB gene family have been well functional characterized, comparing to *Arabidopsis*. Moreover, the R2R3-MYBs characterized in maize are limited in the control of phenylpropanoid metabolism pathway to date [7], [15]–[19]. In addition, the most recent comprehensive review of MYB genes in maize predates the completion of genome sequencing projects [13]. Consequently, the lack of genomic knowledge complicates the analysis of MYB gene family in maize, in particular the gene structures, intron pattern, phylogeny, and expression patterns. The completion of the maize genome sequence has enabled comparative genomics studies, and also the identification of new maize MYB genes (ZmMYBs) [20]–[22]. It is of interest for us that how many MYB genes exist in the maize genome? Given the large size of the MYB gene family, and its functionally diverse nature in *Arabidopsis*, genome data mining of this gene family in maize is crucial to understanding the roles of MYB transcription factors in maize physiological and biochemical processes. Furthermore, analysis of the structural relationships between *Arabidopsis* and maize MYB proteins would facilitate the prediction of the functions of, as yet, uncharacterized genes.

In the present study, we performed a genome-wide survey of the R2R3-MYB gene family in maize. A total of 158 open reading frames (ORFs) encoding R2R3-MYBs were identified, most of which remain to be functionally characterized. Subsequently, the deduced amino acid sequences encoded by the ORFs were subjected to an overview analysis, to yield clues concerning the evolutionary history of maize R2R3-MYB gene family. We revealed that segmental and tandem duplication events have contributed to the expansion of the maize MYB gene family. Using phylogenetic analysis of the R2R3-MYB families in maize, *Arabidopsis*, and other plant species, we designated these MYB genes into 37 subgroups. This facilitated the identification of shared and specific subgroups, suggesting the possible gene retention, and also loss and expansion processes of the MYB genes. By analysis of the intron pattern and conserved motif, we provide additional evidence for the subgroup definition. In addition, we also focused our investigation on mRNA expression analysis of ZmMYBs in different maize organs, and also compared the expression patterns of closely grouping paralogs. Since ZmMYBs present a variety of expression patterns, therefore, the presence of this large family in maize may be important in the control of gene expression in corresponding organs. Our study will serve as a foundation for future research into the functional roles of maize MYB genes.

## Results

### 157 Genes Encoding R2R3-MYB Proteins were Identified in the Maize Genome

To identify MYB encoding genes in maize genome, a preliminary BLASTP search was performed using the DBD sequences of known maize MYB proteins as queries. In each case, a large number of deduced amino acid sequences (>200 candidates) containing MYB or MYB-like repeats are obtained. Only hits with E values of <1.0 were considered as members of this gene family. Subsequently, the redundant sequences of candidate MYBs were discarded from our data set, according to their chromosome locations. In addition, the remaining maize MYBs possessing incomplete ORFs were also excluded for further analysis. All maize MYB proteins were manually inspected to ensure that the putative gene models contained 2 or 3 MYB repeats, and that the gene models mapped to unique loci in their respective genomes.

Finally, we identified 157 typical non-redundant R2R3-MYB proteins, and also 1 AtCDC5-like R2R3-MYB protein in maize genome. In order to distinguish the remaining R2R3-MYBs, we provisionally named them ZmMYB1 to ZmMYB158, based on the order of the corresponding chromosome locations identified from the maize genome browser (Table S1).

### Conserved Residues in the MYB Domain

**Figure 1:**
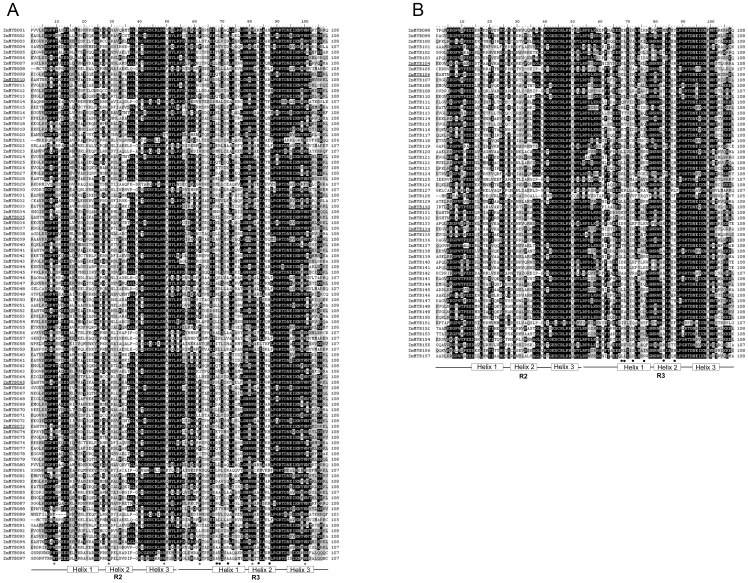
ClustalW amino acid sequence alignment of 157 typical maize R2R3-MYB domains. The shading of the alignment represents different degrees of conservation among sequences; the dark shading indicates identical residues, the light shading indicates conservative changes. The asterisks indicate conserved tryptophan residues (W) in the MYB domain. The positions of the three α-helices that form each MYB repeat are marked as Helix 1 to Helix 3. Residues involved in interaction are marked with dots; genes with these residues are underlined.

Similar to their counterparts in other plant species, the basic regions of maize R2R3-MYB domains contained, on average, ∼108 basic residues, with rare frequency of deletion or insertion (∼2%). By contrast, the region outside the DBD was the most divergent in terms of length, and also amino acid composition. Consistent with earlier reports, the R2 and R3 MYB repeats of ZmMYBs contained characteristic amino acids, including a series of evenly distributed and highly conserved Trp (W) residues (Fig. 1).

As shown in Figure 1, 6 Trp (W) residues at positions 9, 29, and 49 in the R2 repeat, and 62, 81, and 100 in the R3 repeat, form a hydrophobic core and serve as landmarks in the DBD of plant MYB proteins. In general, these Trp (W) residues, excepting Trp-62, are highly conserved in plant MYB DBDs. For example, substitution at Trp-9, Trp-49, and Trp-100 is found in only 1 ZmMYB, respectively. By contrast, most of the ZmMYBs had a substitution at Trp-62 (Fig. 1), and the exchanged amino acid was predominantly hydrophobic, such as Phe (F) or (less frequently) Ile (I), Leu (L), or Tyr (Y) (Fig. 1). In addition to these highly conserved Trp residues, a cysteine (C) located in the DNA recognition helix of R2 (Cys-45 in Fig. 1) was also completely conserved in the ZmMYB proteins.

In order to clarify the relationships between MYB DBD regions from different plants, we performed multiple alignment analysis of the MYB proteins in maize and *Arabidopsis*. Figure S1 shows the distribution of the 108 conserved amino acid residues in the R2R3-MYB domain. It was revealed that the linker region of R2 and R3 repeats was highly conserved, and that 4 amino acids in the first half of the linker (LNPE [L138 to E141 in chicken c-Myb R2R3]) formed a highly conserved motif [23], [24]. Interestingly, we observed a similar motif in the linker region in maize and *Arabidopsis* MYB proteins, as well (LRPD, Leu-53 to Asn-56 in Fig. 1). In addition, the evolution of plant-specific R2R3-MYB domain involved the insertion of a Leu residue (L) between the second and third helices of R2 repeat [25]. In the present study, we observed that up to 133 of the 157 typical ZmMYBs (about 85%) also had a Leu (L) insertion at the same site. However, the remaining 24 ZmMYBs (about 15%) have an insertion of a glycine (G), instead.

### Phylogenetic Analysis of the Maize R2R3-MYB Gene Family

**Figure 2:**
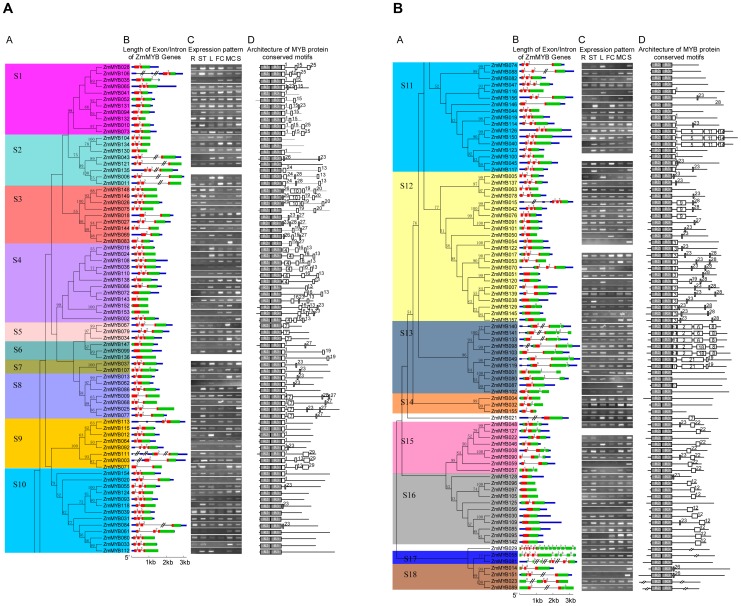
Phylogenetic relationships, intron pattern, expression pattern, architecture of conserved protein motifs, and subgroup designations in typical R2R3-MYB proteins from maize (Zm). A, The neighbor-joining (NJ) tree on the left includes 157 typical R2R3-MYB proteins from maize. The tree shows the 18 phylogenetic subgroups (S1–S18) marked with colored backgrounds, to facilitate subfamily identification with high predictive value. The numbers beside the branches represent bootstrap values (50%) based on 1000 replications. Eight proteins did not fit well into clusters. The colorful marker in the tree indicates the corresponding intron distribution patterns, as shown in Figure 3, below. B, The gene structure is presented by green exon (s), red MYB domain (s), blue UTR (s), and spaces between the colourful boxes corresponding to introns. The sizes of exons and introns can be estimated using the horizontal lines; the number indicated the phases of corresponding introns. C, The expression patterns of MYB genes in maize. The letter R above the column of expression data refers to root, ST refers to stem, L refers to leaf, FC refers to female catkins, MC refers to male catkins, and S refers to seed. D, Architecture of conserved protein motifs in 18 subfamilies. The motifs on the right were detected using MEME and are graphically represented as white boxes drawn to scale for a representative plant MYB protein of each subfamily.

Based on sequence similarity and topology, we subdivided the 157 typical members of the maize MYB gene family into 18 subgroups (designated S1–S18), according to clades with at least 50% bootstrap support (Fig. 2). Our results also showed that the topologies and bootstraps derived using the NJ method of the DBDs and the whole protein sequences were almost identical (Fig. 2 and Fig. S2). Moreover, the tree topology of maximum likelihood (ML) analysis was essentially the same with NJ trees as well (Fig. S3), indicating that these 2 methods were in good agreement.

In these 3 trees, only 2 ZmMYBs (ZmMYB021 and ZmMYB029) did not fit into any subgroup, indicating ambiguous clustering between the NJ and ML phylogenetic trees (Fig. 2; Figs. S2 and S3). The low bootstrap support for the internal nodes of these trees was in accordance with phylogenetic analysis of MYBs in other organisms. It was likely due to the fact that the MYB domain is relatively short, and that members within a subgroup are highly conserved, with relatively few informative character positions.

### Conserved Gene Structure Support to Subgroup Designation

**Figure 3:**
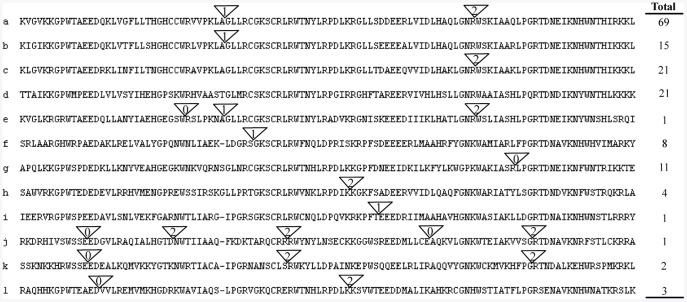
Schematic of the intron distribution patterns within the maize R2R3-MYB DNA-binding domains. Alignment of DNA-binding domains is representative of 12 intron patterns, named from a to l. Locations of introns are indicated by white triangles. The number within each triangle indicates the splicing phases of the MYB domain sequences: 0 refers to phase 0; 1 refers to phase 1; and 2 refers to phase 2. The number of ZmMYB proteins with each pattern is given on the right. The correlation of intron distribution patterns and phylogenetic subfamilies is provided in Figure 2.

In general, R2R3-MYBs possessed at least 1 intron in the DBD and up to 87% (136) of the 157 typical ZmMYBs possessed 1–5 intron(s) in the DBD; these introns could be grouped into 12 patterns, based on their presence and positions (Fig. 3). In contrast, outside the DBD, all but 20 of the 157 typical ZmMYBs lacked introns.

The most common pattern was a typical splicing of 3 exons and 2 introns (pattern a, 44% of ZmMYBs). Genes containing either of the 2 introns in pattern a constituted another 2 major groups of intron patterns, including subgroups S15 and S14 (Fig. 3, patterns b and c, respectively, accounting for ∼23% of ZmMYBs). In addition, ∼13% of ZmMYBs contain no intron in their DBD, forming the fourth main intron pattern (Fig. 3, pattern d; Fig. 2, S16). The remaining patterns exhibited varying intron positions (patterns e to l, accounting for ∼20% of ZmMYBs). Surprisingly, the phases in the same sites or patterns were also almost conserved. In the major splicing patterns a, b, and c, the introns phases were 1 and/or 2, respectively; where the phase at the same position of the R2 repeat was 1, and that at the R3 was 2 (Fig. 3).

In total, 29 conserved motifs with variable length (6–117 amino acids) were detected in the C-terminals of the maize MYB proteins (Table S2). We revealed that most members of a same subgroup shared 1 or more identical motifs outside the MYB DBD. Among which, some subgroup-specific motifs were previously characterized as additional functional properties, such as contributing to the regulatory specificity [4], [18]. The positions of the MYB domains and also any conserved motifs are shown in Figure 2, D. In contrast to previous studies [13], we observed that not only motif 1 (which was specifically conserved and directly followed the R3 repeat in some subgroups) but also some other motifs (including motifs 3, 17, and 23) selectively and directly followed the R3 repeat in some subgroups (Fig. 2, D).

### The Expression Pattern of Maize R2R3-MYB Genes

The expression pattern of a gene is often correlated with its function. Therefore, we analyzed the expression profiles of maize MYB gene family as well. The results showed that most of the 157 typical ZmMYBs yielded positive RT-PCR results. However, few ZmMYBs showed no expression signals (Fig. 2, C) which may be pseudogenes, or may be expressed at specific developmental stages or under special conditions. The wide expressions of ZmMYBs in roots, stems, leaves, flowers, and seeds suggested that they may involve in the development of all maize organs.

As shown in Figure 2, 59 out of the 157 typical ZmMYBs were expressed in all 6 tissues tested, suggesting that they may play regulatory roles at multiple developmental stages. However, most of the ZmMYBs showed preferential expression within different maize organs. For instance, 16 ZmMYBs showed preferential expression in the root, 40 ZmMYBs showed preferential expression in the stem, 34 ZmMYBs showed preferential expression in the leaf, and 41 ZmMYBs showed preferential expression in the seed. In addition, 29 ZmMYBs showed the highest transcript accumulation in male catkins, 38 ZmMYBs showed the highest accumulation in female catkins, and 30 ZmMYBs showed approximately equal transcript accumulation in male and female catkins. Interestingly, ∼136 out of the 157 typical ZmMYBs showed similar expression patterns in female catkins and the seed, supporting the hypothesis that they function in maize reproductive development. In general, the expression patterns of closely grouping paralogs are similar which suggests the function similarity. The similar expression patterns of closely grouping paralogs may suggest the function redundancy, while the paralogs with different expression patterns may share similar functions at different stage in plant development.

### Chromosomal Distribution and Duplication Events of Maize R2R3-MYB Genes

To date, the information regarding expansion events of the R2R3-MYB gene family in maize remain unclear. In order to investigate the relationship between genetic divergence and gene duplication within the MYB gene family in maize, we determined the chromosomal location of ZmMYBs, based on the information from the maize genomic database.

**Figure 4:**
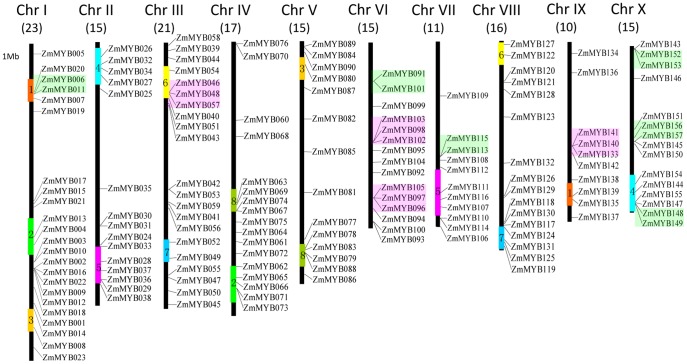
Chromosomal locations, region duplication, and predicted cluster for maize R2R3-MYB genes. The chromosomal position of each ZmMYB was mapped according to the maize genome. The chromosome number is indicated at the top of each chromosome. The number below indicates the number of ZmMYBs in each chromosome. The scale is in mega bases (Mb). The colored boxes indicate groups of predicted gene cluster with paralogous and syntenic genes on the chromosomes. The colored bars with numbers on the chromosomes indicate the 8 predicted duplication regions.

The result showed that the ZmMYBs were distributed throughout all the 10 maize chromosomes (Fig. 4). However, the distribution appeared to be uneven. In general, the central sections of chromosomes lack MYB genes. Relatively high densities of ZmMYBs were observed in the bottom of all chromosomes, excepting chromosome 5. In contrast, low densities were detected in the top of most chromosomes, excepting chromosome 3. Especially in the top half of chromosomes 4, 6, 7 and 9, large chromosomal regions lacked ZmMYBs. Subsequently, we analyzed the gene cluster expansion events of ZmMYBs in maize genome, based on these results.

Based on phylogenetic relationship and sequence similarity, we identified 35 gene clusters with high levels of protein sequence similarity (>90% within the DBD and >60% throughout the protein) (Fig. 4). For instance, the entire protein sequences of ZmMYB028 and ZmMYB106 shared 71% similarity, while those of ZmMYB010 and ZmMYB073 shared 78% similarity. Nevertheless, not all of the sister pairs were genetically linked to each other with respect to their corresponding chromosomal locations; ∼19 pairs (24%) of ZmMYBs lying within recently duplicated segmental chromosomes had a clear relative in these regions which may have evolved from putative maize genome duplication events. These multiple pairs linked each of at least 8 potential segmental duplications (Fig. 4, colored bars with numbers).

In addition, a series of tandem duplications were observed in maize genome as well. In total, we detected 24 (15%) very closely related ZmMYBs (ZmMYB006/ZmMYB011; ZmMYB101/ZmMYB091; ZmMYB113/ZmMYB115; ZmMYB148/ZmMYB149; ZmMYB152/ZmMYB153; ZmMYB156/ZmMYB157; ZmMYB046/ZmMYB048/ZmMYB057; ZmMYB098/ZmMYB102/ZmMYB103; ZmMYB096/ZmMYB097/ZmMYB105; and ZmMYB133/ZmMYB140/ZmMYB141) in a single cluster, respectively. These ZmMYBs were physically located near to each other in a syntenic region of related chromosomes, forming 10 ZmMYB sister pairs (Fig. 4, colored boxes).

### Comparative Analysis of Plant R2R3-MYB Proteins

**Figure 5:**
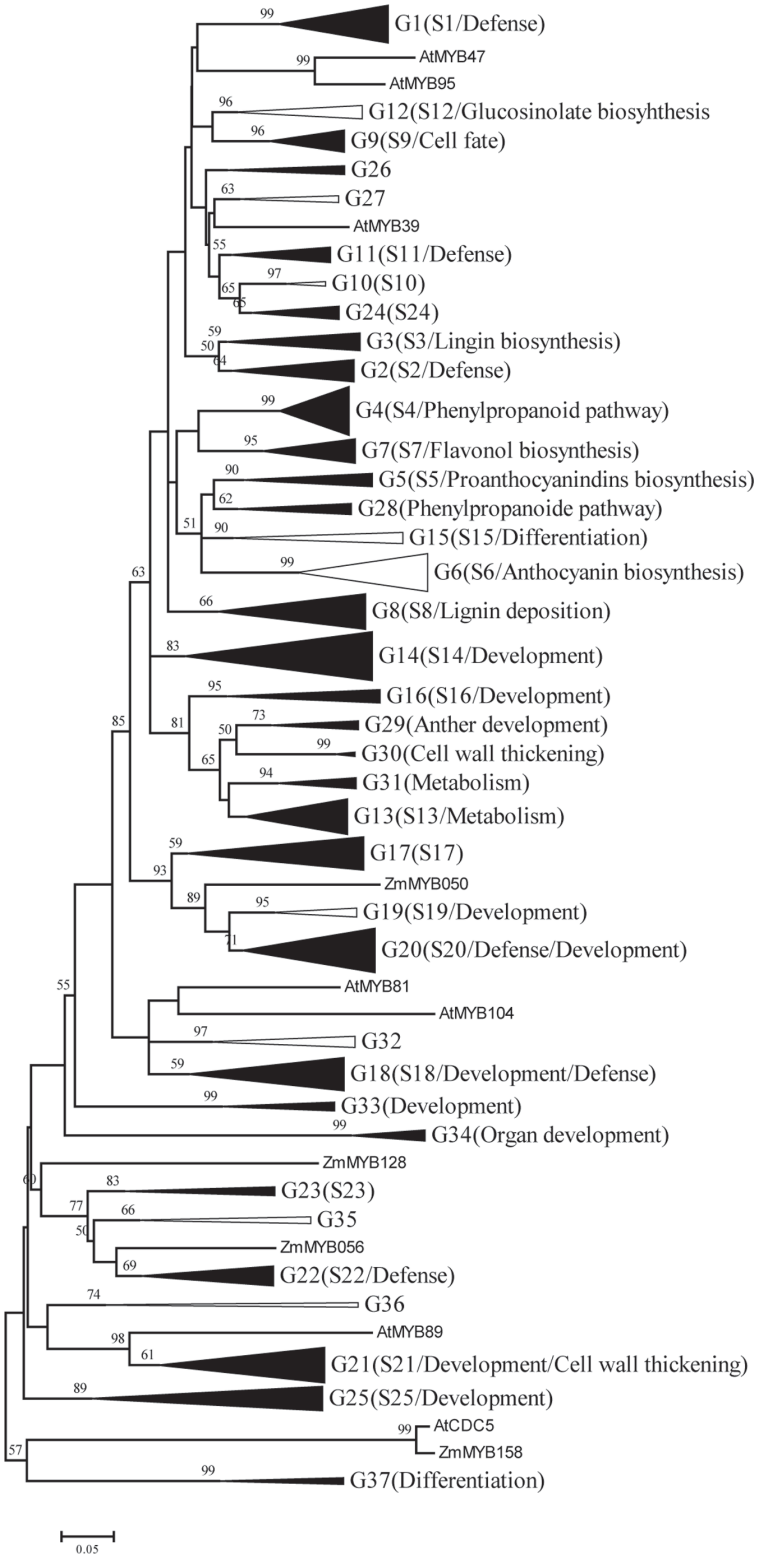
Phylogenetic tree of the R2R3-MYB proteins from maize (Zm), *Arabidopsis* (At), and other plant species. The neighbor-joining tree includes 158 R2R3-MYB proteins from maize, 126 R2R3-MYB proteins from *Arabidopsis*, and a further 52 R2R3-MYB proteins from other plant species. The proteins are clustered into 37 subgroups (triangles), designated with a subgroup number (e.g., G1). Subfamilies are represented as collapsed triangles, with depth and width proportional to sequence divergence and size, respectively. The black triangles indicate that the subgroup includes ZmMYBs and AtMYBs; the hatched and white triangles indicate that the subgroup includes or excludes ZmMYBs, respectively. Bootstrap values <50% are not shown in the phylogenetic tree. Four proteins did not fit well into clusters. The first 25 subgroups were designated as previously reports of AtMYBs by Stracke et al. (2001) and Dubos et al. (2010). The subgroups were listed in round bracket with annotated functions, for reference. 12 new subgroups were added because of the increased data set.

To update the functional clades with the predicted ZmMYBs, we performed a phylogeny reconstruction of the complete *Arabidopsis* R2R3-MYB superfamily (126 members) and 52 well-characterized R2R3-MYBs from other plant species, using the NJ method in Mega4 (Fig. 5 and Fig. S4), and the maximum likelihood (ML) method in PhyML (Fig. S5), respectively. With the exception of a few nodes with low support, the phylogenetic trees derived from each method had very similar topologies. Based on our results and also referring to previous studies of the *Arabidopsis* MYB gene family [3], [4], we subdivided these MYB genes into 37 subgroups (Fig. 5).

As shown in Figure S4, 29 out of 37 subgroups were present both in maize and *Arabidopsis*. Thus, it is likely that the appearance of most genes in these species predates the divergence of monocot/eudicots. This may represent the ancient origin of MYB genes that have played a conserved and crucial role during plant evolution. Meanwhile, some species-specific subgroups were also observed, indicating that MYB genes may have evolved or been lost in a single species, following divergence. For example, members of subgroup G12 were recently identified as being involved in glucosinolate biosynthesis [26]–[29] (Fig. 5). However, no ZmMYBs were clustered within this subgroup, possibly because the glucosinolate compounds were *Cruciferae* plant-specific [30]. In another example, subgroup G15 consisted of AtMYB0/GL1, AtMYB66/WER, and GhMYB109, all of which are involved in epidermal cell development [31]–[34]. Nevertheless, in maize, there appears to be no orthologs in this subgroup, suggesting the possible gene loss and/or lineage-specific expansions, which may reflect species-specific adaptations. While subgroup G6 is more likely to be eudicot-specific clade, for which contains MYBs from as many as 13 eudicot species, forming a separate branch with higher support value.

In addition, 4 of the 37 subgroups contained only MYB members from maize, suggesting a gene acquisition mechanism from the most recent common ancestor with *Arabidopsis*, during the evolution. Our expression analysis revealed that maize R2R3-MYBs have a variety of expression patterns in different tissues. Therefore, we believe that these species-specific subgroups may regulate essential biological processes during maize development.

## Discussion

### Characteristics of Maize MYB DNA-binding Domain

In the present study, we identified a putative full set of R2R3-MYBs in the maize genome, comprising a total of 157 typical R2R3-MYB encoding genes. In addition to the highly conserved Trp amino acid residues (W) in the DBD domain, we observed alternative highly conserved residues, and also some important amino acid substitutions (Fig. S1). The highly conserved residues were generally distributed at both ends of each repeat, especially in the third helix. Our results indicate that during the evolution, the third helix is more conserved than the first 2 helixes. It was previously demonstrated that the residues in the third helix are important for DNA binding activity, by interaction with the DNA bases in the major groove, when bound to DNA [34]. Therefore, the highly conserved characteristic of the third helix may indicate functional conservatism among different plant species during MYB evolution, while species-specific genes may be derived from key residue exchanges in this region. Accordingly, the substitutions in the third helix may result in recognition of novel target genes and/or may significantly impair the DNA-binding activity. This, in turn, dictates the transcriptional regulatory role in most biological processes.

In general, the distributions of conserved amino acids among the MYB DBDs of both maize and *Arabidopsis* were very similar, indicating that the amino acid residues in this domain are highly conserved across plant MYB genes. Nevertheless, 15 out of the 108 (about 14%) positions differed between these 2 species (Fig. S1). However, the difference was not significant, and it was generally a transpose of the residues at the same site, based on the percentage (Fig. S1). For instance, more ZmMYBs have a Val-31 (A) residue and Ile-31 (S) residue in the R2 repeat of the MYB DBD. By contrast, in *Arabidopsis*, the first two common residues at the same position were Ile-31 (S) and Val-31 (A), respectively. Our findings indicate that such sites in the DBD domain may have limited variability among certain residues.

Moreover, it has been demonstrated that some substitutions in the MYB DBD have a dramatic effect on the DNA-binding activity of MYB proteins [5], [23], [24]. For example, Cys-41 in the R2 repeat was highly conserved in typical R2R3-MYB DBD during evolution (Fig. 1), which was demonstrated to be essential for the DNA-binding activity of a maize R2R3-MYB gene, P1 [35]. Surprisingly, up to 33% (52 out of 157) of the ZmMYBs had a substitution at Cys-41, which may also effect the functions of corresponding ZmMYBs, as well. In addition, it was further reported that substitution of residues within the linker region led to reduced stability of protein–DNA complexes, and even loss of DNA-binding ability [35], [36]. Surprisingly, in our study, 5 ZmMYBs have a substitution at Pro-55 (P) by Ala (A) in the linker region, and are consequently predicted to affect the binding ability.

### Structure, Protein Domain Relationships, and Evolution of Maize MYB Family Genes

Phylogenetic analysis of the ZmMYB gene family showed that the genes in the same subgroups or subclades generally contained the same intron pattern (Fig. 2, B), with the position(s) of the intron(s) being fully conserved. Moreover, the number of introns in the MYB DBDs appeared to be limited; among the 157 typical ZmMYBs, a majority of ZmMYBs (97%) had no more than 2 introns. These results validate our classification of ZmMYBs, and indicate that the intron patterns and their corresponding splicing phases are not random, but highly conserved. Introns would be inserted or excised from the MYB coding region in a subfamily-specific manner, suggesting that the introns have been specifically inserted into plants and retained in the genome, during the evolution. In addition, we observed an excess of phase 1 and 2 introns, and of assymmetric exons within the MYB DBD, which may facilitate alternative splicing (AS) [37], [38], such as exon shuffling and intron retain events [39], [40]. Corresponding, we detected AS event (2–6 events) in 23 of the 157 typical ZmMYBs, resulting in a variety of transcripts from a single gene (Table S1).

Most plant MYB proteins are composed of a set of conserved motifs in the C-terminal and the protein architectures are remarkably conserved within specific subgroups. Members of the same subgroups generally shared 1 or more identical motifs outside the MYB DBD. The schemes of protein motifs of individual members of the MYB gene family indicated structural similarities within subgroups, further supporting the subgroup definition in phylogenetic analysis. This also may indicate that the highly conserved protein motifs are protein domain combinations, often lineage-specific. Although most of these conserved motifs remain to be functional elucidated, it is likely that some play important roles in the transcriptional regulation of target genes and may promote further functional diversification in specific lineages.

In general, multiple members of a specific gene family are believed to result from the long evolutionary history of a particular organism. The individual members of a gene family reflect a succession of genomic rearrangements and expansions, caused by extensive duplication and diversification during the course of evolution [13]. In the present study, we observed that large-scale segmental duplication and tandem duplication events were logically the contributors to the expansion of the MYB gene family in maize, following their divergence. Interestingly, the DBDs between ZmMYB148/ZmMYB149, ZmMYB015/ZmMYB042, and ZmMYB140/ZmMYB141 were completely identical. Excepting for a few differences in the C-terminal, their corresponding whole sequences were also very similar (>90%). Thus, it is inferred that new gene initially resulted from the duplication, and thereafter from a series of synonymous and/or non-synonymous mutations in the whole sequence (especially in the MYB DBD), to perform new functions. In addition, the presence of 3 tandem arrays of ZmMYB006/ZmMYB011; ZmMYB046/ZmMYB048/ZmMYB057; and ZmMYB148/ZmMYB149 (located on the recently duplicated segmental chromosome in chromosomes 1, 3, and 10, respectively) suggests tandem duplication of the ancestor of these genes posterior to the most recent segmental duplication (Fig. 4).

### Phylogenic Relationship and Functions of Maize MYB Family Genes

R2R3-MYB transcription factors play important roles in the regulation of secondary metabolism, the control of cell shape, the response to various stress conditions, and hormone responses in higher plants. In spite of their large number and significance, very few of these genes have been functionally characterized in monocots, such as maize. In general, the orthologs clustered in a subgroup or subclade (functional clade) shared similar gene architecture structure and functions, indicating recent common evolutionary origins. In other words, knowledge of the functions of certain members should facilitate the confirmation of paralogous and orthologous functional relationships. Phylogenetic comparative analysis of R2R3-MYBs in different plant species revealed considerable diversification and conservation of the MYB gene family in plant. The major groups/subgroups contained members of orthologous genes belonging to maize, *Arabidopsis*, and/or other plant species, suggesting that the appearance of many of these genes predates monocot/eudicot divergence. However, species-specific groups/subgroups also existed which were evolved or lost during expansion of the MYB gene family, resulting in functional divergence. Though the roles of most ZmMYBs remain to be elucidated, it is likely that members of a given group/subgroup may have recent common evolutionary origins and also a conserved function.

According to our analysis, most ZmMYBs are clustered with orthologs of plant R2R3-MYBs in different subgroups (Fig. 5 and Fig. S4), suggesting the functional conservation of plant MYBs. Many outstanding examples of functional conservation are demonstrated in our analysis. For example, subgroup G7 consists of 10 plant MYBs, including 4 ZmMYBs *p1*, *p2*, *MYB-IF25*, and *MYB-IF35*, implicated in the control of flavonol biosynthesis [16]–[18], [41]–[46]. Subgroup G5 clusters 6 MYBs from different species involved in the control anthocyanin biosynthesis [7], [47]–[51]. In accordance with the expression profile analysis, the two maize counterparts (*ZmMYB134/C1* and *ZmMYB104/PL*) in this subgroup showed preferential expression within the flower (Fig. 2, C). While in subgroup G4, members of *AtMYB4*, *AtMYB32*, *EgMYB1*, *TaMYB4*, *PhMYB4*, *ZmMYB028/Zm31*, and *ZmMYB073/Zm42* are associated with negatively regulation of phenylpropanoid biosynthesis; for example, AtMYB4 acts as a negative regulator of sinapate ester biosynthesis through regulating the expression of the gene encoding cinnamate 4-hydroxylase (*C4H*), whereas ZmMYB31 and ZmMYB42 repress lignin biosynthesis by down-regulating caffeic acid *O*-methyl-transferase (*COMT*) gene expression [19], [52]–[57]. Moreover, all of these genes possess the C2 motifs, known to participate in repression of phenylpropanoid biosynthesis, further supporting the hypothesis of structure and function similarity of MYB genes.

Plant MYB transcription factors are also well known to play a key role in the control of plant development. Subgroup G34 consists of 5 RS2/AS1/PHAN orthologs (including *ZmMYB021/RS2*) involved in organ development by repressing expression of the KNOX genes that are required for normal initiation and development of lateral organs (Fig. 5) [58]–[62]. In subgroup G14, 13 ZmMYBs clustered together with a group of Blind-like MYB genes that are involved in plant development, such as axillary meristem regulation, and lateral organ formation, etc [63]–[66]. Although no Blind-related genes have been reported in maize to date, the ZmMYBs clustered in this subgroup share high sequence similarity with these Blind-related genes, suggesting a similar functional feature of these ZmMYBs in plant development. An interesting question for future research will be to investigate whether these maize MYB orthologs within this subgroup have retained the ancestral function or have evolved new functions.

**Figure 6:**
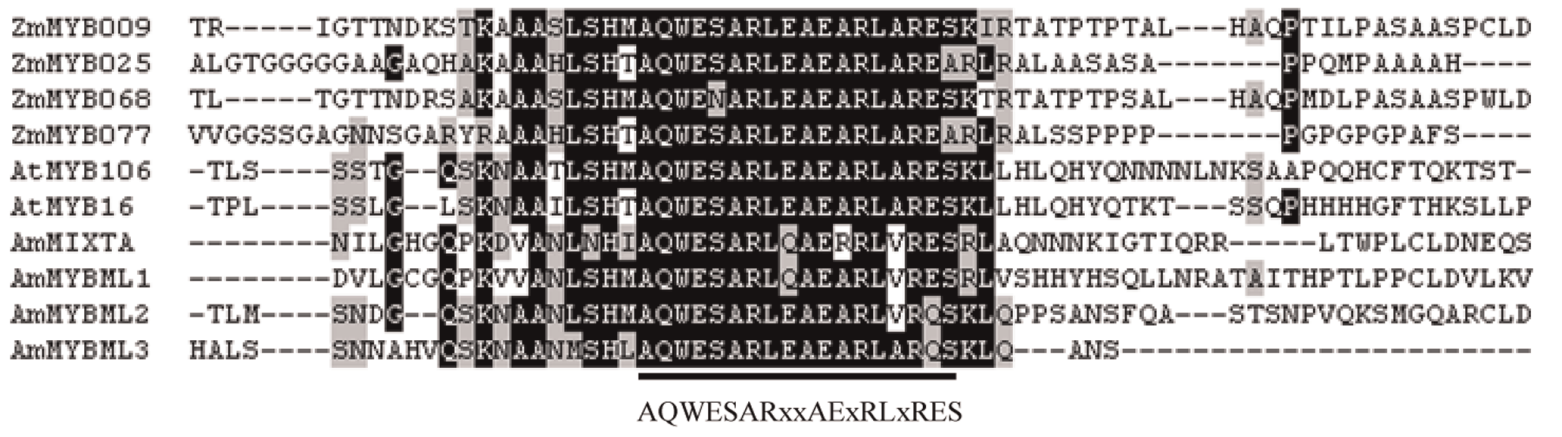
A putative motif conserved in group G9 proteins. Black and gray shading indicate the presence of identical and conserved amino acid residues, respectively, in >75% of the aligned sequences. Consensus amino acid residues are shown below the alignment. The ‘‘x’’ indicates no conservation at this position.

Another well-known role of plant MYB transcription factors is controlling the cell fate. For instance, subgroup G9 contains several plant MIXTA-like genes (Fig. 5). These closely related MIXTA-like genes are proved to be involved in conical-papillate petal cell development, petal trichome differentiation and petal morphogenesis, respectively [67]–[72]. In addition, excepting the highly conserved DBD, a conserved sequence motif (AQWESARxxAExRLxRES) exists downstream of the DBD of the MIXTA-like MYB proteins [70]. 4 ZmMYBs (*ZmMYB009*, *ZmMYB025*, *ZmMYB068*, and *ZmMYB077*), also grouping within this subgroup, have been shown to possess the same motif (Fig. 6). This finding strongly supports a similar role for these orthologs in cell fate in maize.

GAMYB or GAMYB-like genes encode a highly conserved subgroup of R2R3-MYB proteins that have been implicated in GA signal transduction [72]–[77]. In our phylogenetic tree, 6 ZmMYBs together with barley (*Hordeum vulgare*) *GAMYB*, rice *GAMYB*, tomato *GAMYB*, *Lolium temulentum GAMYB* and 7 *Arabidopsis* GAMYB-like genes were clustered in subgroup G18 (Fig. 5). The expressions of these genes were all previously demonstrated to be consistent with their roles in GA-mediated processes [78]–[82]. Moreover, it was further suggested that *GAMYB* gene contains a unique intron located at the 3′ end of its ORF. Interestingly, the 6 ZmMYBs included in this subgroup also have an intron at their C-terminal, implying that they belong to a distinct clade within the MYB transcription factor family (data not show). Furthermore, *GAMYB* was indicated to be negatively regulated by the microRNA (miRNA) family member, miR159 [75], [82]–[84]. Based on recently available genome-wide characterization of microRNA genes in maize [85], we revealed that 3 of these 6 maize GAMYB-like genes (*ZmMYB049*, *ZmMYB080*, and *ZmMYB119*) were predicted to be the target genes of maize miR159, based on similarities. Interestingly, the targets of miR159 in plant MYB gene family were limited to the GAMYB-like genes to date [76], [83]. However, our results indicate that, excepting miR159, miR319 may be also implicated in regulating 2 maize GAMYB-like genes (*ZmMYB049* and *ZmMYB119*) in this subgroup. In addition, miR828 and miR858 have been confirmed to target several R2R3-MYBs in *Arabidopsis* as well [86]. In our study, we did not found any ZmMYB as the target of miR828 or miR858, but we revealed that miR160 and miR159 may target 8 additional ZmMYBs (*ZmMYB004*, *ZmMYB054*, *ZmMYB101*, *ZmMYB103*, *ZmMYB119*, *ZmMYB122*, *ZmMYB140*, and *ZmMYB155*). Because of these genes were clustered in 4 different subgroups (Fig. S4), it is indicated that microRNA may be extensively involved in regulating the expressions of MYB genes. Our findings suggested the roles of these miRNAs in post-transcriptional regulation and transcription networks, and provide a valuable framework for further elucidation of miRNA functions in the MYB gene family.

In general, the C-terminal regions of MYB proteins often possess protein–protein interaction domain. To date, the interaction of R2R3-MYB protein, basic helix-loop-helix (bHLH) transcription factor and WD40 protein has been well studies [7], [87], [88]. The interaction of MYB protein and bHLH protein was firstly reported in two of maize MYB proteins C1 and Pl [7]. Subsequently, in *Arabidopsis*, up to 13 R2R3-MYB proteins (including AtMYB3, AtMYB4, AtMYB5, AtMYB7, AtMYB0/GL1, AtMYB23, AtMYB32, AtMYB66/WER, AtMYB75/PAP1, AtMYB90/PAP2, AtMYB113, AtMYB114 and AtMYB123/TT2) were also implicated in interaction with bHLH proteins and a WD40 repeat protein, TTG1[47], [89]–[93]. It was further reported that 6 highly conserved amino acid included in a motif ([DE]Lx2[RK]x3Lx6Lx3R; Fig. 1) in the first two helixes of R3 repeat were necessary for the interaction with bHLH protein [87], [94]. Interestingly, these 6 residues are also found in 8 ZmMYBs that were clustered the same subgroups (G4, G5 and G28, Fig. 1 and Fig. S4), strongly suggests the function similarity of them. These results showed that the combinatorial regulation mechanism may involve in many plant biological processes, including the anthocyanin biosynthesis [48], [53], proanthocyanidin and synapate ester biosynthesis [47], [49], [52], as well as trichome and root hair patterning [90] etc.

In total, 9 out of the 37 MYB subgroups lack any functionally characterized members. The existing knowledge of the functions of the R2R3-MYB gene family in plants is summarized in Table S3. This data will facilitate the characterization of each subgroup of the maize MYB gene family.

## Methods

### Identification of MYB Protein in Maize

To identify maize genes encoding MYB proteins with at least 1 possible DBD, we performed a BLASTP search (http://www.phytozome.net/search.php) at the Joint Genome Institute (JGI) (http://www.phytozome.net/cgi-bin/gbrowse/maize/), and/or the MaizeSequence (http://www.maizesequence.org/index.html) genome database, using the amino acid sequences of the known maize R2R3-MYB DBDs (about 108 amino acids) as queries. To verify the reliability of our results, the functional and structural domains were predicted by PROSITE profiling (http://www.expasy.org/tools/scanprosite/) [95] and SMART analysis (http://smart.embl-heidelberg.de/) [96], respectively.

In addition, the sequences of 126 *Arabidopsi*s R2R3-MYB proteins [4] were downloaded from the TAIR *Arabidopsis* genome (http://www.arabidopsis.org/). The predicted proteins of 52 well-known plant R2R3-MYB genes were collected from the National Center for Biotechnology Information (NCBI, http://www.ncbi.nlm.nih.gov/).

### Sequence Analysis

To analyze the sequence features of the 157 typical identified maize R2R3-MYB proteins, we performed multiple alignment analyses of the MYB DBDs by ClustalW (www.ebi.ac.uk/clustalw/) [97]. In order to obtain optimized alignment, the deduced amino acid sequences were adjusted manually using BioEdit (version 7.0.0) with the default parameters (Pittsburgh Supercomputing Center; http://www.psc.edu/biomed/genedoc/) [98].

The intron pattern represents an independent criterion to support subgroup designations of phylogenetic analysis. Therefore, we analyzed the intron pattern (including the distribution, position, and phases) of the maize R2R3-MYB encoding genes. Based on the results of BLASTP searches in the maize genome database, we obtained the information on cDNA sequences, genomic sequences, intron distribution pattern, and phases and intron/exon boundaries of the genes. We also obtained information on the chromosome locations of each gene from the results of BLASTP at MaizeSequence.

In order to identify potential protein motifs outside the maize R2R3-MYB DBDs, we used the MEME version 3.5.7 tool [99], with the following parameter settings: the distribution of motifs, 0 or 1 per sequence; maximum number of motifs to find, 100; minimum width of motif, 6; maximum width of motif, 300; and the motif must be present in all members within the same subgroup. In addition, only motifs with an e-value ≤1e–10 were kept for further analysis. Subsequently, the MAST program was used to search detected motifs in protein databases [100].

### Construction of the Phylogenetic Trees

In order to obtain clues about the evolutionary history of the R2R3-MYB gene family in maize, we constructed a neighbor-joining (NJ) tree, based on the multiple sequence alignment of all predicted maize MYB DBDs, using MEGA version 4.0. For statistical reliability, we conducted bootstrap analysis with 1000 replicates. To test the reliability of the result, we performed maximum likelihood (ML) analysis of the MYB DBDs, using the software PhyML (http://atgc.lirmm.fr/phyml/) [101], as well as a NJ analysis of whole ZmMYB protein sequences by MEGA4.0.

To compare the evolutionary relationship of different plant MYB genes, we further constructed a phylogenetic tree of the 158 ZmMYBs, 126 AtMYBs, and 52 well-known R2R3-MYB proteins, using the NJ method of MEGA, with the following parameters: Poisson correction; pairwise deletion; and bootstrap (1000 replicates). To validate the results from the NJ method, we also constructed a maximum likelihood (ML) tree using the software PhyML with bootstrap of 1000 replicates. Tree files were viewed using FigTree v1.3.1 (http://tree.bio.ed.ac.uk/software/figtree/).

### Expression Analysis of R2R3-MYB Gene Family in Maize

Using reverse transcription (RT)-PCR analysis, we analyzed the expression information of the 157 typical ZmMYBs. All gene-specific primers were designed to avoid the conserved region (based on the C-terminal regions of ZmMYB sequences), amplifying products of 100–400 bp long. The specific primer for the maize actin gene was used as an internal control (310 bp). The detailed PCR primer sequences are shown in Table S4.

RNA of roots, stems, leaves, and flowers of maize cultivar Chuandan 13 was isolated from plants with 8–10 cm inflorescences. The corresponding plant tissues of maize were harvested and ground in liquid nitrogen. Total RNA was extracted using Trizol reagent (Invitrogen, Germany), according to the manufacturer’s instructions, and treated with DNase I (Promega, USA). First-strand synthesis of cDNA was performed using an oligo (dT) primer and M-MuLV RT kit (Takara Biotechnology, Japan) (2 µg of total RNA was used for RT in a 20-µl reaction volume). Each PCR pattern was independently verified in at least 3 replicate experiments performed under identical conditions. PCR products were fractionated on 1% agarose gels containing ethidium bromide, and photographed under UV light. To confirm the validity of RT-PCR, approximately 10 samples were randomly selected for sequencing (data not shown).

## Supporting Information

Figure S1 Sequence comparison of the DNA-binding domains in maize and *Arabidopsis* R2R3-MYB genes.
(PDF)

Figure S2 Phylogenetic tree of 157 typical maize R2R3-MYB family genes.
(PDF)

Figure S3 ML phylogenetic tree of 157 typical maize R2R3-MYB family genes.
(PDF)

Figure S4 Phylogenetic tree of the R2R3-MYB proteins from maize (Zm), *Arabidopsis* (At) and other plants.
(PDF)

Figure S5 Maximum likelihood (ML) tree of the R2R3-MYB proteins from maize (Zm), *Arabidopsis* (At) and other plants.
(PDF)

Table S1 Summary of the R2R3-MYB transcription factor genes in maize.
(PDF)

Table S2 Motifs outside the MYB DNA-binding domain of maize R2R3-MYB proteins.
(PDF)

Table S3 Summary of functionally characterized R2R3-MYBs from plants examined in this study by MYB subgroups.
(PDF)

Table S4 Details of primers used in the RT-PCR analysis.
(PDF)

## References

[1] Klempnauer KH, Gonda TJ, Bishop JM (1982). Nucleotide sequence of the retroviral leukemia gene v–myb and its cellular progenitor c–myb: the architecture of a transduced oncogene.. Cell.

[2] Lipsick JS (1996). One billion years of Myb.. Oncogene.

[3] Dubos C, Stracke R, Grotewold E, Weisshaar B, Martin C (2010). MYB transcription factors in Arabidopsis.. Trends Plant Sci.

[4] Stracke R, Werber M, Weisshaar B (2001). The R2R3–MYB gene family in Arabidopsis thaliana.. Curr Opin Plant Bio l.

[5] Rabinowicz PD, Braun EL, Wolfe AD, Bowen B, Grotewold E (1999). Maize R2R3 Myb genes: sequence analysis reveals amplification in the higher plants.. Genetics.

[6] Ogata K, Morikawa S, Nakamura H, Hojo H, Yoshimura S (1995). Comparison of the free and DNA–complexed forms of the DNA–binding domain from c–Myb.. Nat Struct Biol.

[7] Paz–Ares J, Ghosal D, Wienand U, Peterson PA, Saedler H (1987). The regulatory c1 locus of maize encodes a protein with homology to myb proto–oncogene products and with structural similarities to transcriptional activators.. EMBO J.

[8] Durbarry A, Vizir I, Twell D (2005). Male germ line development in Arabidopsis: duo pollen mutants reveal gametophytic regulators of generative cell cycle progression.. Plant Physiol.

[9] Yanhui C, Xiaoyuan Y, Kun H, Meihua L, Jigang L (2006). The MYB transcription factor superfamily of Arabidopsis: expression analysis and phylogenetic comparison with the rice MYB family.. Plant Mol Biol.

[10] Wilkins O, Nahal H, Foong J, Provart NJ, Campbell MM (2009). Expansion and diversification of the Populus R2R3-MYB family of transcription factors.. Plant Physiol.

[11] Cai H, Tian S, Dong H (2011). Large Scale In Silico Identification of MYB Family Genes from Wheat Expressed Sequence Tags.. Mol Biotechnol [Epub ahead of print].

[12] Zhang L, Zhao G, Jia J, Liu X, Kong X (2012). Molecular characterization of 60 isolated wheat MYB genes and analysis of their expression during abiotic stress.. J Exp Bot.

[13] Jiang C, Gu X, Peterson T (2004). Identification of conserved gene structures and carboxy-terminal motifs in the Myb gene family of Arabidopsis and Oryza sativa L. ssp. indica.. Genome Biol.

[14] Matus JT, Aquea F, Arce–Johnson P (2008). Analysis of the grape MYB R2R3 subgroup reveals expanded wine quality–related clades and conserved gene structure organization across Vitis and Arabidopsis genomes.. BMC Plant Biology.

[15] Marocco A, Wissenbach M, Becker D, Paz-Ares J, Saedler H (1989). Multiple genes are transcribed in Hordeum vulgare and Zea mays that carry the DNA binding domain of the myb oncoproteins.. Mol Gen Genet.

[16] Grotewold E, Athma P, Peterson T (1991). Alternatively spliced products of the maize P gene encode proteins with homology to the DNA-binding domain of Myb-like transcription factors.. Proc Natl Acad Sci USA.

[17] Cone KC, Cocciolone SM, Burr FA, Burr B (1993). Maize anthocyanin regulatory gene pl is a duplicate of c1 that functions in the plant.. Plant Cell.

[18] Heine GF, Malik V, Dias AP, Grotewold E (2007). Expression and molecular characterization of ZmMYB-IF35 and related R2R3-MYB transcription factors.. Mol Biotechnol.

[19] Fornalé S, Sonbol FM, Maes T, Capellades M, Puigdomènech P (2006). Down-regulation of the maize and Arabidopsis thaliana caffeic acid O-methyl-transferase genes by two new maize R2R3-MYB transcription factors.. Plant Mol Biol.

[20] Palmer LE, Rabinowicz PD, O’Shaughnessy AL, Balija VS, Nascimento LU (2003). Maize genome sequencing by methylation filtration.. Science.

[21] Martienssen RA, Rabinowicz PD, O’Shaughnessy A, McCombie WR (2004). Sequencing the maize genome.. Curr Opin Plant Biol.

[22] Schnable PS, Ware D, Fulton RS, Stein JC, Wei F (2009). The B73 maize genome: complexity, diversity, and dynamics.. Science.

[23] Hegvold AB, Gabrielsen OS (1996). The importance of the linker connecting the repeats of the c-Myb oncoprotein may be due to a positioning function.. Nucleic Acids Res.

[24] Van Aalten DM, Grotewold E, Joshua-Tor L (1998). Essential dynamics from NMR clusters: dynamic properties of the Myb DNA-binding domain and a hinge-bending enhancing variant.. Methods.

[25] Williams CE, Grotewold E (1997). Differences between plant and animal Myb domains are fundamental for DNA binding activity, and chimeric Myb domains have novel DNA binding specificities.. J Biol Chem.

[26] Gigolashvili T, Yatusevich R, Berger B, Müller C, Flügge UI (2007). The R2R3–MYB transcription factor HAG1/MYB28 is a regulator of methionine–derived glucosinolate biosynthesis in Arabidopsis thaliana.. Plant J.

[27] Gigolashvili T, Engqvist M, Yatusevich R, Müller C, Flügge UI (2008). HAG2/MYB76 and HAG3/MYB29 exert a specific and coordinated control on the regulation of aliphatic glucosinolate biosynthesis in Arabidopsis thaliana.. New Phytol.

[28] Celenza JL, Quiel JA, Smolen GA, Merrikh H, Silvestro AR (2005). The Arabidopsis ATR1 Myb transcription factor controls indolic glucosinolate homeostasis.. Plant Physiol.

[29] Gigolashvili T, Berger B, Mock HP, Müller C, Weisshaar B (2007). The transcription factor HIG1/MYB51 regulates indolic glucosinolate biosynthesis in Arabidopsis thaliana.. Plant J.

[30] Sønderby IE, Hansen BG, Bjarnholt N, Ticconi C, Halkier BA (2007). A Systems Biology Approach Identifies a R2R3 MYB Gene Subgroup with Distinct and Overlapping Functions in Regulation of Aliphatic Glucosinolates.. PLoS One.

[31] Lee MM, Schiefelbein J (1999). WEREWOLF, a MYB-related protein in Arabidopsis, is a position-dependent regulator of epidermal cell patterning.. Cell.

[32] Tominaga R, Iwata M, Okada K, Wada T (2007). Functional analysis of the epidermal–specific MYB genes CAPRICE and WEREWOLF in Arabidopsis.. Plant Cell.

[33] Pu L, Li Q, Fan X, Yang W, Xue Y (2008). The R2R3 MYB transcription factor GhMYB109 is required for cotton fiber development.. Genetics.

[34] Ogata K, Morikawa S, Nakamura H, Sekikawa A, Inoue T (1994). Solution structure of a specific DNA complex of the Myb DNA-binding domain with cooperative recognition helices.. Cell.

[35] Heine GF, Hernandez JM, Grotewold E (2004). Two cysteines in plant R2R3 MYB domains participate in REDOX-dependent DNA binding.. J Biol Chem.

[36] Dias AP, Braun EL, McMullen MD, Grotewold E (2003). Recently duplicated maize R2R3 Myb genes provide evidence for distinct mechanisms of evolutionary divergence after duplication.. Plant Physiol.

[37] Sharp PA (1981). Speculations on RNA splicing.. Cell.

[38] Brett D, Pospisil H, Valcarcel J, Reich J, Bork P (2002). Alternative splicing and genome complexity. Nat.. Genet.

[39] Black DL (2003). Mechanisms of alternative pre-messenger RNA splicing.. Annu Rev Biochem.

[40] Matlin AJ, Clark F, Smith CW (2005). Understanding alternative splicing: towards a cellular code.. Nat Rev Mol Cell Biol.

[41] Mehrtens F, Kranz H, Bednarek P, Weisshaar B (2005). The Arabidopsis transcription factor MYB12 is a flavonol–specific regulator of phenylpropanoid biosynthesis.. Plant Physiol.

[42] Stracke R, Ishihara H, Huep G, Barsch A, Mehrtens F (2007). Differential regulation of closely related R2R3–MYB transcription factors controls flavonol accumulation in different parts of the Arabidopsis thaliana seedling.. Plant J.

[43] Czemmel S, Stracke R, Weisshaar B, Cordon N, Harris NN (2009). The grapevine R2R3-MYB transcription factor VvMYBF1 regulates flavonol synthesis in developing grape berries.. Plant Physiol.

[44] Ballester AR, Molthoff J, de Vos R, Hekkert BL, Orzaez D (2010). Biochemical and molecular analysis of pink tomatoes: deregulated expression of the gene encoding transcription factor SlMYB12 leads to pink tomato fruit color.. Plant Physiol.

[45] Boddu J, Jiang C, Sangar V, Olson T, Peterson T (2006). Comparative structural and functional characterization of sorghum and maize duplications containing orthologous myb transcription regulators of 3-deoxyflavonoid biosynthesis.. Plant Mol Biol.

[46] Zhang F, Peterson T (2005). Comparisons of maize pericarp color1 alleles reveal paralogous gene recombination and an organ-specific enhancer region.. Plant Cell.

[47] Nesi N, Jond C, Debeaujon I, Caboche M, Lepiniec L (2001). The Arabidopsis TT2 gene encodes an R2R3 MYB domain protein that acts as a key determinant for proanthocyanidin accumulation in developing seed.. Plant Cell.

[48] Pilu R, Piazza P, Petroni K, Ronchi A, Martin C (2003). pl-bol3, a complex allele of the anthocyanin regulatory pl1 locus that arose in a naturally occurring maize population.. Plant J.

[49] Mellway RD, Tran LT, Prouse MB, Campbell MM, Constabel CP (2009). The wound-, pathogen-, and ultraviolet B-responsive MYB134 gene encodes an R2R3 MYB transcription factor that regulates proanthocyanidin synthesis in poplar.. Plant Physiol.

[50] Akagi T, Ikegami A, Yonemori K (2010). DkMyb2 wound-induced transcription factor of persimmon (Diospyros kaki Thunb.), contributes to proanthocyanidin regulation.. Planta.

[51] Saitoh K, Onishi K, Mikami I, Thidar K, Sano Y (2004). Allelic diversification at the C (OsC1) locus of wild and cultivated rice: nucleotide changes associated with phenotypes.. Genetics.

[52] Jin H, Cominelli E, Bailey P, Parr A, Mehrtens F (2000). Transcriptional repression by AtMYB4 controls production of UV–protecting sunscreens in Arabidopsis.. EMBO J.

[53] Preston J, Wheeler J, Heazlewood J, Li SF, Parish RW (2004). AtMYB32 is required for normal pollen development in Arabidopsis thaliana.. Plant J.

[54] Legay S, Sivadon P, Blervacq AS, Pavy N, Baghdady A (2010). EgMYB1, an R2R3 MYB transcription factor from eucalyptus negatively regulates secondary cell wall formation in Arabidopsis and poplar.. New Phytol.

[55] Ma QH, Wang C, Zhu HH (2011). TaMYB4 cloned from wheat regulates lignin biosynthesis through negatively controlling the transcripts of both cinnamyl alcohol dehydrogenase and cinnamoyl-CoA reductase genes.. Biochimie.

[56] Colquhoun TA, Kim JY, Wedde AE, Levin LA, Schmitt KC (2011). PhMYB4 fine-tunes the floral volatile signature of Petunia x hybrida through PhC4H.. J Exp Bot.

[57] Sonbol FM, Fornale S, Capellades M, Encina A, Tourin S (2009). The maize ZmMYB42 represses the phenylpropanoid pathway and affects the cell wall structure, composition and degradability in Arabidopsis thaliana.. Plant Mol Biol.

[58] Waites R, Selvadurai HR, Oliver IR, Hudson A (1998). The PHANTASTICA gene encodes a MYB transcription factor involved in growth and dorsoventrality of lateral organs in Antirrhinum.. Cell.

[59] Koltai H, Bird DM (2000). Epistatic repression of PHANTASTICA and class 1 KNOTTED genes is uncoupled in tomato.. Plant J.

[60] Morimoto R, Nishioka E, Murai K, Takumi S (2009). Functional conservation of wheat orthologs of maize rough sheath1 and rough sheath2 genes.. Plant Mol Biol.

[61] Tsiantis M, Schneeberger R, Golz JF, Freeling M, Langdale JA (1999). The maize rough sheath2 gene and leaf development programs in monocot and dicot plants.. Science.

[62] Byrne ME, Barley R, Curtis M, Arroyo JM, Dunham M (2000). Asymmetric leaves1 mediates leaf patterning and stem cell function in Arabidopsis.. Nature.

[63] Schmitz G, Tillmann E, Carriero F, Fiore C, Cellini F (2002). The tomato Blind gene encodes a MYB transcription factor that controls the formation of lateral meristems.. Proc Natl Acad Sci USA.

[64] Keller T, Abbott J, Moritz T, Doerner P (2006). Arabidopsis REGULATOR OF AXILLARY MERISTEMS1 controls a leaf axil stem cell niche and modulates vegetative development.. Plant Cell.

[65] Müller D, Schmitz G, Theres K (2006). Blind homologous R2R3 Myb genes control the pattern of lateral meristem initiation in Arabidopsis.. Plant Cell.

[66] Feng C, Andreasson E, Maslak A, Mock HP, Mattsson O (2004). Arabidopsis MYB68 in development and responses to environmental cues.. Plant Science.

[67] Noda K, Glover BJ, Linstead P, Martin C (1994). Flower colour intensity depends on specialized cell shape controlled by a Myb-related transcription factor.. Nature.

[68] Perez-Rodriguez M, Jaffe FW, Butelli E, Glover BJ, Martin C (2005). Development of three different cell types is associated with the activity of a specific MYB transcription factor in the ventral petal of Antirrhinum majus flowers.. Development.

[69] Baumann K, Perez–Rodriguez M, Bradley D, Venail J, Bailey P (2007). Control of cell and petal morphogenesis by R2R3 MYB transcription factors.. Development.

[70] Jaffé FW, Tattersall A, Glover BJ (2007). A truncated MYB transcription factor from Antirrhinum majus regulates epidermal cell outgrowth.. J Exp Bot.

[71] Jakoby MJ, Falkenhan D, Mader MT, Brininstool G, Wischnitzki E (2008). Transcriptional profiling of mature Arabidopsis trichomes reveals that NOECK encodes the MIXTA-like transcriptional regulator MYB106.. Plant Physiol.

[72] Woodger FJ, Gubler F, Pogson BJ, Jacobsen JV (2003). A Mak-like kinase is a repressor of GAMYB in barley aleurone.. Plant J.

[73] Gubler F, Kalla R, Roberts JK, Jacobsen JV (1995). Gibberellin-regulated expression of a myb gene in barley aleurone cells: evidence for Myb transactivation of a high-pI alpha-amylase gene promoter.. Plant Cell.

[74] Gocal GF, Poole AT, Gubler F, Watts RJ, Blundell C (1999). Long-day up-regulation of a GAMYB gene during Lolium temulentum inflorescence formation.. Plant Physiol.

[75] Tsuji H, Aya K, Ueguchi-Tanaka M, Shimada Y, Nakazono M (2006). GAMYB controls different sets of genes and is differentially regulated by microRNA in aleurone cells and anthers.. Plant J.

[76] Aya K, Ueguchi-Tanaka M, Kondo M, Hamada K, Yano K (2009). Gibberellin modulates anther development in rice via the transcriptional regulation of GAMYB.. Plant Cell.

[77] Gocal GF, Sheldon CC, Gubler F, Moritz T, Bagnall DJ (2001). GAMYB–like genes, flowering, and gibberellin signaling in Arabidopsis.. Plant Physiol.

[78] Murray F, Kalla R, Jacobsen J, Gubler F (2003). A role for HvGAMYB in anther development.. Plant J.

[79] Kaneko M, Inukai Y, Ueguchi-Tanaka M, Itoh H, Izawa T (2004). Loss-of-function mutations of the rice GAMYB gene impair alpha-amylase expression in aleurone and flower development.. Plant Cell.

[80] Gong X, Bewley DJ (2008). A GAMYB-like gene in tomato and its expression during seed germination.. Planta.

[81] Gocal GF, Poole AT, Gubler F, Watts RJ, Blundell C (1999). Long-day up-regulation of a GAMYB gene during Lolium temulentum inflorescence formation.. Plant Physiol.

[82] Millar AA, Gubler F (2005). The Arabidopsis GAMYB-like genes, MYB33 and MYB65, are microRNA-regulated genes that redundantly facilitate anther development.. Plant Cell.

[83] Rhoades MW, Reinhart BJ, Lim LP, Burge CB, Bartel B (2002). Prediction of plant microRNA targets.. Cell.

[84] Achard P, Herr A, Baulcombe DC, Harberd NP (2004). Modulation of floral development by a gibberellin-regulated micro-RNA.. Development.

[85] Zhang L, Chia JM, Kumari S, Stein JC, Liu Z (2009). A genome-wide characterization of microRNA genes in maize.. PLoS Genet.

[86] Addo-Quaye C, Eshoo TW, Bartel DP, Axtell MJ (2008). Endogenous siRNA and miRNA targets identified by sequencing of the Arabidopsis degradome.. Current Biology.

[87] Grotewold E, Sainz MB, Tagliani L, Hernandez JM, Bowen B (2000). Identification of the residues in the Myb domain of maize C1 that specify the interaction with the bHLH cofactor R. Proc Natl Acad Sci USA.

[88] Tohge T, Nishiyama Y, Hirai MY, Yano M, Nakajima J (2005a). Functional genomics by integrated analysis of metabolome and transcriptome of Arabidopsis plants over-expressing an MYB transcription factor.. Plant J.

[89] Li SF, Milliken ON, Pham H, Seyit R, Napoli R (2009). The Arabidopsis MYB5 transcription factor regulates mucilage synthesis, seed coat development, and trichome morphogenesis.. Plant Cell.

[90] Payne CT, Zhang F, Lloyd AM (2000). GL3 encodes a bHLH protein that regulates trichome development in Arabidopsis through interaction with GL1 and TTG1.. Genetics.

[91] Zhang F, Gonzalez A, Zhao M, Payne CT, Lloyd A (2003). A network of redundant bHLH proteins functions in all TTG1-dependent pathways of Arabidopsis.. Development.

[92] Walker AR, Davison PA, Bolognesi-Winfield AC, James CM, Srinivasan N (1999). The Transparent Testa Glabra1 locus, which regulates trichome differentiation and anthocyanin biosynthesis in Arabidopsis, encodes aWD40 repeat protein.. Plant Cell.

[93] Zimmermann IM, Heim MA, Weisshaar B, Uhrig JF (2004). Comprehensive identification of Arabidopsis thaliana MYB transcription factors interacting with R/B-like BHLH proteins.. Plant J.

[94] Dubos C, Le Gourrierec J, Baudry A, Huep G, Lanet E (2008). MYBL2 is a new regulator of flavonoid biosynthesis in Arabidopsis thaliana.. Plant J.

[95] Apweiler R, Attwood TK, Bairoch A, Bateman A, Birney E (2001). The InterPro database, an integrated documentation resource for protein families, domains and functional sites.. Nucleic Acids Res.

[96] Letunic I, Copley RR, Schmidt S, Ciccarelli FD, Doerks T (2004). SMART 4.0: towards genomic data integration.. Nucleic Acids Res.

[97] Thompson JD, Gibson TJ, Plewniak F, Jeanmougin F, Higgins DG (1997). The CLUSTAL_X windows interface: flexible strategies for multiple sequence alignment aided by quality analysis tools.. Nucleic Acids Res.

[98] Nicholas KB, Nicholas HBJ, Deerfield DWI (1997). Genedoc: analysis and visualization of genetic variation.. Embnew News.

[99] Bailey TL, Williams N, Misleh C, Li WW (2006). MEME: discovering and analyzing DNA and protein sequence motifs.. Nucleic Acids Res.

[100] Bailey TL, Gribskov M (1998). Combining evidence using p–values: application to sequence homology searches.. Bioinformatics.

[101] Guindon S, Gascuel O (2003). A simple, fast, and accurate algorithm to estimate large phylogenies by maximum likelihood. Syst. Biol..

