# Supplementary material for: The R2R3-MYB Transcription Factor Gene Family in Maize
Source: PLoS One. 2012 Jun 7;7(6):e37463. doi: 10.1371/journal.pone.0037463 (PMC3370817; doi:10.1371/journal.pone.0037463)
Supplement: Figure S1 — Sequence comparison of the DNA-binding domains in maize and Arabidopsis R2R3-MYB genes. (PDF) [file pone.0037463.s001.pdf]

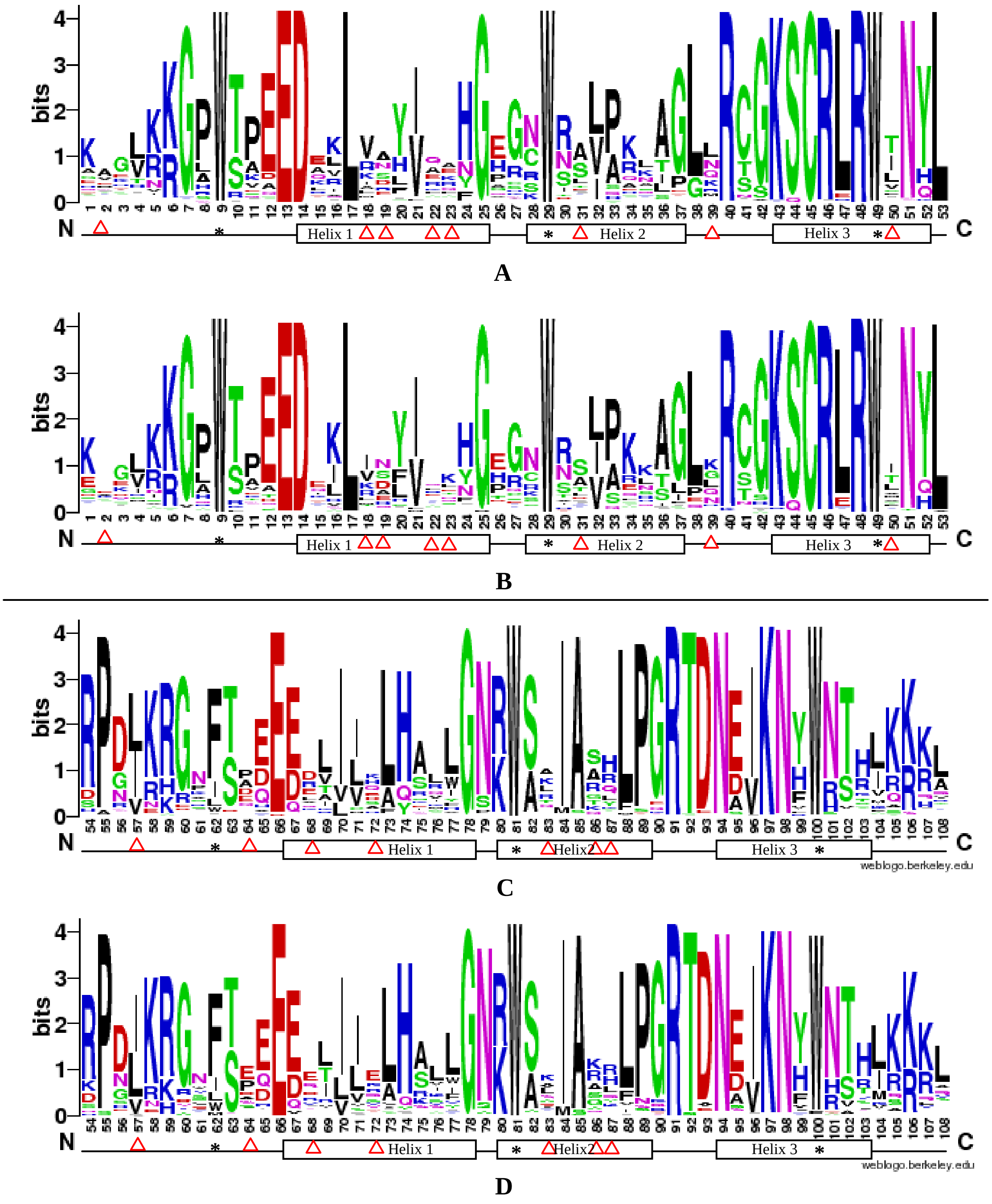

Figure S1. Sequence comparison of the DNA-binding domains in maize and Arabidopsis R2R3-MYB genes. The first part includes sequence logos of the R2 in maize (A) and *Arabidopsis* (B), while second part is the R3 in maize (C) and *Arabidopsis* (D), respectively. MYB repeats are based on full-length alignments of all R2R3-MYB domains in these two species. The bit score indicates the information content for each position in the sequence. The position of the three α -helices that form each MYB repeat are marked (Helix 1 to 3). The asterisks indicate the conserved tryptophan residues (W) in the MYB domain, whereas the residues that are different between maize and *Arabidopsis* MYB domain are marked with triangle marker.
