# Supplementary material for: The R2R3-MYB Transcription Factor Gene Family in Maize
Source: PLoS One. 2012 Jun 7;7(6):e37463. doi: 10.1371/journal.pone.0037463 (PMC3370817; doi:10.1371/journal.pone.0037463)
Supplement: Figure S2 — Phylogenetic tree of 157 typical maize R2R3-MYB family genes. (PDF) [file pone.0037463.s002.pdf]

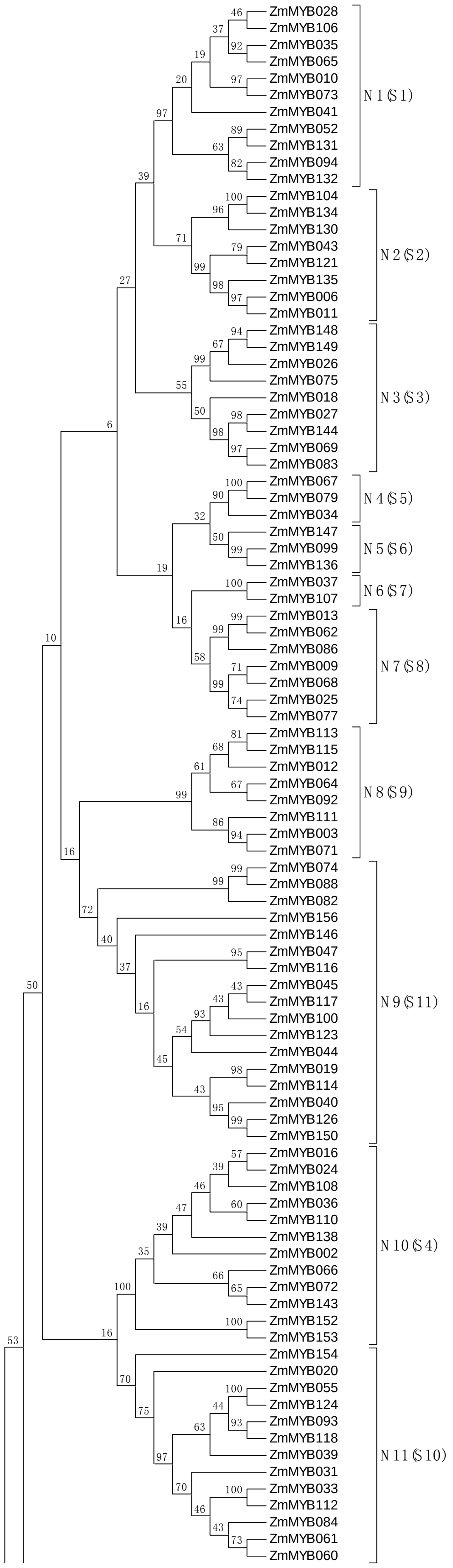
ZmMYB028
ZmMYB106
ZmMYB035
ZmMYB065
ZmMYB010
ZmMYB073
ZmMYB041
ZmMYB052
ZmMYB131
ZmMYB094
ZmMYB132
N 1(S 1)
ZmMYB104
ZmMYB134
ZmMYB130
ZmMYB043
ZmMYB121
ZmMYB135
ZmMYB006
ZmMYB011
N 2(S 2)
ZmMYB148
ZmMYB149
ZmMYB026
ZmMYB075
ZmMYB018
ZmMYB027
ZmMYB144
ZmMYB069
ZmMYB083
N 3(S 3)
ZmMYB067
ZmMYB079
ZmMYB034
N 4(S 5)
ZmMYB147
ZmMYB099
ZmMYB136
N 5(S 6)
ZmMYB037
ZmMYB107
N 6(S 7)
ZmMYB013
ZmMYB062
ZmMYB086
ZmMYB009
ZmMYB068
ZmMYB025
ZmMYB077
N 7(S 8)
ZmMYB113
ZmMYB115
ZmMYB012
ZmMYB064
ZmMYB092
ZmMYB111
ZmMYB003
ZmMYB071
N 8(S 9)
ZmMYB074
ZmMYB088
ZmMYB082
ZmMYB156
ZmMYB146
ZmMYB047
ZmMYB116
ZmMYB045
ZmMYB117
ZmMYB100
ZmMYB123
ZmMYB044
ZmMYB019
ZmMYB114
ZmMYB040
ZmMYB126
ZmMYB150
N 9(S 11)
ZmMYB016
ZmMYB024
ZmMYB108
ZmMYB036
ZmMYB110
ZmMYB138
ZmMYB002
ZmMYB066
ZmMYB072
ZmMYB143
ZmMYB152
ZmMYB153
N 10(S 4)
ZmMYB154
ZmMYB020
ZmMYB055
ZmMYB124
ZmMYB093
ZmMYB118
ZmMYB039
ZmMYB031
ZmMYB033
ZmMYB112
ZmMYB084
ZmMYB061
ZmMYB060
N 11(S 10)

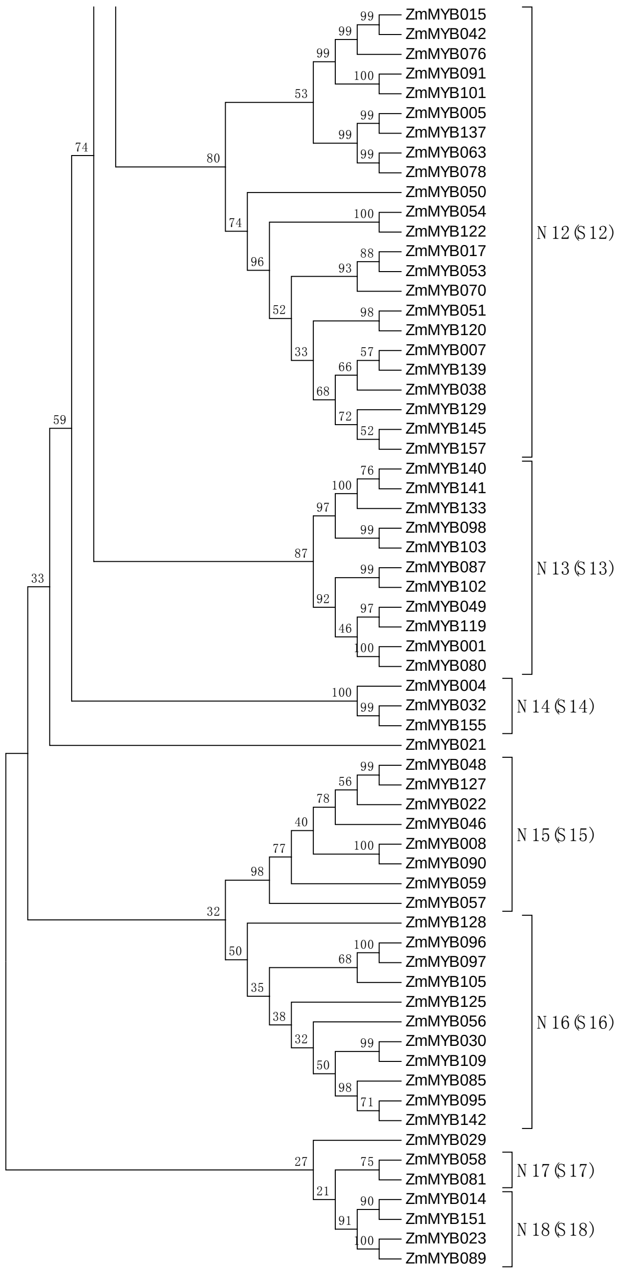

Figure S2. Phylogenetic tree of 157 typical maize R2R3-MYB family genes.

The unrooted tree was inferred by the neighbor-joining method after the alignment of the whole amino acid sequences of the 157 typical maize R2R3-MYB genes listed in Table S1. The MYB proteins are clustered into 18 subgroups (designated as N1 to N18) and the corresponding subgroups in NJ tree (Fig.2) were listed in round bracket, for reference.
