# Supplementary material for: The R2R3-MYB Transcription Factor Gene Family in Maize
Source: PLoS One. 2012 Jun 7;7(6):e37463. doi: 10.1371/journal.pone.0037463 (PMC3370817; doi:10.1371/journal.pone.0037463)
Supplement: Figure S3 — ML phylogenetic tree of 157 typical maize R2R3-MYB family genes. (PDF) [file pone.0037463.s003.pdf]

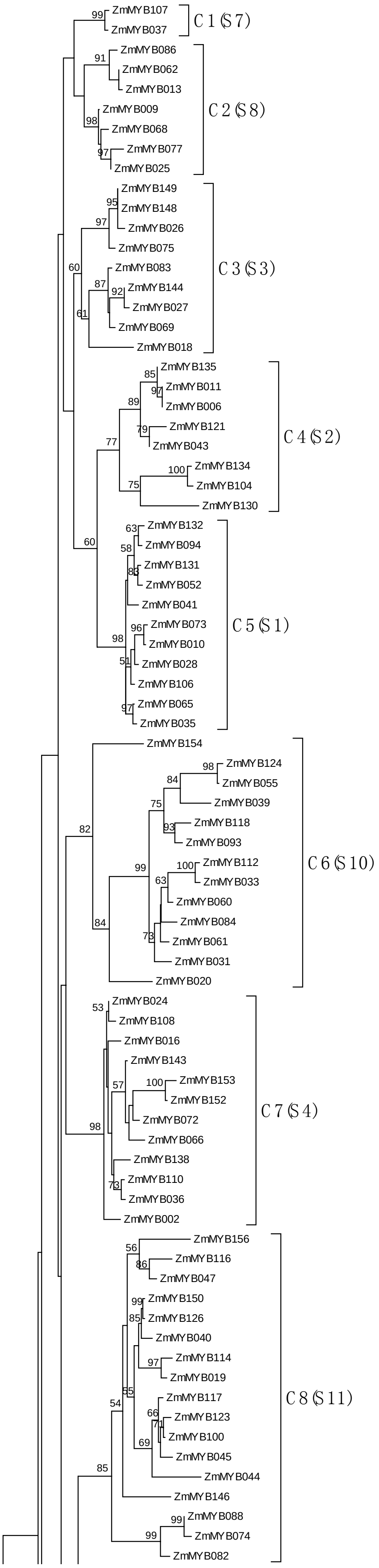

ZmMYB107
ZmMYB037
C1(S7)
ZmMYB086
ZmMYB062
ZmMYB013
ZmMYB009
ZmMYB068
ZmMYB077
ZmMYB025
C2(S8)
ZmMYB149
ZmMYB148
ZmMYB026
ZmMYB075
ZmMYB083
ZmMYB144
ZmMYB027
ZmMYB069
ZmMYB018
C3(S3)
ZmMYB135
ZmMYB011
ZmMYB006
ZmMYB121
ZmMYB043
ZmMYB134
ZmMYB104
ZmMYB130
C4(S2)
ZmMYB132
ZmMYB094
ZmMYB131
ZmMYB052
ZmMYB041
ZmMYB073
ZmMYB010
ZmMYB028
ZmMYB106
ZmMYB065
ZmMYB035
C5(S1)
ZmMYB154
ZmMYB124
ZmMYB055
ZmMYB039
ZmMYB118
ZmMYB093
ZmMYB112
ZmMYB033
ZmMYB060
ZmMYB084
ZmMYB061
ZmMYB031
ZmMYB020
C6(S10)
ZmMYB024
ZmMYB108
ZmMYB016
ZmMYB143
ZmMYB153
ZmMYB152
ZmMYB072
ZmMYB066
ZmMYB138
ZmMYB110
ZmMYB036
ZmMYB002
C7(S4)
ZmMYB156
ZmMYB116
ZmMYB047
ZmMYB150
ZmMYB126
ZmMYB040
ZmMYB114
ZmMYB019
ZmMYB117
ZmMYB123
ZmMYB100
ZmMYB045
ZmMYB044
ZmMYB146
ZmMYB088
ZmMYB074
ZmMYB082
C8(S11)

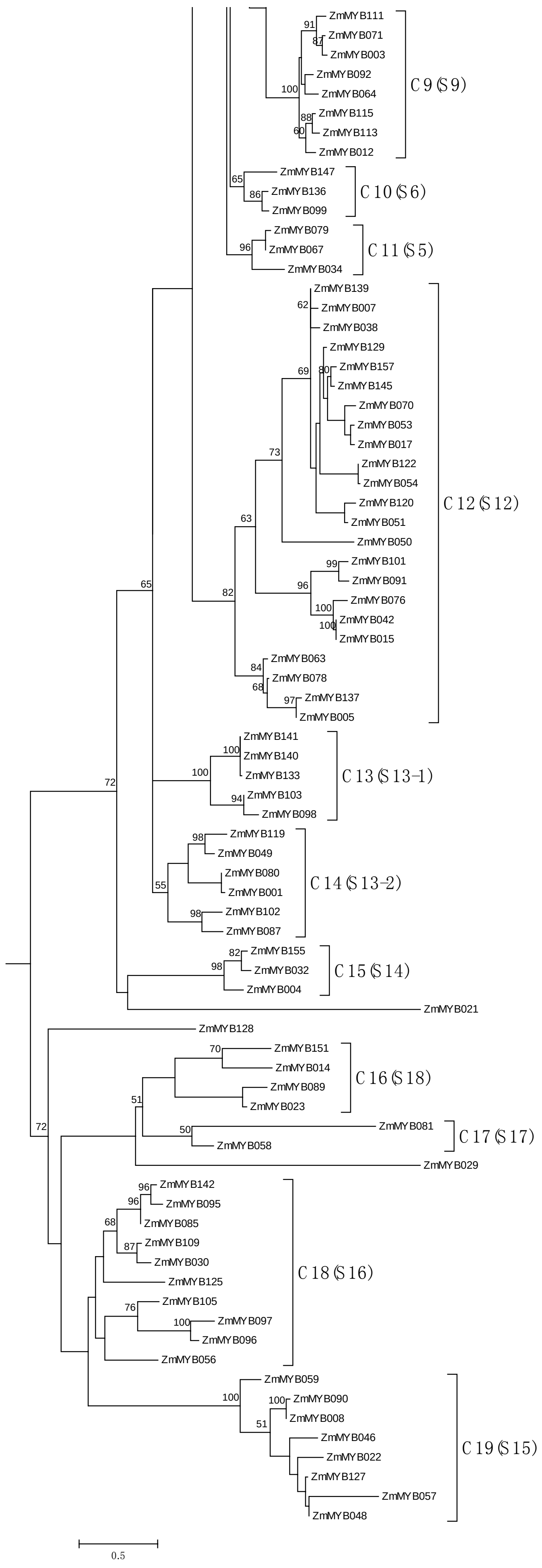
ZmMYB111
ZmMYB071
ZmMYB003
ZmMYB092
ZmMYB064
ZmMYB115
ZmMYB113
ZmMYB012
C9 (S9)
ZmMYB147
ZmMYB136
ZmMYB099
C10 (S6)
ZmMYB079
ZmMYB067
ZmMYB034
C11 (S5)
ZmMYB139
ZmMYB007
ZmMYB038
ZmMYB129
ZmMYB157
ZmMYB145
ZmMYB070
ZmMYB053
ZmMYB017
ZmMYB122
ZmMYB054
ZmMYB120
ZmMYB051
ZmMYB050
ZmMYB101
ZmMYB091
ZmMYB076
ZmMYB042
ZmMYB015
ZmMYB063
ZmMYB078
ZmMYB137
ZmMYB005
C12 (S12)
ZmMYB141
ZmMYB140
ZmMYB133
ZmMYB103
ZmMYB098
C13 (S13-1)
ZmMYB119
ZmMYB049
ZmMYB080
ZmMYB001
ZmMYB102
ZmMYB087
C14 (S13-2)
ZmMYB155
ZmMYB032
ZmMYB004
C15 (S14)
ZmMYB021
ZmMYB128
ZmMYB151
ZmMYB014
ZmMYB089
ZmMYB023
C16 (S18)
ZmMYB081
ZmMYB058
C17 (S17)
ZmMYB029
ZmMYB142
ZmMYB095
ZmMYB085
ZmMYB109
ZmMYB030
ZmMYB125
ZmMYB105
ZmMYB097
ZmMYB096
ZmMYB056
C18 (S16)
ZmMYB059
ZmMYB090
ZmMYB008
ZmMYB046
ZmMYB022
ZmMYB127
ZmMYB057
ZmMYB048
C19 (S15)
0.5

Figure S3. ML phylogenetic tree of 157 typical maize R2R3-MYB family genes. The unrooted tree was constructed by the maximum likelihood (ML) method based on the alignment of the corresponding MYB DNA binding domains. Boot strapping values are indicated as percentages along the branches in ML analysis. The MYB proteins are clustered into 19 subgroups (designated as C1 to C19) and the corresponding subgroups in NJ tree (Fig.2) were listed in round bracket, for reference.
