# Supplementary material for: The R2R3-MYB Transcription Factor Gene Family in Maize
Source: PLoS One. 2012 Jun 7;7(6):e37463. doi: 10.1371/journal.pone.0037463 (PMC3370817; doi:10.1371/journal.pone.0037463)
Supplement: Figure S4 — Phylogenetic tree of the R2R3-MYB proteins from maize (Zm), Arabidopsis (At) and other plants. (PDF) [file pone.0037463.s004.pdf]

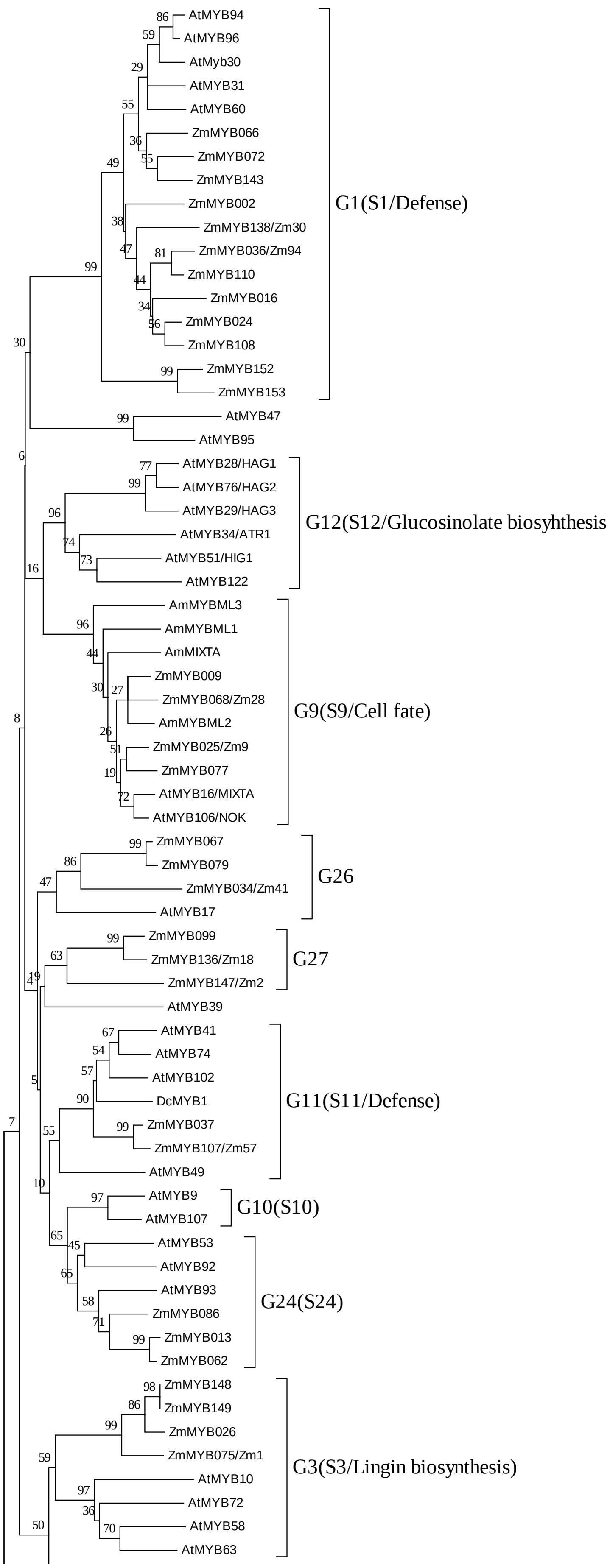
AtMYB94
AtMYB96
AtMyb30
AtMYB31
AtMYB60
ZmMYB066
ZmMYB072
ZmMYB143
ZmMYB002
ZmMYB138/Zm30
ZmMYB036/Zm94
ZmMYB110
ZmMYB016
ZmMYB024
ZmMYB108
ZmMYB152
ZmMYB153
G1(S1/Defense)
AtMYB47
AtMYB95
AtMYB28/HAG1
AtMYB76/HAG2
AtMYB29/HAG3
AtMYB34/ATR1
AtMYB51/HIG1
AtMYB122
G12(S12/Glucosinolate biosyhthesis
AmMYBML3
AmMYBML1
AmMIXTA
ZmMYB009
ZmMYB068/Zm28
AmMYBML2
ZmMYB025/Zm9
ZmMYB077
AtMYB16/MIXTA
AtMYB106/NOK
G9(S9/Cell fate)
ZmMYB067
ZmMYB079
ZmMYB034/Zm41
AtMYB17
G26
ZmMYB099
ZmMYB136/Zm18
ZmMYB147/Zm2
AtMYB39
G27
AtMYB41
AtMYB74
AtMYB102
DcMYB1
ZmMYB037
ZmMYB107/Zm57
AtMYB49
G11(S11/Defense)
AtMYB9
AtMYB107
G10(S10)
AtMYB53
AtMYB92
AtMYB93
ZmMYB086
ZmMYB013
ZmMYB062
G24(S24)
ZmMYB148
ZmMYB149
ZmMYB026
ZmMYB075/Zm1
AtMYB10
AtMYB72
AtMYB58
AtMYB63
G3(S3/Lingin biosynthesis)

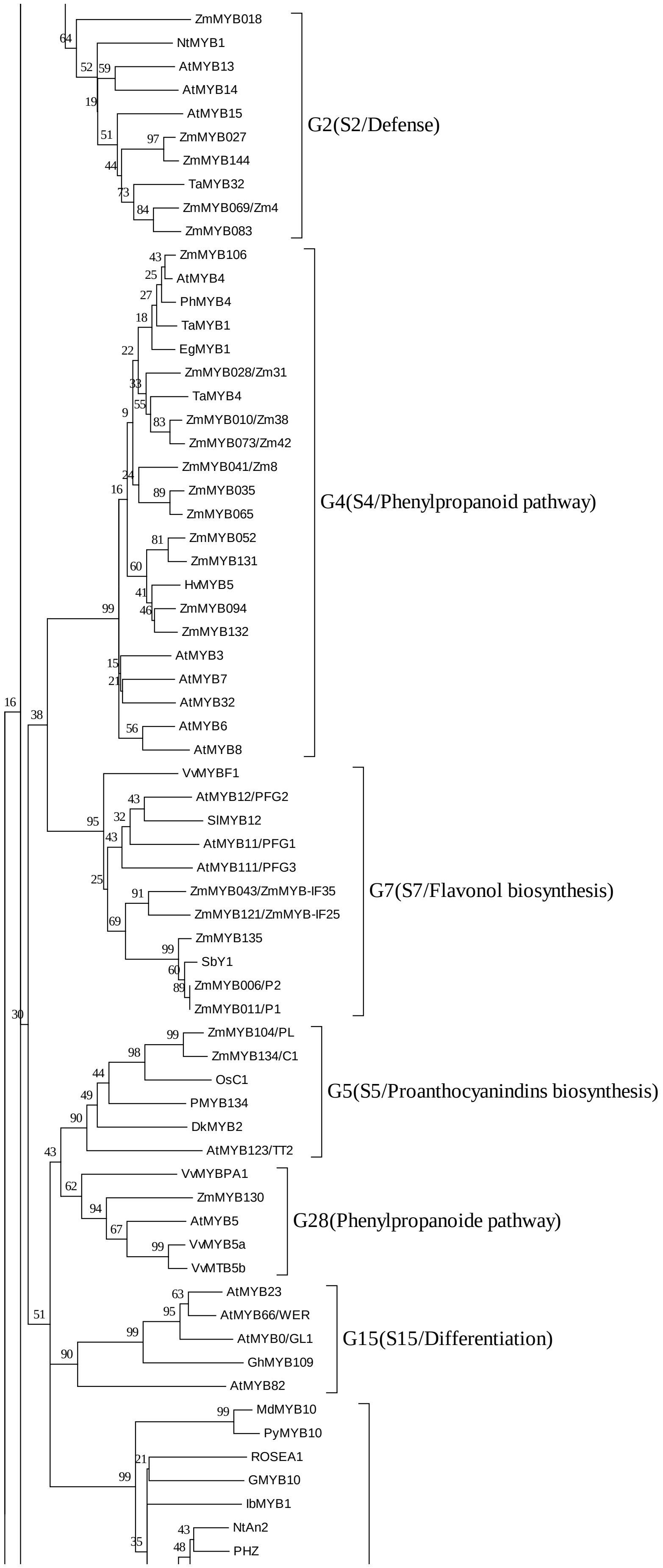
ZmMYB018
NtMYB1
AtMYB13
AtMYB14
AtMYB15
ZmMYB027
ZmMYB144
TaMYB32
ZmMYB069/Zm4
ZmMYB083
G2(S2/Defense)
ZmMYB106
AtMYB4
PhMYB4
TaMYB1
EgMYB1
ZmMYB028/Zm31
TaMYB4
ZmMYB010/Zm38
ZmMYB073/Zm42
ZmMYB041/Zm8
ZmMYB035
ZmMYB065
ZmMYB052
ZmMYB131
HvMYB5
ZmMYB094
ZmMYB132
AtMYB3
AtMYB7
AtMYB32
AtMYB6
AtMYB8
G4(S4/Phenylpropanoid pathway)
VvMYBF1
AtMYB12/PFG2
SlMYB12
AtMYB11/PFG1
AtMYB111/PFG3
ZmMYB043/ZmMYB-IF35
ZmMYB121/ZmMYB-IF25
ZmMYB135
SbY1
ZmMYB006/P2
ZmMYB011/P1
G7(S7/Flavonol biosynthesis)
ZmMYB104/PL
ZmMYB134/C1
OsC1
PMYB134
DkMYB2
AtMYB123/TT2
G5(S5/Proanthocyanindins biosynthesis)
VvMYBPA1
ZmMYB130
AtMYB5
VvMYB5a
VvMTB5b
G28(Phenylpropanoide pathway)
AtMYB23
AtMYB66/WER
AtMYB0/GL1
GhMYB109
AtMYB82
G15(S15/Differentiation)
MdMYB10
PyMYB10
ROSEA1
GMYB10
IbMYB1
NtAn2
PHZ

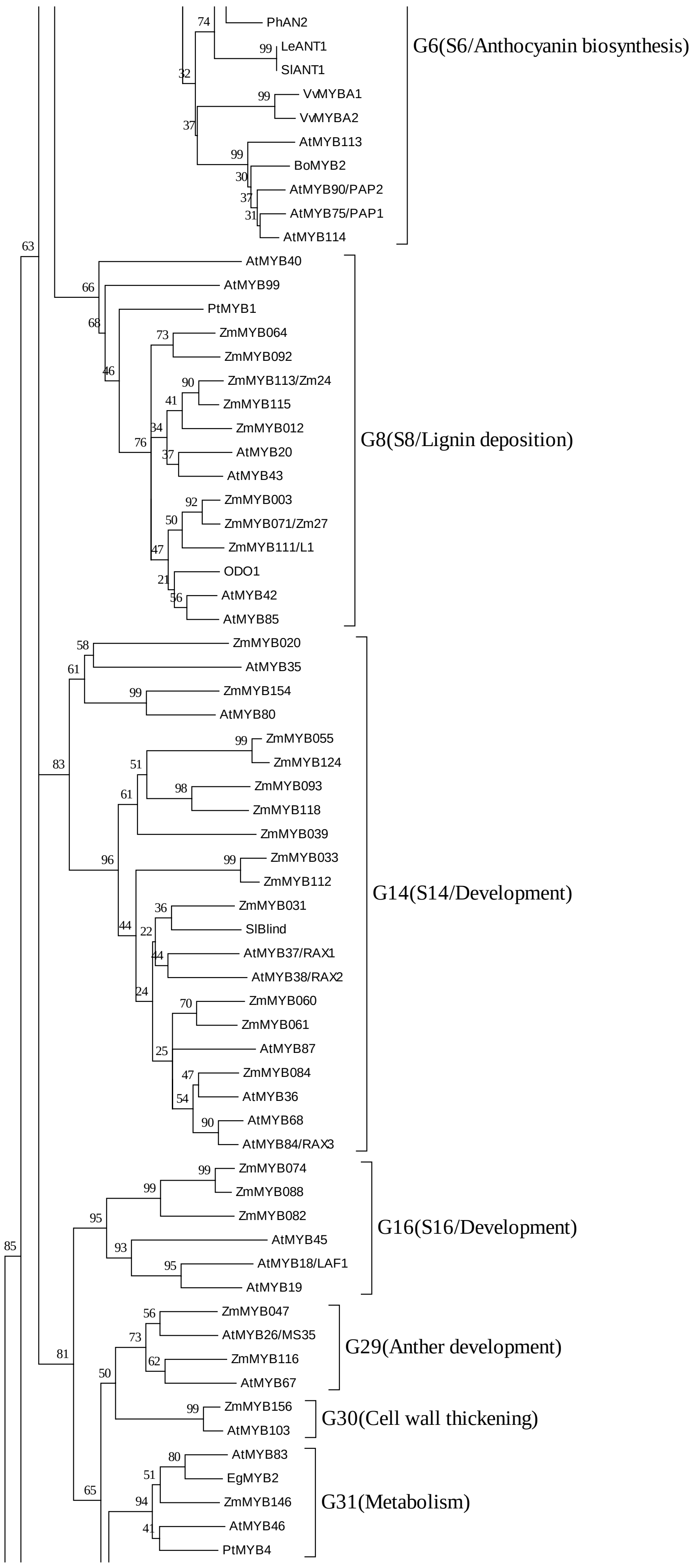
PhAN2
LeANT1
SlANT1
VvMYBA1
VvMYBA2
AtMYB113
BoMYB2
AtMYB90/PAP2
AtMYB75/PAP1
AtMYB114
G6(S6/Anthocyanin biosynthesis)
AtMYB40
AtMYB99
PtMYB1
ZmMYB064
ZmMYB092
ZmMYB113/Zm24
ZmMYB115
ZmMYB012
AtMYB20
AtMYB43
ZmMYB003
ZmMYB071/Zm27
ZmMYB111/L1
ODO1
AtMYB42
AtMYB85
G8(S8/Lignin deposition)
ZmMYB020
AtMYB35
ZmMYB154
AtMYB80
ZmMYB055
ZmMYB124
ZmMYB093
ZmMYB118
ZmMYB039
ZmMYB033
ZmMYB112
ZmMYB031
SlBlind
AtMYB37/RAX1
AtMYB38/RAX2
ZmMYB060
ZmMYB061
AtMYB87
ZmMYB084
AtMYB36
AtMYB68
AtMYB84/RAX3
G14(S14/Development)
ZmMYB074
ZmMYB088
ZmMYB082
AtMYB45
AtMYB18/LAF1
AtMYB19
G16(S16/Development)
ZmMYB047
AtMYB26/MS35
ZmMYB116
AtMYB67
G29(Anther development)
ZmMYB156
AtMYB103
G30(Cell wall thickening)
AtMYB83
EgMYB2
ZmMYB146
AtMYB46
PtMYB4
G31(Metabolism)

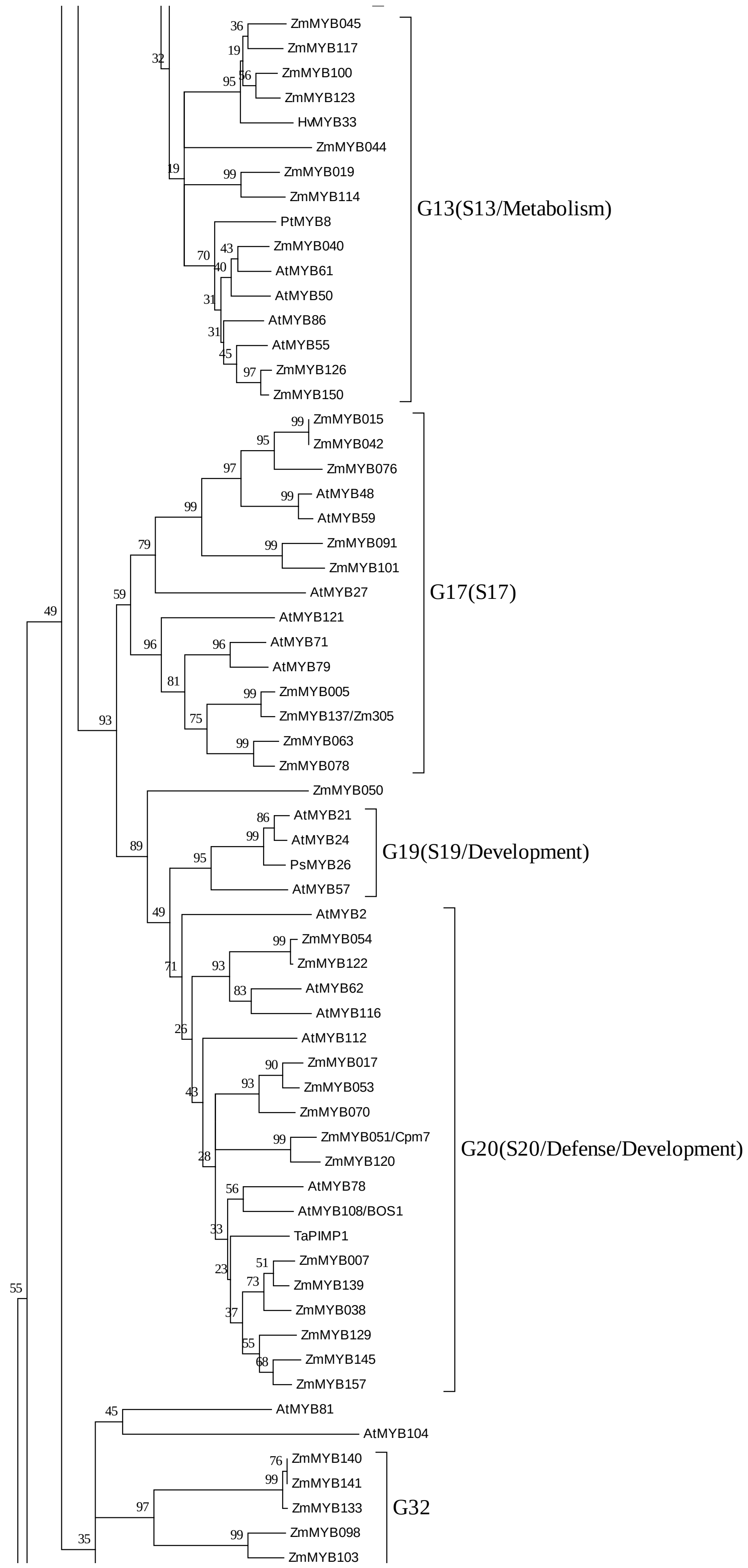
ZmMYB045
ZmMYB117
ZmMYB100
ZmMYB123
HvMYB33
ZmMYB044
ZmMYB019
ZmMYB114
PtMYB8
ZmMYB040
AtMYB61
AtMYB50
AtMYB86
AtMYB55
ZmMYB126
ZmMYB150
G13(S13/Metabolism)
ZmMYB015
ZmMYB042
ZmMYB076
AtMYB48
AtMYB59
ZmMYB091
ZmMYB101
AtMYB27
AtMYB121
AtMYB71
AtMYB79
ZmMYB005
ZmMYB137/Zm305
ZmMYB063
ZmMYB078
G17(S17)
ZmMYB050
AtMYB21
AtMYB24
PsMYB26
AtMYB57
G19(S19/Development)
AtMYB2
ZmMYB054
ZmMYB122
AtMYB62
AtMYB116
AtMYB112
ZmMYB017
ZmMYB053
ZmMYB070
ZmMYB051/Cpm7
ZmMYB120
AtMYB78
AtMYB108/BOS1
TaPIMP1
ZmMYB007
ZmMYB139
ZmMYB038
ZmMYB129
ZmMYB145
ZmMYB157
G20(S20/Defense/Development)
AtMYB81
AtMYB104
ZmMYB140
ZmMYB141
ZmMYB133
ZmMYB098
ZmMYB103
G32

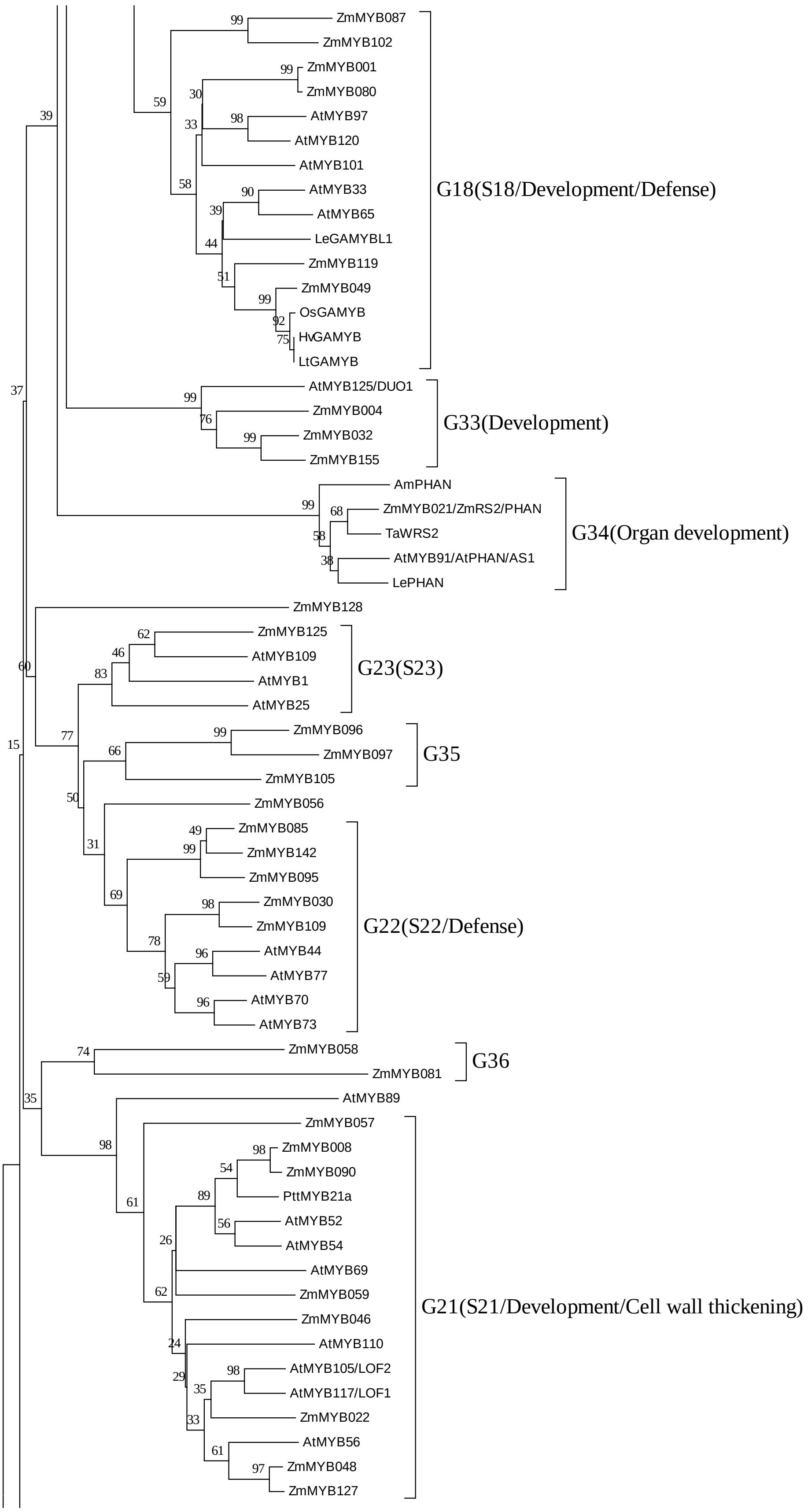
ZmMYB087
ZmMYB102
ZmMYB001
ZmMYB080
AtMYB97
AtMYB120
AtMYB101
AtMYB33
AtMYB65
LeGAMYBL1
ZmMYB119
ZmMYB049
OsGAMYB
HvGAMYB
LtGAMYB
G18(S18/Development/Defense)
AtMYB125/DUO1
ZmMYB004
ZmMYB032
ZmMYB155
G33(Development)
AmPHAN
ZmMYB021/ZmRS2/PHAN
TaWRS2
AtMYB91/AtPHAN/AS1
LePHAN
G34(Organ development)
ZmMYB128
ZmMYB125
AtMYB109
AtMYB1
AtMYB25
G23(S23)
ZmMYB096
ZmMYB097
ZmMYB105
G35
ZmMYB056
ZmMYB085
ZmMYB142
ZmMYB095
ZmMYB030
ZmMYB109
AtMYB44
AtMYB77
AtMYB70
AtMYB73
G22(S22/Defense)
ZmMYB058
ZmMYB081
G36
AtMYB89
ZmMYB057
ZmMYB008
ZmMYB090
PttMYB21a
AtMYB52
AtMYB54
AtMYB69
ZmMYB059
ZmMYB046
AtMYB110
AtMYB105/LOF2
AtMYB117/LOF1
ZmMYB022
AtMYB56
ZmMYB048
ZmMYB127
G21(S21/Development/Cell wall thickening)

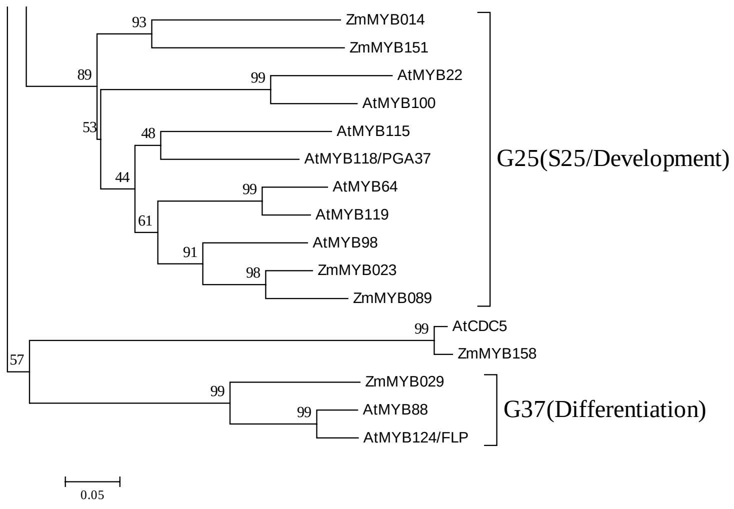

Figure S4. Phylogenetic tree of the R2R3-MYB proteins from maize (Zm), *Arabidopsis* (At) and other plants. The neighbor-joining tree includes 158 R2R3-MYB proteins from maize, and 126 from *Arabidopsis*, and a further 52 from other plant species. The proteins are clustered into 37 subgroups (triangles), designated with a subgroup number (e.g. G1). The bootstrap values lower than 50 are not shown in the phylogenetic tree. Five proteins did not fit well into clusters.
