# Supplementary material for: The R2R3-MYB Transcription Factor Gene Family in Maize
Source: PLoS One. 2012 Jun 7;7(6):e37463. doi: 10.1371/journal.pone.0037463 (PMC3370817; doi:10.1371/journal.pone.0037463)
Supplement: Figure S5 — Maximum likelihood (ML) tree of the R2R3-MYB proteins from maize (Zm), Arabidopsis (At) and other plants. (PDF) [file pone.0037463.s005.pdf]

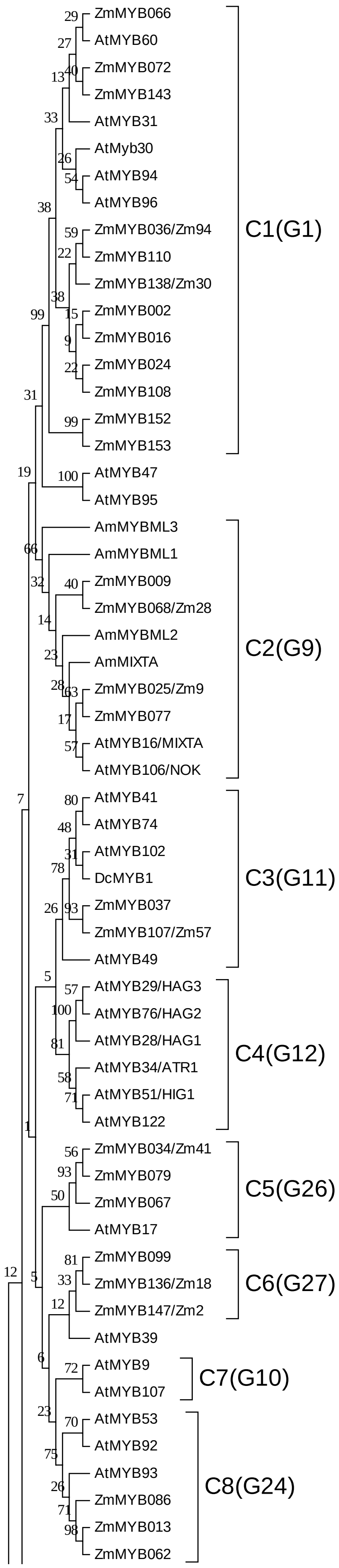

ZmMYB066
AtMYB60
ZmMYB072
ZmMYB143
AtMYB31
AtMyb30
AtMYB94
AtMYB96
ZmMYB036/Zm94
ZmMYB110
ZmMYB138/Zm30
ZmMYB002
ZmMYB016
ZmMYB024
ZmMYB108
ZmMYB152
ZmMYB153
C1(G1)
AtMYB47
AtMYB95
AmMYBML3
AmMYBML1
ZmMYB009
ZmMYB068/Zm28
AmMYBML2
AmMIXTA
ZmMYB025/Zm9
ZmMYB077
AtMYB16/MIXTA
AtMYB106/NOK
C2(G9)
AtMYB41
AtMYB74
AtMYB102
DcMYB1
ZmMYB037
ZmMYB107/Zm57
AtMYB49
C3(G11)
AtMYB29/HAG3
AtMYB76/HAG2
AtMYB28/HAG1
AtMYB34/ATR1
AtMYB51/HIG1
AtMYB122
C4(G12)
ZmMYB034/Zm41
ZmMYB079
ZmMYB067
AtMYB17
C5(G26)
ZmMYB099
ZmMYB136/Zm18
ZmMYB147/Zm2
C6(G27)
AtMYB39
AtMYB9
AtMYB107
C7(G10)
AtMYB53
AtMYB92
AtMYB93
ZmMYB086
ZmMYB013
ZmMYB062
C8(G24)

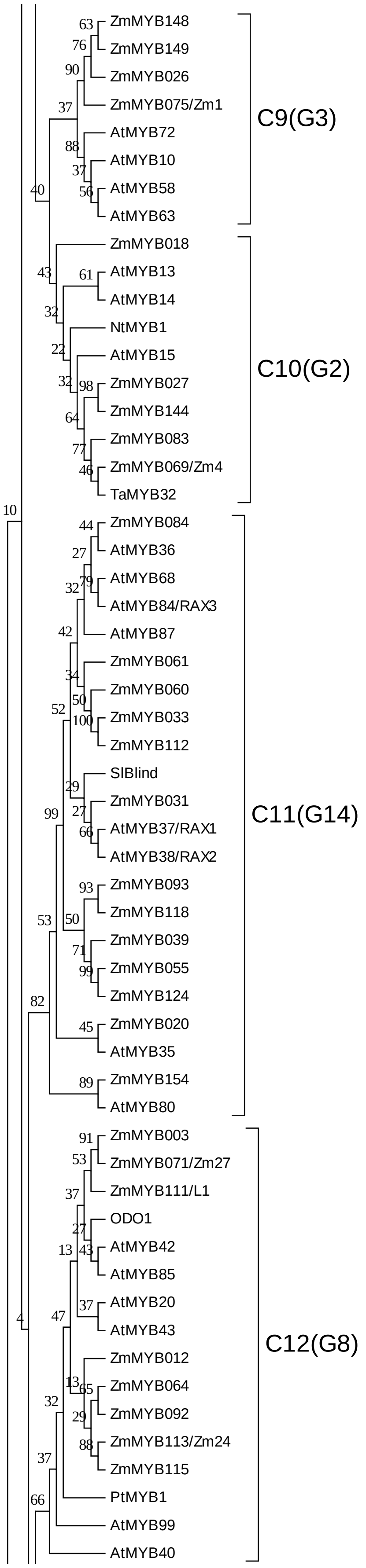
ZmMYB148
ZmMYB149
ZmMYB026
ZmMYB075/Zm1
AtMYB72
AtMYB10
AtMYB58
AtMYB63
C9(G3)
ZmMYB018
AtMYB13
AtMYB14
NtMYB1
AtMYB15
ZmMYB027
ZmMYB144
ZmMYB083
ZmMYB069/Zm4
TaMYB32
C10(G2)
ZmMYB084
AtMYB36
AtMYB68
AtMYB84/RAX3
AtMYB87
ZmMYB061
ZmMYB060
ZmMYB033
ZmMYB112
SlBlind
ZmMYB031
AtMYB37/RAX1
AtMYB38/RAX2
ZmMYB093
ZmMYB118
ZmMYB039
ZmMYB055
ZmMYB124
ZmMYB020
AtMYB35
ZmMYB154
AtMYB80
C11(G14)
ZmMYB003
ZmMYB071/Zm27
ZmMYB111/L1
ODO1
AtMYB42
AtMYB85
AtMYB20
AtMYB43
ZmMYB012
ZmMYB064
ZmMYB092
ZmMYB113/Zm24
ZmMYB115
PtMYB1
AtMYB99
AtMYB40
C12(G8)

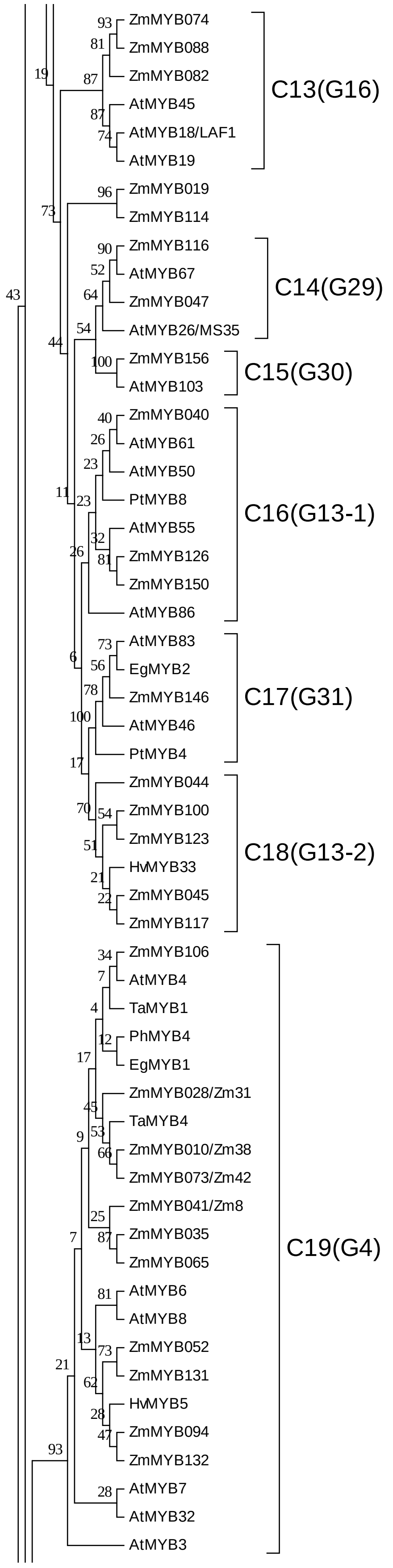

ZmMYB074
ZmMYB088
ZmMYB082
AtMYB45
AtMYB18/LAF1
AtMYB19
C13(G16)
ZmMYB019
ZmMYB114
ZmMYB116
AtMYB67
ZmMYB047
AtMYB26/MS35
C14(G29)
ZmMYB156
AtMYB103
C15(G30)
ZmMYB040
AtMYB61
AtMYB50
PtMYB8
AtMYB55
ZmMYB126
ZmMYB150
AtMYB86
C16(G13-1)
AtMYB83
EgMYB2
ZmMYB146
AtMYB46
PtMYB4
C17(G31)
ZmMYB044
ZmMYB100
ZmMYB123
HvMYB33
ZmMYB045
ZmMYB117
C18(G13-2)
ZmMYB106
AtMYB4
TaMYB1
PhMYB4
EgMYB1
ZmMYB028/Zm31
TaMYB4
ZmMYB010/Zm38
ZmMYB073/Zm42
ZmMYB041/Zm8
ZmMYB035
ZmMYB065
AtMYB6
AtMYB8
ZmMYB052
ZmMYB131
HvMYB5
ZmMYB094
ZmMYB132
AtMYB7
AtMYB32
AtMYB3
C19(G4)

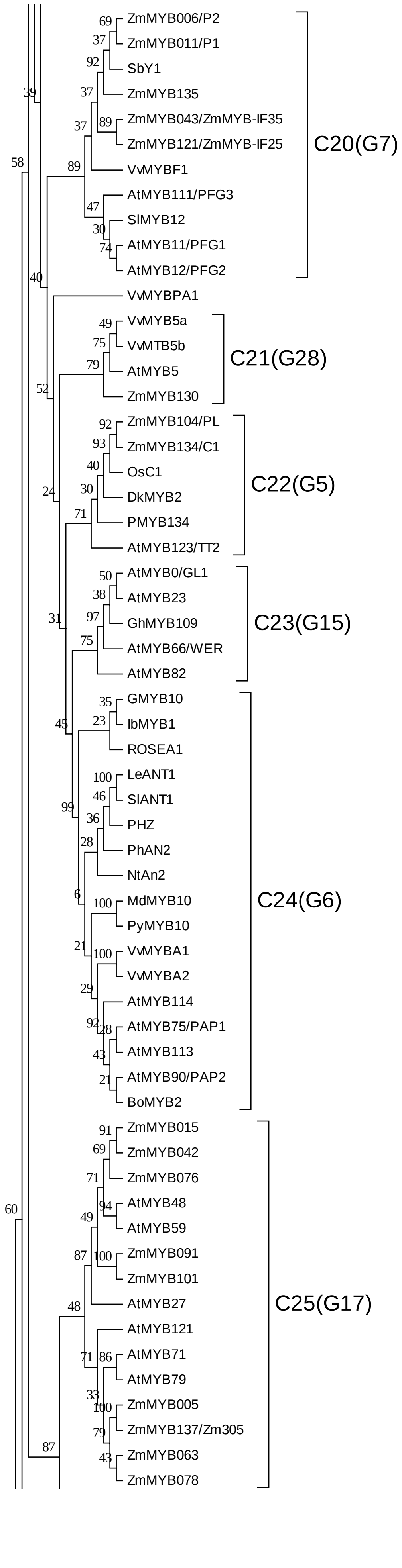

ZmMYB006/P2
ZmMYB011/P1
SbY1
ZmMYB135
ZmMYB043/ZmMYB-IF35
ZmMYB121/ZmMYB-IF25
VvMYBF1
AtMYB111/PFG3
SlMYB12
AtMYB11/PFG1
AtMYB12/PFG2
C20(G7)
VvMYBPA1
VvMYB5a
VvMTB5b
AtMYB5
ZmMYB130
C21(G28)
ZmMYB104/PL
ZmMYB134/C1
OsC1
DkMYB2
PMYB134
AtMYB123/TT2
C22(G5)
AtMYB0/GL1
AtMYB23
GhMYB109
AtMYB66/WER
AtMYB82
C23(G15)
GMYB10
IbMYB1
ROSEA1
LeANT1
SlANT1
PHZ
PhAN2
NtAn2
MdMYB10
PyMYB10
VvMYBA1
VvMYBA2
AtMYB114
AtMYB75/PAP1
AtMYB113
AtMYB90/PAP2
BoMYB2
C24(G6)
ZmMYB015
ZmMYB042
ZmMYB076
AtMYB48
AtMYB59
ZmMYB091
ZmMYB101
AtMYB27
AtMYB121
AtMYB71
AtMYB79
ZmMYB005
ZmMYB137/Zm305
ZmMYB063
ZmMYB078
C25(G17)

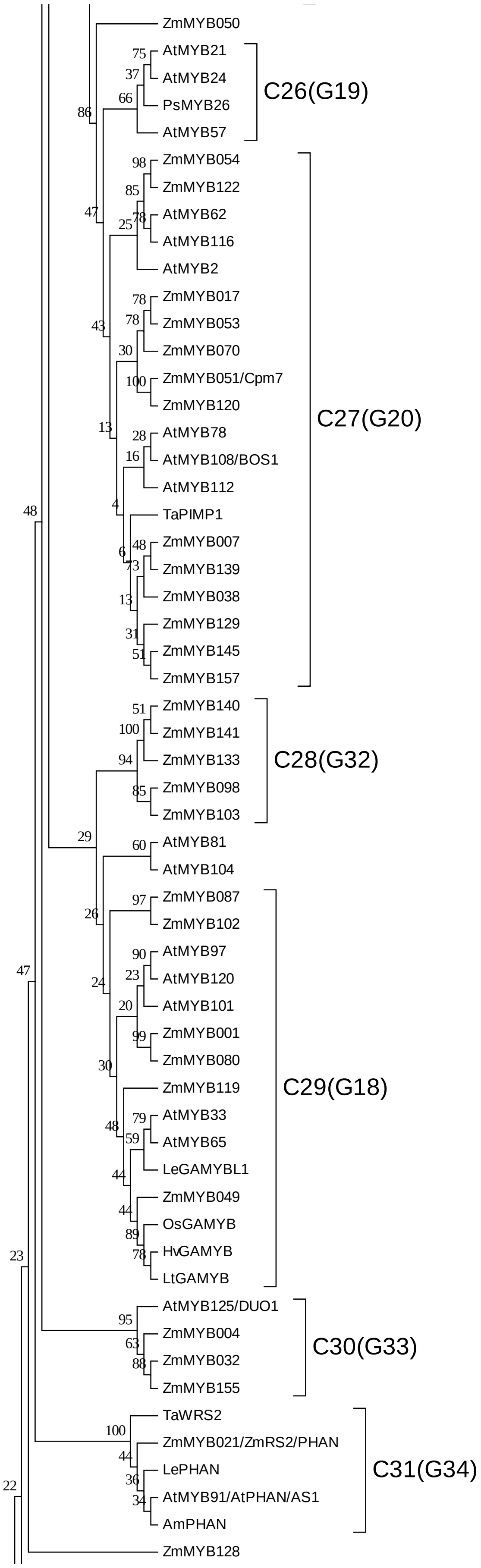

ZmMYB050
AtMYB21
AtMYB24
PsMYB26
AtMYB57
C26(G19)
ZmMYB054
ZmMYB122
AtMYB62
AtMYB116
AtMYB2
ZmMYB017
ZmMYB053
ZmMYB070
ZmMYB051/Cpm7
ZmMYB120
AtMYB78
AtMYB108/BOS1
AtMYB112
TaPIMP1
ZmMYB007
ZmMYB139
ZmMYB038
ZmMYB129
ZmMYB145
ZmMYB157
C27(G20)
ZmMYB140
ZmMYB141
ZmMYB133
ZmMYB098
ZmMYB103
C28(G32)
AtMYB81
AtMYB104
ZmMYB087
ZmMYB102
AtMYB97
AtMYB120
AtMYB101
ZmMYB001
ZmMYB080
ZmMYB119
AtMYB33
AtMYB65
LeGAMYBL1
ZmMYB049
OsGAMYB
HvGAMYB
LtGAMYB
C29(G18)
AtMYB125/DUO1
ZmMYB004
ZmMYB032
ZmMYB155
C30(G33)
TaWRS2
ZmMYB021/ZmRS2/PHAN
LePHAN
AtMYB91/AtPHAN/AS1
AmPHAN
C31(G34)
ZmMYB128

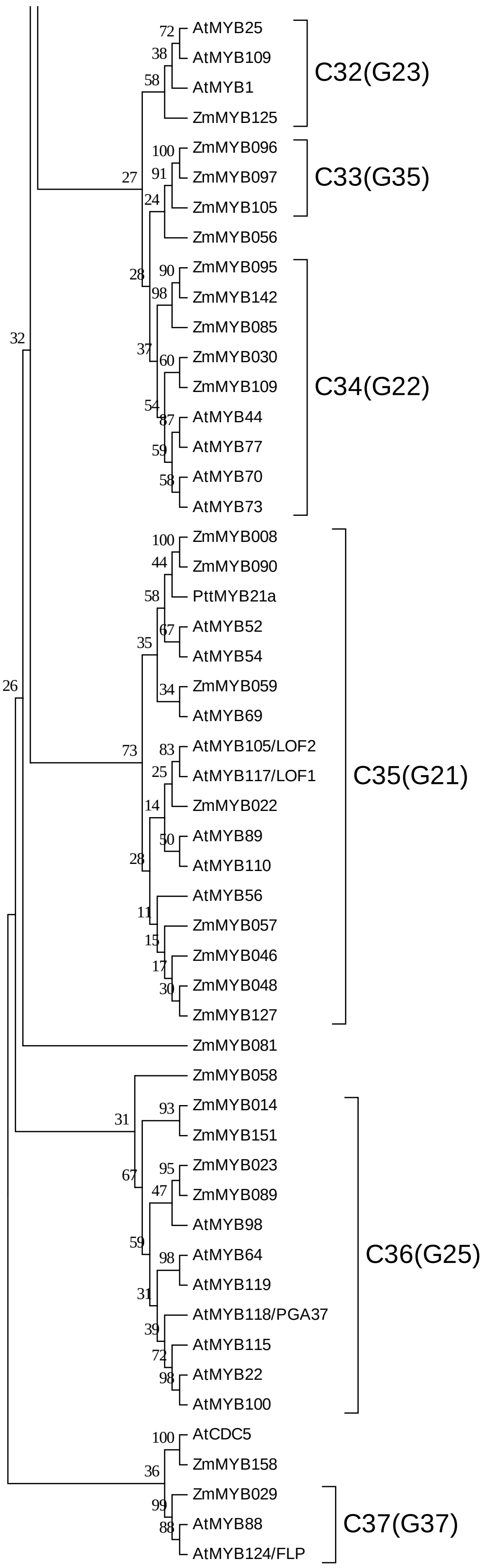

Figure S5. Maximum likelihood (ML) tree of the R2R3-MYB proteins from maize (Zm), *Arabidopsis* (At) and other plants. The unrooted tree was constructed by the Maximum likelihood (ML) method based on the alignment of the corresponding MYB domains. Boot strapping values

are indicated as percentages along the branches in ML analysis. The MYB proteins are clustered into 37 subgroups (designated as C1 to C37) and the corresponding subfamilies in NJ tree (Fig.5) were listed in round bracket, for reference.
