# Supplementary material for: The R2R3-MYB Transcription Factor Gene Family in Maize
Source: PLoS One. 2012 Jun 7;7(6):e37463. doi: 10.1371/journal.pone.0037463 (PMC3370817; doi:10.1371/journal.pone.0037463)
Supplement: Table S1 — Summary of the R2R3-MYB transcription factor genes in maize. (PDF) [file pone.0037463.s006.pdf]

Table S1. Summary of the R2R3-MYB transcription factor genes in maize

The MYB transcription factor encoding genes identified from maize in this study are listed according to the system of JGI. *, The MYB protein had undergone alternative splicing events.

Name	JGI Number	Chr
ZmMYB001	AC217264.3_FGT005	1
ZmMYB002	GRMZM2G024468_T01	1
ZmMYB003	GRMZM2G037650_T01	1
ZmMYB004	GRMZM2G046443_T01	1
ZmMYB005	GRMZM2G054111_T01	1
ZmMYB006*	GRMZM2G057027_T02	1
ZmMYB007	GRMZM2G070849_T01	1
ZmMYB008	GRMZM2G077147_T01	1
ZmMYB009	GRMZM2G079123_T01	1
ZmMYB010*	GRMZM2G084583_T01	1
ZmMYB011	GRMZM2G084799_T01	1
ZmMYB012*	GRMZM2G106558_T02	1
ZmMYB013	GRMZM2G110135_T01	1
ZmMYB014	GRMZM2G121570_T01	1
ZmMYB015*	GRMZM2G130149_T01	1
ZmMYB016	GRMZM2G131937_T01	1
ZmMYB017*	GRMZM2G143046_T01	1
ZmMYB018	GRMZM2G147346_T01	1
ZmMYB019	GRMZM2G147698_T01	1
ZmMYB020	GRMZM2G308034_T01	1
ZmMYB021	GRMZM2G403620_T01	1
ZmMYB022	GRMZM2G428555_T01	1
ZmMYB023	GRMZM5G870592_T01	1
ZmMYB024	AC165178.2_FGT004	2
ZmMYB025	GRMZM2G032655_T01	2
ZmMYB026	GRMZM2G038722_T01	2
ZmMYB027	GRMZM2G048295_T01	2
ZmMYB028	GRMZM2G050305_T01	2
ZmMYB029	GRMZM2G064630_T01	2
ZmMYB030	GRMZM2G087955_T01	2
ZmMYB031	GRMZM2G090837_T01	2
ZmMYB032	GRMZM2G105137_T01	2
ZmMYB033	GRMZM2G115859_T01	2
ZmMYB034	GRMZM2G123202_T01	2
ZmMYB035*	GRMZM2G124715_T01	2
ZmMYB036	GRMZM2G139284_T01	2
ZmMYB037	GRMZM2G166337_T01	2
ZmMYB038	GRMZM2G176327_T01	2
ZmMYB039	GRMZM2G001875_T01	3

Name	JGI Number	Chr
ZmMYB040	GRMZM2G017520_T01	3
ZmMYB041	GRMZM2G041415_T01	3
ZmMYB042	GRMZM2G047626_T01	3
ZmMYB043	GRMZM2G051256_T01	3
ZmMYB044	GRMZM2G052377_T01	3
ZmMYB045	GRMZM2G064744_T01	3
ZmMYB046	GRMZM2G083239_T01	3
ZmMYB047	GRMZM2G088783_T01	3
ZmMYB048	GRMZM2G111731_T01	3
ZmMYB049	GRMZM2G139688_T01	3
ZmMYB050	GRMZM2G143328_T01	3
ZmMYB051	GRMZM2G158700_T01	3
ZmMYB052	GRMZM2G160838_T01	3
ZmMYB053	GRMZM2G160840_T01	3
ZmMYB054*	GRMZM2G162709_T01	3
ZmMYB055	GRMZM2G167829_T01	3
ZmMYB056	GRMZM2G369799_T01	3
ZmMYB057	GRMZM2G460869_T01	3
ZmMYB058	GRMZM2G470307_T01	3
ZmMYB059	GRMZM5G803355_T01	3
ZmMYB060	GRMZM2G011422_T01	4
ZmMYB061	GRMZM2G015021_T01	4
ZmMYB062*	GRMZM2G017268_T01	4
ZmMYB063	GRMZM2G043792_T01	4
ZmMYB064	GRMZM2G055158_T01	4
ZmMYB065	GRMZM2G089244_T01	4
ZmMYB066	GRMZM2G108959_T01	4
ZmMYB067*	GRMZM2G111045_T01	4
ZmMYB068	GRMZM2G111117_T01	4
ZmMYB069	GRMZM2G127857_T01	4
ZmMYB070*	GRMZM2G131442_T01	4
ZmMYB071	GRMZM2G138427_T01	4
ZmMYB072	GRMZM2G162434_T01	4
ZmMYB073	GRMZM2G419239_T01	4
ZmMYB074	GRMZM2G496770_T01	4
ZmMYB075	GRMZM5G833253_T01	4
ZmMYB076*	GRMZM2G048136_T01	4
ZmMYB077*	GRMZM2G001223_T03	5
ZmMYB078	GRMZM2G027697_T01	5
ZmMYB079	GRMZM2G040924_T01	5
ZmMYB080*	GRMZM2G070523_T01	5
ZmMYB081	GRMZM2G073836_T01	5
ZmMYB082	GRMZM2G088189_T01	5

Name	JGI Number	Chr
ZmMYB083	GRMZM2G095904_T01	5
ZmMYB084*	GRMZM2G104789_T01	5
ZmMYB085	GRMZM2G145444_T01	5
ZmMYB086	GRMZM2G159547_T01	5
ZmMYB087	GRMZM2G161512_T01	5
ZmMYB088	GRMZM2G170049_T01	5
ZmMYB089	GRMZM2G302549_T01	5
ZmMYB090	GRMZM2G455869_T01	5
ZmMYB091	GRMZM2G013581_T01	6
ZmMYB092	GRMZM2G048910_T01	6
ZmMYB093*	GRMZM2G069325_T02	6
ZmMYB094*	GRMZM2G077789_T01	6
ZmMYB095	GRMZM2G078820_T01	6
ZmMYB096	GRMZM2G093647_T01	6
ZmMYB097	GRMZM2G093660_T01	6
ZmMYB098*	GRMZM2G093789_T01	6
ZmMYB099	GRMZM2G102790_T01	6
ZmMYB100	GRMZM2G175232_T01	6
ZmMYB101	GRMZM2G305856_T01	6
ZmMYB102	GRMZM2G343068_T01	6
ZmMYB103	GRMZM2G423833_T01	6
ZmMYB104	GRMZM2G701063_T01	6
ZmMYB105	AC213884.3_FGT002	6
ZmMYB106*	GRMZM2G000818_T01	7
ZmMYB107	GRMZM2G031323_T01	7
ZmMYB108*	GRMZM2G045748_T01	7
ZmMYB109	GRMZM2G050550_T01	7
ZmMYB110	GRMZM2G056407_T01	7
ZmMYB111*	GRMZM2G104551_T01	7
ZmMYB112	GRMZM2G117244_T01	7
ZmMYB113*	GRMZM2G126566_T01	7
ZmMYB114	GRMZM2G150841_T01	7
ZmMYB115	GRMZM2G169356_T01	7
ZmMYB116	GRMZM2G172327_T01	7
ZmMYB117	GRMZM2G003406_T01	8
ZmMYB118	GRMZM2G006352_T01	8
ZmMYB119*	GRMZM2G028054_T01	8
ZmMYB120	GRMZM2G047600_T01	8
ZmMYB121	GRMZM2G051528_T01	8
ZmMYB122	GRMZM2G096358_T01	8
ZmMYB123	GRMZM2G119693_T01	8
ZmMYB124	GRMZM2G151205_T01	8
ZmMYB125	GRMZM2G169316_T01	8

Name	JGI Number	Chr
ZmMYB126	GRMZM2G171781_T01	8
ZmMYB127	GRMZM2G312419_T01	8
ZmMYB128	GRMZM2G322490_T01	8
ZmMYB129	GRMZM2G330475_T01	8
ZmMYB130	GRMZM2G395672_T01	8
ZmMYB131	GRMZM2G405094_T01	8
ZmMYB132*	GRMZM2G431156_T01	8
ZmMYB133	GRMZM2G004090_T01	9
ZmMYB134	GRMZM2G005066_T01	9
ZmMYB135	GRMZM2G022686_T01	9
ZmMYB136	GRMZM2G044824_T01	9
ZmMYB137	GRMZM2G089686_T01	9
ZmMYB138	GRMZM2G098179_T01	9
ZmMYB139	GRMZM2G134279_T01	9
ZmMYB140	GRMZM2G167088_T01	9
ZmMYB141	GRMZM2G416652_T01	9
ZmMYB142	GRMZM5G803308_T01	9
ZmMYB143	AC197146.3_FGT002	10
ZmMYB144	AC206901.3_FGT005	10
ZmMYB145	GRMZM2G001824_T01	10
ZmMYB146	GRMZM2G052606_T01	10
ZmMYB147	GRMZM2G081557_T01	10
ZmMYB148	GRMZM2G097636_T01	10
ZmMYB149	GRMZM2G097638_T01	10
ZmMYB150	GRMZM2G127490_T01	10
ZmMYB151	GRMZM2G150680_T01	10
ZmMYB152	GRMZM2G172487_T01	10
ZmMYB153	GRMZM2G172575_T01	10
ZmMYB154	GRMZM2G173633_T01	10
ZmMYB155	GRMZM2G311059_T01	10
ZmMYB156	GRMZM2G325907_T01	10
ZmMYB157	GRMZM2G425427_T01	10
