# Supplementary material for: The R2R3-MYB Transcription Factor Gene Family in Maize
Source: PLoS One. 2012 Jun 7;7(6):e37463. doi: 10.1371/journal.pone.0037463 (PMC3370817; doi:10.1371/journal.pone.0037463)
Supplement: Table S2 — Motifs outside the MYB DNA-binding domain of maize R2R3-MYB proteins. (PDF) [file pone.0037463.s007.pdf]

Table S2: Motifs outside the MYB DBD domain of maize R2R3-MYB proteins

Motif	E-value	Consensus sequence
E1	6.70E-271	[LR][QK][MR]GIDPVTH[KR]P[LR]A[DA]AAAAA[AP]
		[HN][GE][KC][KS][RK][IE][RK][EV][IV][ED][IV][HQ][DE]L[MV]D[VE][KL]I[LV]F[QE]HLDY[HG]K
		D[LIT]V[VF]P[TI][KR]PLK[RC][RH]AS[IT][GS]S[LV][KQ]I[SP]G[EAD][TI][KER][EK]T[YF]C[SP]TD
E2	6.40E-182	LNYV[LM][AT][RK][SN][EQ]SV[PH][TV]GSAI[AG]SGYPV
E3	8.00E-177	[KR]QL[NR]CD[VA]NS[AKQ][RQ]F[KR]DA[MV]RYLWMPRL[LV]ER[IA]
E4	1.80E-82	[RP]P[AS][AV][PS]KGQWERRLQTDI[HN][TL]AR[QR]ALR[ED]AL[ST][LP][DEL]
		P[VA][YL][DG]K[NH]R[AR]ET[PT][NS]LA[RH][PL][KR][VL]PPKELFL[ED]QL[TV][AS]GHE[SN][PL
		][ST][TG]C[RH][ST][SA]G[QP][TV][LP][YN]FPFQQ[PL][LM][GC]Y[NS][SN]E[SF]G[SN][GKS][DN]G
		A[NS][MN]NSLWFN[QH][SN][DE][FT][NS][CG]STISTVMPP[VA]SPS[AT]LSTS[MT]GLN[LR][PS]PD
E5	2.50E-74	N[PS][RH][HS]GGTGI[GQ]ST
E6	5.60E-66	DWLLQCVSASVEQQIHSSIQSTETISSQTTGLL[HP]TMSEISFETLIPMVYPMIHSFGY
E7	4.40E-57	A[AKR][AG][AC][AP][SAH][LA][RS]H[MT]AQWE[ST]ARLEAEARL[AS][RL][EL][AS][RK][TL][RA]
		D[VP]SQ[YC]Y[QP]VASF[FS]D[PL]G[VI][PS]DT[ELP]L[GT][AKS][SG][FL][FL]ND[TI][PAT][PL][EA
E8	1.80E-55	][TS][SL][FL][LN][NV][QN][QL]K[QD]
		C[IV]TS[IT]L[KE][GN]T[HPQ][PS][DV]MDGYPMDQIW[MK]EIEAP[EHQ][AMV]P[SN][EL][ELV][SG
E9	2.90E-39	][FI][VA][GE]G[NK][EH][NK][VT]CSS[LS][AP][TC][PH]LP[PS]P[TV][VM]W[ED]Y
		TTT[TA]N[FC]SSGDSGEQQSN[NT][MS][SK][KE][EA][AD]DDELDLEN[FL]EMMPMLD[VD]D[DP][P
E10	1.20E-38	I][ST]FGF[GD][TM]LVD[TP][AV]PAPYG[SP]AV
		FYWD[GT][TA]NPSS[SC]SS[TR]GS[RS]GSN[SG][ML]GFE[PL]QSTS[ST][IL]LE[NS]SVF[PQ]WT[DE]
E11	2.00E-38	[IL][GT][QP][ED]K[DN][TS][RQ][AV]HL[VG][EA]ELKWPD
		[FQ][SDN][PA][EW][LF][VL][ALP][AL][MLV][QR][ELQ][MIL][IV][RS][DIT]EV[QR][RN][YKQ]M[S
E12	5.30E-44	AQ][GES][AMSV]G[LQ][RDV]
E13	1.50E-35	[LF]S[AM][IL]E[SN]WL[LF]DD
E14	9.70E-35	MQN[QL]SQ[TS]LY[DE]DVIKAESQF[NS][IM]EG[IL]CA[SA]W[FS]QN[QL]QPQQ[QH]LQ
E15	6.10E-42	[RK][CH][PL]D[LI][ND]L[DN]L[SC][IV][SG][PL][PA][CA]
E16	5.60E-41	Y[APV][SCL][ST][AM][DE]N[IV][SA][RK][LM]L[EDK][GV][WFK][ALM][GR][PC]
E17	1.10E-31	[RK][AQC][GS][LS]P[IL]YP[EA][HY][VM]L[SN]Q[VS][KS][QHN][PEK][DQ][QM][DQ]
E18	5.20E-25	TIFGSIQTEIPSIQSSSYSPSNDWLAQCPSACIEQRIPMECNTISQSKAYLMMH
E19	6.50E-28	GVDD[LT]LV[LS]PE[IF]D[IM]G[QH]E[LY]W[SE][IE][IQ][DP]G
E20	1.70E-26	[KN][ED]WWLE[DN]LE[RK]ELGLWG[TPY][VA][ED][DEV][YDE][QDT][YQ][PAH][MHY]
		VSISNLLGQSFASK[GS]CSFMDQVDQAGMLKQSG[CS][AV]LP[AT]LSDAID[DS]V[IL]SS[AV]D[HQ]
		FSNDSEKL[MR]Q[AT]LGF[DG]YLNEANA[ST]SKSIAPFGVALTGSHA[FP]LNG[IN]FSASR[LP]TNGP
E21	9.10E-23	[LS]KME
E22	1.10E-16	[DAK][KN][KA]S[VL][PE]F[FY]DFL[GQ]V[GN]A
E23	1.20E-16	[HQ]HQQQQ
E24	1.80E-16	[TI]AIAIDMSKL[QH]SA[DE]RRRGGRTPGR[PS]PK
E25	1.40E-15	[NKS][GCS][HS][HNRS][FC]LGLR[TG][AGS][MV]LDFR[GRS]L[EK]MK
E26	1.60E-15	K[RK][KR][KLP]KKK
E27	6.50E-19	[ED][DE]DK[DE][YF]WLK[MV][LF][DM]E[SVA][SNG]
E28	2.40E-15	[AQ]DQEFGSG[ED]xE[DS]G[LW]WSL[ED][DE][IL]W
		PWDG[TA]TDWLLDYQDFG[ML][CG][SD]S[NS][LS]VD[DGN][SY][MV][FV][HNR][AN][SN][NS][G
E29	5.60E-15	S][LNS][NG][FA]
