# Supplementary material for: The R2R3-MYB Transcription Factor Gene Family in Maize
Source: PLoS One. 2012 Jun 7;7(6):e37463. doi: 10.1371/journal.pone.0037463 (PMC3370817; doi:10.1371/journal.pone.0037463)
Supplement: Table S3 — Summary of functionally characterized R2R3-MYBs from plants examined in this study by MYB subgroups. (PDF) [file pone.0037463.s008.pdf]

Table S3. Summary of functionally characterized R2R3-MYB genes from plants examined in this study by MYB subgroups

Subgroup	Reported Members	Biological Functions	References
G1	AtMYB30, AtMYB60, AtMYB96, TaMYB1	Abiotic stress response/HR response, SA-mediated (VLC-Lipid metabolism); Development/Hypocotyle elongation, brassinosteroid pathway; Biotic stress response/Drought, ABA-mediated (stomatal closure), and pathogens ABA-and JA-mediated	1-5
G2	AtMYB13, AtMYB15, NtMYB1, TaMYB32	Abiotic stress response/Drought, light, wounding, salt, and Cold, ABA-mediated, disease resistance,	6-9
G3	AtMYB58, AtMYB63, AtMYB72, Zm1	Phenylpropanoide pathway/Lignin biosynthesis (fibers and vessels), Biotic stress response/Pathogens (induced systemic resistance)	10-12
G4	AtMYB3, AtMYB4, AtMYB7, AtMYB32, EgMYB1, TaMYB4, PhMYB4, HvMYB5, ZmMYB31, ZmMYB38, ZmMYB42, ZmMYB8	Phenylpropanoid pathway/sinapate ester biosynthesis, lignin biosynthesis, floral volatile benzenoid/phenylpropanoid (FVBP) biosynthesis, secondary cell wall formation	13-20
G5	AtMYB123, PL, C1, OSC1 PMYB134, DkMYB2	Proanthocyanidin biosynthesis, anthocyanin biosynthesis,	21-26
G6	AtMYB75, AtMYB90, AtMYB113, AtMYB114, IbMYB1, MdMYB10, PyMYB10, GhMYB10, ROSEA1, PhAn2, LeAN1, NtAn2, VvMYBA1, VvMYBA2, PHZ, BoMYB2	Anthocyanin biosynthesis	27-38
G7	AtMYB11, AtMYB12, AtMYB111, SlMYB12, SbY1, VvMYBF1, P1, P2, ZmMYB-IF25, ZmMYB-IF35, VvMYBPA1	Flavonol biosynthesis, anthocyanin biosynthesis, proanthocyanidin synthesis	39-49
G8	AtMYB85, PtMYB1, ODO1	Metabolism/Lignin deposition, cell wall thickening, fragrance biosynthesis	50-52
G9	AtMYB16, AtMYB17, AtMYB106, AmMYBL1, AmMYBL2, AmMYBL3, AmMIXTA	Cell fate/Conical epidermal cell outgrowth, richome branching	53-58
G11	AtMYB41, AtMYB102, DcMYB1	Abiotic stress response/Osmotic, ABA-mediated, elicitor treatment, UV-B irradiation and the dilution effect	59-61
G12	AtMYB28, AtMYB29, AtMYB34, AtMYB51, AtMYB76, AtMYB122	Glucosinolate biosynthesis/Aliphatic pool and Indolic pool	62-65
G13	AtMYB61, HvMYB33, PtMYB8	Phenylpropanoide pathway/lignin biosynthesis, Mucilage deposition and extrusion, stomatal closure	66-68
G14	AtMYB37, AtMYB38, AtMYB68, AtMYB84, AtMYB80, SlBlind	Development/Axillary meristem regulation, lateral organ formation (shoot branching, GA-mediated), hypocotyl elongation, blue light-mediated, root elongation	69-73
G15	AtMYB0, AtMYB23, AtMYB66, GhMYB109	Cell fate/ Trichome initiation and branching, and Root hair patterning, elongating fibers	74-79
G16	AtMYB18	Hypocotyl elongation, far red light-mediated (phytochrome signalling)	80
G18	AtMYB33, AtMYB65, AtMYB101, OsGAMYB, HvGAMYB, LtGAMYB	Stamen development/Anther development (tapetum); Abiotic stress response/ABA-, gibberellin-mediated; impair alpha-amylase expression in aleurone and flower development	81-92
G19	AtMYB21, AtMYB24, AtMYB57, PsMYB26	Stamen development/Filament lenght, GA- and JA-mediated, dehiscence process, phenylpropanoid metabolism	93-95
G20	AtMYB2, AtMYB62, AtMYB108, TaPIMP1	Abiotic stress response/Drought, salt, wounding, pathogens, ABA-, JA-, and GA-mediated, phosphate starvation; Stamen development/pollen maturation; dehiscence process	96-99
G21	AtMYB52, AtMYB54, AtMYB69, AtMYB105, AtMYB117, PttMYB21a	Metabolism/Cell wall thickening (fibers); axillary meristem regulation/lateral organ formation; vascular tissue formation and lignification	100-102
G22	AtMYB44, AtMYB70, AtMYB73,AtMYB77	Abiotic stress response/Drought, salt, cold, light, wounding; ABA-mediated (stomatal closure); growth regulation, auxin-mediated	103-105
G25	AtMYB115, AtMYB118	Embryogenesis/seed maturation	106
G28	AtMYB5, VvMYB5a, VvMYB5b, VvMYBPA1	Phenylpropanoide pathway/Proanthocyanindins biosynthesis; Mucilage biosynthesis;	107-112
G29	AtMYB26	Stamen development /Anther development (endothecium)	113
G30	AtMYB103	Cell wall thickening (fibers)	50, 112
G31	AtMYB46, PtMYB4, EdMYB2	Cell wall thickening (fibers and vessels), lignification	114-116
G33	AtMYB125	Stamen development, pollen formation	117
G34	AtMYB91, ZmRS2, TaWRS2, AmPHAN, LePHAN	Axillary meristem regulation/Lateral organ separation (Leaves)	118-122
G37	AtMYB88, AtMYB124	Cell fate/Stomata cell differentiation	123

Supplementary references

[1]. **Li L, Yu X, Thompson A, Guo M, Yoshida S, et al.** (2009) Arabidopsis MYB30 is a direct target of BES1 and cooperates with BES1 to regulate brassinosteroid-induced gene expression. Plant J 58: 275–286

[2]. **Cominelli E, Galbiati M, Vavasseur A, Conti L, Sala T, et al.** (2005) A guard-cell-specific MYB transcription factor regulates stomatal movements and plant drought tolerance. Curr Biol 15:1196–1200

[3]. **Seo PJ, Xiang F, Qiao M, Park JY, Lee YN, et al.** (2009) The MYB96 transcription factor mediates abscisic acid signaling during drought stress response in Arabidopsis. Plant Physiol 151:275–289

[4]. **Oh JE, Kwon Y, Kim JH, Noh H, Hong SW, et al.** (2011) A dual role for MYB60 in stomatal regulation and root growth of Arabidopsis thaliana under drought stress. Plant Mol Biol 77:91–103

[5]. **Lee1 TG, Jang CS, Kim JY, Kim DS, Park JH, et al.** (2007) A Myb transcription factor (TaMyb1) from wheat roots is expressed during hypoxia: roles in response to the oxygen concentration in root environment and abiotic stresses. Physiologia Plantarum 129:375–385

[6]. **Kirik V, Kölle K, Wohlfarth T, Miséra S, Bäumlein H** (1998) Ectopic expression of a novel MYB gene modifies the architecture of the Arabidopsis inflorescence. Plant J 3:729-742

[7]. **Ding Z, Li S, An X, Liu X, Qin H, et al.** (2009) Transgenic expression of MYB15 confers enhanced sensitivity to abscisic acid and improved drought tolerance in Arabidopsis thaliana. J Genet Genomics 36:17–29

[8]. **Yang Y, Klessig DF** (1996) Isolation and characterization of a tobacco mosaic virus-inducible myb oncogene homolog from tobacco. Proc Natl Acad Sci U S A, 93:14972-14977

[9]. **Zhang L, Zhao G, Jia J, Liu X, Kong X** (2012) Molecular characterization of 60 isolated wheat MYB genes and analysis of their expression during abiotic stress. J Exp Bot 63:203–214.

[10]. **Segarra G, Van der Ent S, Trillas I, Pieterse CM** (2009) MYB72, a node of convergence in induced systemic resistance triggered by a fungal and a bacterial beneficial microbe. Plant Biol (Stuttg) 11:90-96

[11]. **Zhou J, Lee C, Zhong R, Ye ZH** (2009) MYB58 and MYB63 are transcriptional activators of the lignin biosynthetic pathway during secondary cell wall formation in Arabidopsis. Plant Cell 21:248-266

[12]. **Franken P, Schrell S, Peterson PA, Saedler H, Wienand U** (1994) Molecular analysis of protein domain function encoded by the myb-homologous maize genes C1, Zm 1 and Zm 38. Plant J 6:21-30

[13]. **Jin H, Cominelli E, Bailey P, Parr A, Mehrtens F, et al.** (2000) Transcriptional repression by AtMYB4 controls production of UV–protecting sunscreens in Arabidopsis. EMBO J 19:6150–6161

[14]. **Preston J, Wheeler J, Heazlewood J, Li SF, Parish RW** (2004) AtMYB32 is required for normal pollen development in Arabidopsis thaliana. Plant J 40:979–995

[15]. **Legay S, Sivadon P, Blervacq AS, Pavy N, Baghdady A, et al.** (2010) EgMYB1, an R2R3 MYB transcription factor from eucalyptus negatively regulates secondary cell wall formation in Arabidopsis and poplar. New Phytol 188:774–786

[16]. **Ma QH, Wang C, Zhu HH** (2011) TaMYB4 cloned from wheat regulates lignin biosynthesis through negatively controlling the transcripts of both cinnamyl alcohol dehydrogenase and cinnamoyl-CoA reductase genes. Biochimie 93:1179–1186

[17]. **Colquhoun TA, Kim JY, Wedde AE, Levin LA, Schmitt KC, et al.** (2011) PhMYB4 fine-tunes the floral volatile signature of Petunia x hybrida through PhC4H. J Exp Bot 62:1133–1143

[18]. **Sonbol FM, Fornale S, Capellades M, Encina A, Tourin S, et al.** (2009) The maize ZmMYB42 represses the phenylpropanoid pathway and affects the cell wall structure, composition and degradability in Arabidopsis thaliana. Plant Mol Biol 70:283–296

[19]. **Fornalé S, Sonbol FM, Maes T, Capellades M, Puigdomènech P, et al.** (2006) Down-regulation of the maize and Arabidopsis thaliana caffeic acid O-methyl-transferase genes by two new maize R2R3-MYB transcription factors. Plant Mol Biol 62:809–823

[20]. **Wissenbach M, Uberlacker B, Vogt F, Becker D, Salamini F, Rohde W** (1993) Myb genes from Hordeum vulgare: tissue-specific expression of chimeric Myb promoter/Gus genes in transgenic tobacco. Plant J 4:411-422

[21]. **Nesi N, Jond C, Debeaujon I, Caboche M, Lepiniec L** (2001) The Arabidopsis TT2 gene encodes an R2R3 MYB domain protein that acts as a key determinant for proanthocyanidin accumulation in developing seed. Plant Cell 13:2099–2114

[22]. **Pilu R, Piazza P, Petroni K, Ronchi A, Martin C, et al.** (2003) pl-bol3, a complex allele of the anthocyanin regulatory pl1 locus that arose in a naturally occurring maize population. Plant J 36:510–521

[23]. **Paz–Ares J, Ghosal D, Wienand U, Peterson PA, Saedler H** (1987) The regulatory c1 locus of maize encodes a protein with homology to myb proto–oncogene products and with structural similarities to transcriptional activators. EMBO J 6:3553–3558

[24]. **Mellway RD, Tran LT, Prouse MB, Campbell MM, Constabel CP** (2009) The wound-, pathogen-, and ultraviolet B-responsive MYB134 gene encodes an R2R3 MYB transcription factor that regulates proanthocyanidin synthesis in poplar. Plant Physiol 150:924–941

[25]. **Akagi T, Ikegami A, Yonemori K** (2010) DkMyb2 wound-induced transcription factor of persimmon (Diospyros kaki Thunb.), contributes to proanthocyanidin regulation. Planta 232:1045–1059

[26]. **Saitoh K, Onishi K, Mikami I, Thidar K, Sano Y** (2004) Allelic diversification at the C (OsC1) locus of wild and cultivated rice:

nucleotide changes associated with phenotypes. Genetics 168:997-1007

[27]. Gonzalez A, Zhao M, Leavitt JM, Lloyd AM (2008) Regulation of the anthocyanin biosynthetic pathway by the TTG1/bHLH/Myb transcriptional complex in Arabidopsis seedlings. Plant J 53:814–827

[28]. Quattrocchio F, Wing J, van der Woude K, Souer E, de Vetten N, et al. (1999) Molecular analysis of the anthocyanin2 gene of petunia and its role in the evolution of flower color. Plant Cell 11:1433–1444

[29]. Pattanaik S, Kong Q, Zaitlin D, Werkman J R, Xie C H, et al. (2010) Isolation and functional characterization of a floral tissue–specific R2R3 MYB regulator from tobacco. Planta 231:1061–1076

[30]. Mathews H, Clendennen SK, Caldwell CG, Liu XL, Connors K, et al. (2003) Activation tagging in tomato identifies a transcriptional regulator of anthocyanin biosynthesis, modification, and transport. Plant Cell 15:1689–1703

[31]. Espley RV, Hellens RP, Putterill J, Stevenson DE, Kutty-Amma S, et al. (2006) Red colouration in apple fruit is due to the activity of the MYB transcription factor, MdMYB10. Plant J 49:414–427

[32]. Fournier-Level A, Lacombe T, Le Cunff L, Boursiquot JM, This P (2010) Evolution of the VvMybA gene family, the major determinant of berry colour in cultivated grapevine (Vitis vinifera L.). Heredity 104:351–362

[33]. Feng S, Wang Y, Yang S, Xu Y, Chen X (2010) Anthocyanin biosynthesis in pears is regulated by a R2R3-MYB transcription factor PyMYB10. Planta 232:245–255

[34]. Schwinn K, Venail J, Shang Y, Mackay S, Alm V, et al. (2006) A small family of MYB-regulatory genes controls floral pigmentation intensity and patterning in the genus Antirrhinum. Plant Cell 18:831–851

[35]. Elomaa P, Uimari A, Mehto M, Albert VA, Laitinen RA, et al. (2003) Activation of anthocyanin biosynthesis in Gerbera hybrida (Asteraceae) suggests conserved protein-protein and protein-promoter interactions between the anciently diverged monocots and eudicots. Plant Physiol 133:1831–1842

[36]. Mano H, Ogasawara F, Sato K, Higo H, Minobe Y (2007) Isolation of a regulatory gene of anthocyanin biosynthesis in tuberous roots of purple-fleshed sweet potato. Plant Physiol 143:1252–1268

[37]. Chiu LW, Zhou X, Burke S, Wu X, Prior RL, et al. (2010) The purple cauliflower arises from activation of a MYB transcription factor. Plant Physiol 154:1470–1480.

[38]. Albert NW, Lewis DH, Zhang H, Schwinn KE, Jameson PE, et al. (2011) Members of an R2R3-MYB transcription factor family in Petunia are developmentally and environmentally regulated to control complex floral and vegetative pigmentation patternin. Plant J 65:771–784

[39]. Grotewold E, Athma P, Peterson T (1991) Alternatively spliced products of the maize P gene encode proteins with homology to the DNA-binding domain of Myb-like transcription factors. Proc Natl Acad Sci USA 88: 4587–4591

[40]. Cone KC, Cocciolone SM, Burr FA, Burr B (1993) Maize anthocyanin regulatory gene pl is a duplicate of c1 that functions in the plant. Plant Cell 5:1795–1805

[41]. Heine GF, Malik V, Dias AP, Grotewold E (2007) Expression and molecular characterization of ZmMYB-IF35 and related R2R3-MYB transcription factors. Mol Biotechnol 37:155–164

[42]. Dias AP, Braun EL, McMullen MD, Grotewold E (2003) Recently duplicated maize R2R3 Myb genes provide evidence for distinct mechanisms of evolutionary divergence after duplication. Plant Physiol 131:610–620

[43]. Mehrtens F, Kranz H, Bednarek P, Weisshaar B (2005) The Arabidopsis transcription factor MYB12 is a flavonol–specific regulator of phenylpropanoid biosynthesis. Plant Physiol 138: 1083–1096

[44]. Stracke R, Ishihara H, Huep G, Barsch A, Mehrtens F, et al. (2007) Differential regulation of closely related R2R3–MYB transcription factors controls flavonol accumulation in different parts of the Arabidopsis thaliana seedling. Plant J 50: 660–677

[45]. Czemmel S, Stracke R, Weisshaar B, Cordon N, Harris NN, et al. (2009) The grapevine R2R3-MYB transcription factor VvMYBF1 regulates flavonol synthesis in developing grape berries. Plant Physiol 151:1513–1530

[46]. Ballester AR, Molthoff J, de Vos R, Hekkert BL, Orzaez D, et al. (2010) Biochemical and molecular analysis of pink tomatoes: deregulated expression of the gene encoding transcription factor SlMYB12 leads to pink tomato fruit color. Plant Physiol 152:71–84

[47]. Boddu J, Jiang C, Sangar V, Olson T, Peterson T, et al. (2006) Comparative structural and functional characterization of sorghum and maize duplications containing orthologous myb transcription regulators of 3-deoxyflavonoid biosynthesis. Plant Mol Biol 60:185–199

[48]. Zhang F, Peterson T (2005) Comparisons of maize pericarp color1 alleles reveal paralogous gene recombination and an organ-specific enhancer region. Plant Cell 17:903–914

[49]. Bogs J, Jaffé FW, Takos AM, Walker AR, Robinson SP (2007) The grapevine transcription factor VvMYBPA1 regulates proanthocyanidin synthesis during fruit development. Plant Physiol 143:1347–1361.

[50]. Zhong R, Lee C, Zhou J, McCarthy RL, Ye ZH (2008) A battery of transcription factors involved in the regulation of secondary cell wall biosynthesis in Arabidopsis. Plant Cell 20:2763-2782.

[51]. Patzlaff A, Newman LJ, Dubos C, Whetten RW, Smith C, et al. (2003) Characterisation of PtMYB1, an R2R3-MYB from pine xylem. Plant Mol Biol 53:597-608

[52]. **Verdonk JC, Haring MA, van Tunen AJ, Schuurink RC** (2005) ODORANT1 regulates fragrance biosynthesis in petunia flowers. Plant Cell 17:1612-1624

[53]. **Baumann K, Perez–Rodriguez M, Bradley D, Venail J, Bailey P, et al.** (2007) Control of cell and petal morphogenesis by R2R3 MYB transcription factors. Development 134:1691–1701

[54]. **Zhang Y, Cao G, Qu LJ, Gu H** (2007) Characterization of Arabidopsis MYB transcription factor gene AtMYB17 and its possible regulation by LEAFY and AGL15. J Genet Genomics 36:99-107.

[55]. **Jakoby MJ, Falkenhan D, Mader MT, Brininstool G, Wischnitzki E, et al.** (2008) Transcriptional profiling of mature Arabidopsis trichomes reveals that NOECK encodes the MIXTA-like transcriptional regulator MYB106. Plant Physiol 148:1583–1602

[56]. **Glover BJ, Perez-Rodriguez M, Martin C** (1998) Development of several epidermal cell types can be specified by the same MYB-related plant transcription factor. Development 125:3497–3508

[57]. **Perez-Rodriguez M, Jaffe FW, Butelli E, Glover BJ, Martin C** (2005) Development of three different cell types is associated with the activity of a specific MYB transcription factor in the ventral petal of Antirrhinum majus flowers. Development 132:359–370

[58]. **Jaffé FW, Tattersall A, Glover BJ** (2007) A truncated MYB transcription factor from Antirrhinum majus regulates epidermal cell outgrowth. J Exp Bot 58:1515–1524

[59]. **Lippold F, Sanchez DH, Musialak M, Schlereth A, Scheible WR, et al. (2009)** AtMyb41 regulates transcriptional and metabolic responses to osmotic stress in Arabidopsis. Plant Physiol 149:1761–1772

[60]. **De Vos M, Denekamp M, Dicke M, Vuylsteke M, Van Loon L, et al.** (2006) The Arabidopsis thaliana Transcription Factor AtMYB102 Functions in Defense Against the Insect Herbivore Pieris rapae. Plant Signal Behav 1:305–311

[61]. **Maeda K, Kimura S, Demura T, Takeda J, Ozeki Y** (2005) DcMYB1 acts as a transcriptional activator of the carrot phenylalanine ammonia-lyase gene (DcPAL1) in response to elicitor treatment, UV-B irradiation and the dilution effect. Plant Mol Biol, 59:739–752

[62]. **Gigolashvili T, Yatusevich R, Berger B, Müller C, Flügge UI** (2007) The R2R3–MYB transcription factor HAG1/MYB28 is a regulator of methionine–derived glucosinolate biosynthesis in Arabidopsis thaliana. Plant J 51:247–261

[63]. **Gigolashvili T, Engqvist M, Yatusevich R, Müller C, Flügge UI** (2008) HAG2/MYB76 and HAG3/MYB29 exert a specific and coordinated control on the regulation of aliphatic glucosinolate biosynthesis in Arabidopsis thaliana. New Phytol 177:627–42

[64]. **Celenza JL, Quiel JA, Smolen GA, Merrikh H, Silvestro AR, et al.** (2005) The Arabidopsis ATR1 Myb transcription factor controls indolic glucosinolate homeostasis. Plant Physiol 137:253–262

[65]. **Gigolashvili T, Berger B, Mock HP, Müller C, Weisshaar B, et al.** (2007) The transcription factor HIG1/MYB51 regulates indolic glucosinolate biosynthesis in Arabidopsis thaliana. Plant J 50:886–901

[66]. **Bomal C, Bedon F, Caron S, Mansfield SD, Levasseur C, et al.** (2008) Involvement of Pinus taeda MYB1 and MYB8 in phenylpropanoid metabolism and secondary cell wall biogenesis: a comparative in planta analysis. J Exp Bot, 59:3925–3939

[67]. **Liang YK, Dubos C, Dodd IC, Holroyd GH, Hetherington AM, et al.** (2005) AtMYB61, an R2R3-MYB transcription factor controlling stomatal aperture in Arabidopsis thaliana. Curr Biol, 15:1201–1206

[68]. **Wissenbach M, Uberlacker B, Vogt F, Becker D, Salamini F, et al.** (1993) Myb genes from Hordeum vulgare: tissue-specific expression of chimeric Myb promoter/Gus genes in transgenic tobacco. Plant J, 4:411–422

[69]. **Schmitz G, Tillmann E, Carriero F, Fiore C, Cellini F, et al.** (2002) The tomato Blind gene encodes a MYB transcription factor that controls the formation of lateral meristems. Proc Natl Acad Sci USA 99:1064–1069

[70]. **Keller T, Abbott J, Moritz T, Doerner P** (2006) Arabidopsis REGULATOR OF AXILLARY MERISTEMS1 controls a leaf axil stem cell niche and modulates vegetative development. Plant Cell 18:598–611

[71]. **Müller D, Schmitz G, Theres K** (2006) Blind homologous R2R3 Myb genes control the pattern of lateral meristem initiation in Arabidopsis. Plant Cell 18:586–597

[72]. **Feng Caiping, Andreasson E, Maslak A, Mock HP, Mattsson O, et al.** (2004) Arabidopsis MYB68 in development and responses to environmental cues. Plant Science 167:1099–1107.

[73]. **Zhang ZB, Zhu J, Gao JF, Wang C, Li H, et al.** (2007) Transcription factor AtMYB103 is required for anther development by regulating tapetum development, callose dissolution and exine formation in Arabidopsis. Plant J 52:528–538.

[74]. **Kirik V, Lee MM, Wester K, Herrmann U, Zheng Z, et al.** (2005) Functional diversification of MYB23 and GL1 genes in trichome morphogenesis and initiation. Development 132:1477–1485

[75]. **Kang YH, Kirik V, Hulskamp M, Nam KH, Hagely K, et al.** (2009) The MYB23 gene provides a positive feedback loop for cell fate specification in the Arabidopsis root epidermis. Plant Cell 21:1080–1094

[76]. **Suo J, Liang X, Pu L, Zhang Y, Xue Y** (2003) Identification of GhMYB109 encoding a R2R3 MYB transcription factor that expressed specifically in fiber initials and elongating fibers of cotton (Gossypium hirsutum L.). Biochim Biophys Acta 1630:25-34

[77]. **Lee MM, Schiefelbein J** (1999) WEREWOLF, a MYB-related protein in Arabidopsis, is a position-dependent regulator of epidermal cell patterning. Cell 99:473–483

[78]. Tominaga R, Iwata M, Okada K, Wada T (2007) Functional analysis of the epidermal–specific MYB genes CAPRICE and WEREWOLF in Arabidopsis. Plant Cell 19: 2264–2277

[79]. Pu L, Li Q, Fan X, Yang W, Xue Y (2008) The R2R3 MYB transcription factor GhMYB109 is required for cotton fiber development. Genetics 180:811–820

[80]. Yang SW, Jang IC, Henriques R, Chua NH (2009) FAR-RED ELONGATED HYPOCOTYL1 and FHY1-LIKE associate with the Arabidopsis transcription factors LAF1 and HFR1 to transmit phytochrome A signals for inhibition of hypocotyl elongation. Plant Cell, 21:1341–1359

[81]. Woodger FJ, Gubler F, Pogson BJ, Jacobsen JV (2003) A Mak-like kinase is a repressor of GAMYB in barley aleurone. Plant J 33:707–717

[82]. Gubler F, Kalla R, Roberts JK, Jacobsen JV (1995) Gibberellin-regulated expression of a myb gene in barley aleurone cells: evidence for Myb transactivation of a high-pI alpha-amylase gene promoter. Plant Cell 7:1879–1891

[83]. Gocal GF, Poole AT, Gubler F, Watts RJ, Blundell C, et al. (1999) Long-day up-regulation of a GAMYB gene during Lolium temulentum inflorescence formation. Plant Physiol 119:1271–1278

[84]. Tsuji H, Aya K, Ueguchi-Tanaka M, Shimada Y, Nakazono M, et al. (2006) GAMYB controls different sets of genes and is differentially regulated by microRNA in aleurone cells and anthers. Plant J 47:427–444

[85]. Aya K, Ueguchi-Tanaka M, Kondo M, Hamada K, Yano K, et al. (2009) Gibberellin modulates anther development in rice via the transcriptional regulation of GAMYB. Plant Cell 21:1453–1472

[86]. Gocal GF, Sheldon CC, Gubler F, Moritz T, Bagnall DJ, et al (2001) GAMYB–like genes, flowering, and gibberellin signaling in Arabidopsis. Plant Physiol 127:1682–1693

[87]. Murray F, Kalla R, Jacobsen J, and Gubler F (2003) A role for HvGAMYB in anther development. Plant J 33: 481–491

[88]. Millar AA, and Gubler F (2005) The Arabidopsis GAMYB-like genes, MYB33 and MYB65, are microRNA-regulated genes that redundantly facilitate anther development. Plant Cell 17:705–721

[89]. Achard P, Herr A, Baulcombe DC, Harberd NP (2004) Modulation of floral development by a gibberellin-regulated micro-RNA. Development 131:3357–3365

[90]. Kaneko M, Inukai Y, Ueguchi-Tanaka M, Itoh H, Izawa T, et al. (2004) Loss-of-function mutations of the rice GAMYB gene impair alpha-amylase expression in aleurone and flower development. Plant Cell 16:33–44

[91]. Rhoades MW, Reinhart BJ, Lim LP, Burge CB, Bartel B, et al. (2002). Prediction of plant microRNA targets. Cell 110:513–520

[92]. Reyes JL, Chua NH (2007) ABA induction of miR159 controls transcript levels of two MYB factors during Arabidopsis seed germination. Plant J 49:592–606

[93]. Mandaokar A, Thines B, Shin B, Lange BM, Choi G, et al. (2006) Transcriptional regulators of stamen development in Arabidopsis identified by transcriptional profiling. Plant J, 46:984–1008

[94]. Cheng H, Song S, Xiao L, Soo HM, Cheng Z, et al. (2009) Gibberellin acts through jasmonate to control the expression of MYB21, MYB24, and MYB57 to promote stamen filament growth in Arabidopsis. PLoS Genet, 5:e1000440

[95]. Uimari A, Strommer J (1997) Myb26: a MYB-like protein of pea flowers with affinity for promoters of phenylpropanoid genes. Plant J 12:1273–1284

[96]. Mandaokar A, Browse J (2009) MYB108 acts together with MYB24 to regulate jasmonate-mediated stamen maturation in Arabidopsis. Plant Physiol, 149:851–862

[97]. Abe H, Urao T, Ito T, Seki M, Shinozaki K, et al. (2003) Arabidopsis AtMYC2 (bHLH) and AtMYB2 (MYB) function as transcriptional activators in abscisic acid signaling. Plant Cell, 15:63–78

[98]. Devaiah BN, Madhuvanthi R, Karthikeyan AS, Raghothama KG. (2008) Phosphate starvation responses and gibberellic acid biosynthesis are regulated by the MYB62 transcription factor in Arabidopsis. Mol Plant, 2:43–58

[99]. Liu H, Zhou X, Dong N, Liu X, Zhang H, et al. (2011) Expression of a wheat MYB gene in transgenic tobacco enhances resistance to Ralstonia solanacearum, and to drought and salt stresses. Funct Integr Genomics, 11:431–443.

[100]. Zhong R, Lee C, Zhou J, McCarthy RL, Ye ZH (2008) A battery of transcription factors involved in the regulation of secondary cell wall biosynthesis in Arabidopsis. Plant Cell 20:2763–2782

[101]. Karpinska B, Karlsson M, Srivastava M, Stenberg A, Schrader J, et al. (2004) MYB transcription factors are differentially expressed and regulated during secondary vascular tissue development in hybrid aspen. Plant Mol Biol 56:255–270

[102]. Lee DK, Geisler M, Springer PS (2009) LATERAL ORGAN FUSION1 and LATERAL ORGAN FUSION2 function in lateral organ separation and axillary meristem formation in Arabidopsis. Development 136:2423–2432

[103]. Jung C, Seo JS, Han SW, Koo YJ, Kim CH, et al. (2008) Overexpression of AtMYB44 enhances stomatal closure to confer abiotic stress tolerance in transgenic Arabidopsis. Plant Physiol 146: 623–635

[104]. Shin R, Burch AY, Huppert KA, Tiwari SB, Murphy AS, et al. (2007) The Arabidopsis transcription factor MYB77 modulates auxin signal transduction. Plant Cell 19:2440–2453

[105]. Shinozaki K, Yamaguchi-Shinozaki K, Urao T, Koizumi M (1992) Nucleotide sequence of a gene from Arabidopsis thaliana

encoding a myb homologue. Plant Mol Biol 19:493–499.

[106]. Wang X, Niu QW, Teng C, Li C, Mu J, et al. (2009) Overexpression of PGA37/MYB118 and MYB115 promotes vegetative-to-embryonic transition in Arabidopsis. Cell Res, 19:224–235

[107]. Li SF, Milliken ON, Pham H, Seyit R, Napoli R, et al. (2009) The Arabidopsis MYB5 transcription factor regulates mucilage synthesis, seed coat development, and trichome morphogenesis. Plant Cell 21:72–89

[108]. Deluc L, Barrieu F, Marchive C, Lauvergeat V, Decendit A, et al. (2006) Characterization of a grapevine R2R3-MYB transcription factor that regulates the phenylpropanoid pathway. Plant Physiol 140:499–511

[109]. Bogs J, Jaffé FW, Takos AM, Walker AR, Robinson SP (2007) The grapevine transcription factor VvMYBPA1 regulates proanthocyanidin synthesis during fruit development. Plant Physiol 143:1347–1361

[110]. Deluc L, Bogs J, Walker AR, Ferrier T, Decendit A, et al. (2008) The transcription factor VvMYB5b contributes to the regulation of anthocyanin and proanthocyanidin biosynthesis in developing grape berries. Plant Physiol 147:2041–2053

[111]. Martin C, Paz–Ares J (1997) MYB transcription factors in plants. Trends Genet 13:67–73

[112]. Lai LB, Nadeau JA, Lucas J, Lee EK, Nakagawa T, et al. (2005) The Arabidopsis R2R3 MYB proteins FOUR LIPS and MYB88 restrict divisions late in the stomatal cell lineage. Plant Cell 17:2754–2767

[113]. Yang C, Xu Z, Song J, Conner K, Vizcay Barrena G, et al. (2007) Arabidopsis MYB26/MALE STERILE35 regulates secondary thickening in the endothecium and is essential for anther dehiscence. Plant Cell 19:534-548

[114]. Zhong R, Richardson EA, Ye ZH (2007) The MYB46 transcription factor is a direct target of SND1 and regulates secondary wall biosynthesis in Arabidopsis. Plant Cell 19:2776–2792

[115]. Patzlaff A, McInnis S, Courtenay A, Surman C, Newman LJ, et al. (2003) Characterisation of a pine MYB that regulates lignification. Plant J 36:743–754.

[116]. Goicoechea M, Lacombe E, Legay S, Mihaljevic S, Rech P, et al. (2005) EgMYB2, a new transcriptional activator from Eucalyptus xylem, regulates secondary cell wall formation and lignin biosynthesis. Plant J 43:553–567

[117]. Rotman N, Durbarry A, Wardle A, Yang WC, Chaboud A, et al. (2005) A novel class of MYB factors controls sperm-cell formation in plants. Curr Biol, 15:244–248

[118]. Waites R, Selvadurai HR, Oliver IR, Hudson A (1998) The PHANTASTICA gene encodes a MYB transcription factor involved in growth and dorsoventrality of lateral organs in Antirrhinum. Cell 93:779–789

[119]. Koltai H, Bird DM (2000) Epistatic repression of PHANTASTICA and class 1 KNOTTED genes is uncoupled in tomato. Plant J 22:455–459

[120]. Morimoto R, Nishioka E, Murai K, Takumi S (2009) Functional conservation of wheat orthologs of maize rough sheath1 and rough sheath2 genes. Plant Mol Biol 69:273–285

[121]. Tsiantis M, Schneeberger R, Golz JF, Freeling M, Langdale JA (1999) The maize rough sheath2 gene and leaf development programs in monocot and dicot plants. Science 284:154–156

[122]. Byrne ME, Barley R, Curtis M, Arroyo JM, Dunham M, et al. (2000) Asymmetric leaves1 mediates leaf patterning and stem cell function in Arabidopsis. Nature 408:967–971

[123]. Xie Z, Lee E, Lucas JR, Morohashi K, Li D, et al. (2010) Regulation of cell proliferation in the stomatal lineage by the Arabidopsis MYB FOUR LIPS via direct targeting of core cell cycle genes. Plant Cell, 22:2306–2321
