# Supplementary material for: The R2R3-MYB Transcription Factor Gene Family in Maize
Source: PLoS One. 2012 Jun 7;7(6):e37463. doi: 10.1371/journal.pone.0037463 (PMC3370817; doi:10.1371/journal.pone.0037463)
Supplement: Table S4 — Details of primers used in the RT-PCR analysis. (PDF) [file pone.0037463.s009.pdf]

Table S4. Details of primers used in the RT-PCR analysis

Name	sequence (5' to 3')
ZmMYB001-1F	CGAGCCTCGAACAGATGCTG
ZmMYB001-1R	TGGGACTGAAACTCCGCAATC
ZmMYB002-1F	GCGATGTACGCTTCCAGCATC
ZmMYB002-1R	CGGAGAACGCCGAGAAGGA
ZmMYB003-1F	GACCCTGAACTGCTGGTGAATTG
ZmMYB003-1R	CCAGTCTGTTGTTCCGTCCCAC
ZmMYB004-1F	CACGCACTGCCGCTACGAC
ZmMYB004-1R	GCAGCAGAAAGAGAGCAGACGA
ZmMYB005-1F	GCCGACAGTACCTCCAGAGCC
ZmMYB005-1R	GCTGTCCAACATCGCCATCAC
ZmMYB006-1F	GGACGCCCTGATGCCTATTG
ZmMYB006-1R	CGAAGCCATCCCAGTCCATG
ZmMYB007-1F	GTCCTCTCCGTCCTCGGATTC
ZmMYB007-1R	GACCAGGCACGATTGTTGCTG
ZmMYB008-1F	CCCGTTTGCCATTAACCATGATAG
ZmMYB008-1R	CGAACCACACTTGGTGTCGCTC
ZmMYB009-1F	CAGAGGAGTGATGACTTGGAGGATG
ZmMYB009-1R	GAGAACCACGGCTCCTCACAAC
ZmMYB010-1F	GTTCCAGCCCAGCCCCAG
ZmMYB010-1R	TGATGCAGAGGTCGAGGTTGAG
ZmMYB011-1F	TGGGTGCTGGAGCCGATAGAG
ZmMYB011-1R	ATAGGCATCAGGGCGTCCATC
ZmMYB012-1F	CTGTTCCCGCTCATGGACTTG
ZmMYB012-1R	GTAGACAGGGGATGGCGACAG
ZmMYB013-1F	CTCTGCTCGCCGCAACTGAC
ZmMYB013-1R	GAACGTGGTGCGCGGTAAGTAG
ZmMYB014-1F	ATGATGGAGCACGATCACTACCAG
ZmMYB014-1R	GCTGCCACATGACGCTGTTG
ZmMYB015-1F	CGTCCTCGTCTTCGCTGACTTAC
ZmMYB015-1R	GTGCCCTTCAGGATGCTTGTG
ZmMYB016-1F	GGCGTAATATCTGCGAGCAACAG
ZmMYB016-1R	CCGTCAGCCTTGGTCATCCTG
ZmMYB017-1F	CCTCTGGATGCCGCACCTC
ZmMYB017-1R	GAAAAGCAGGCCTCCGTCATC
ZmMYB018-1F	CACCAGGGACGAGGACATGGA
ZmMYB018-1R	TTACAAGACGCTCAAGTCCCTCATG
ZmMYB019-1F	GGTAACGTCAAGGCCATGCTACC
ZmMYB019-1R	AGTGTTCCGCTTCCGCCATC
ZmMYB020-1F	TGGCCGTTGTTGGGCAGTAC
ZmMYB020-1R	CCAGGATGGCGTCGATGAAC
ZmMYB021-1F	GGAGAAGATGGCCGAGCAGTG
ZmMYB021-1R	GTTCATGTCGGTGGCAGAGGAC

Name	sequence (5' to 3')
ZmMYB022-1F	CCAATACTGCACCTTCCCGTTC
ZmMYB022-1R	TCACCAGGGAACAAGCAGAAGG
ZmMYB023-1F	TGCTCGCAGCTTCGATATCCTC
ZmMYB023-1R	CTGTCCATCATGCAAAGTAATACGTG
ZmMYB024-1F	TGCAGACCGACATCCACACG
ZmMYB024-1R	CGGCAGAGGCTCCACCTTAG
ZmMYB025-1F	AGAGCTACTGGGACAGCATAATGAAC
ZmMYB025-1R	GGGCACAGGCACAGCGACT
ZmMYB026-1F	GGGAAAGGAGTGGTGGTTGGAG
ZmMYB026-1R	CCCGCTTGGAAGTAGCAGGAC
ZmMYB027-1F	GGTCCGAGACGCTGTCGATG
ZmMYB027-1R	ACACTCTGAGCCAGTAGTCCAGGTC
ZmMYB028-1F	GAGGAGGCTGCGATGAGAGTG
ZmMYB028-1R	AGGCTTCTGAAGTCGAGCATGG
ZmMYB029-1F	AGTCAGGGGAGCAGAATGGACAG
ZmMYB029-1R	CGGTTATCTGCTGAACAAGTTTCCAT
ZmMYB030-1F	TCCAGCAACCTCCTTGTCGTTG
ZmMYB030-1R	GGATCATCTCCTGCATCATCAACAG
ZmMYB031-1F	GGCTTGGCACGGGAAACTTC
ZmMYB031-1R	CTGCTGCCTCCTCGCCATAG
ZmMYB032-1F	ACATACACGCCTGCTCGTCATC
ZmMYB032-1R	AGGAGCCATGTTCCAGCCAG
ZmMYB033-1F	CAGAGACACCATGACGGCAGTAG
ZmMYB033-1R	CTCCCCGAAGCACTGAATCTG
ZmMYB034-1F	ACATGGCCCAGTGGGAGACC
ZmMYB034-1R	AGGAGGAAGAGGCGGTGGC
ZmMYB035-1F	ATCGACCTGAACCTGTCCATCAG
ZmMYB035-1R	GGCTGTTGAGGCAGAGGCAC
ZmMYB036-1F	CGGTGTTCGAGTACGAGACGAAG
ZmMYB036-1R	TCTGGACAGGACGGTGGAAGTG
ZmMYB037-1F	CGGTGACGAGCAGCGGTAAC
ZmMYB037-1R	CGACGCAGGTGGTGGAGTGA
ZmMYB038-1F	TGTCGTCGTCGTCGTCCGT
ZmMYB038-1R	AGTGGGAGGCGTCGTTGTTG
ZmMYB039-1F	CCTCTTCCTGCTGGACTTCTGC
ZmMYB039-1R	TCGGGTCGAGAGGGTAGTAGTAGC
ZmMYB040-1F	CACGGAACATTCACGGACACAC
ZmMYB040-1R	GCCCTCTATGTTGAATTGGCTCTC
ZmMYB041-1F	AAGCAGCTTTCGCCACCATG
ZmMYB041-1R	GTGGGCTTAGCGAGAGGTTGAG
ZmMYB042-1F	CTCGGAGGTGGGCTTTGTTG
ZmMYB042-1R	ACCAAATTGTGGTGCCATCTTG
ZmMYB043-1F	CTGAGCGAGGAGATGGTGAGTG

Name	sequence (5' to 3')
ZmMYB043-1R	GCCACTGTAGCTGTCCATGCAC
ZmMYB044-1F	CTGTTCATGTAGGGTACGCCACTG
ZmMYB044-1R	ACCATCGCCCGTAAGAAGCC
ZmMYB045-1F	ATGAGCAGCGACGACCCTCTC
ZmMYB045-1R	GCGGAACGAGCCGTAGGAG
ZmMYB046-1F	CACTGCTGCGCTGCATCATG
ZmMYB046-1R	AACGCCCAGGAAGTCGAAGAAG
ZmMYB047-1F	ACTACAACATTCTGGATGGAGGAGC
ZmMYB047-1R	ATGGAAGGAGAAGGCTGCGTC
ZmMYB048-1F	CAGTACACGGCAGCGGACAC
ZmMYB048-1R	GTCGTAGCCACTCTGGTAGAAGCA
ZmMYB049-1F	TCCTCCTGTTAGTGCTGCGTCAC
ZmMYB049-1R	TGCCGACTCATGTTTAGGCTCTAAC
ZmMYB050-1F	AGATGTGCTACCCGTACTGCTCC
ZmMYB050-1R	GATCGGTTTTGACTCCCAGAAATC
ZmMYB051-1F	AGCGTTGACATGTTCGACGAGA
ZmMYB051-1R	CCACCAGTTGTCTCCAGTTTCTCC
ZmMYB052-1F	CGGACAACGAGATCAAGAACTACTG
ZmMYB052-1R	GGTGGTAGCACAGGCAGATGG
ZmMYB053-1F	CGTCGTCTGGTCATGGTCTGTC
ZmMYB053-1R	GCATCTTCCAAATTTCCTCCAAG
ZmMYB054-1F	TACGGGTACGGCCCTTATTACAGTT
ZmMYB054-1R	GGGAGTTGAGGTAGTCCAGCAGC
ZmMYB055-1F	ACGGACGTGCATGGCACAA
ZmMYB055-1R	TCACCACCGCAGCAGTAGCAT
ZmMYB056-1F	CCGTCTCTGTCCCTCAGCCTT
ZmMYB056-1R	TCCTCCAGTAACGGCGATGC
ZmMYB057-1F	TAGAAGGAGCCTCTAACATGGACCC
ZmMYB057-1R	CTTTTCGGTGGCTAGTGATGGG
ZmMYB058-1F	AGGATAATACGGATGGCAAGGAACT
ZmMYB058-1R	TATCGGAAACTTCTAAGGTTGGGG
ZmMYB059-1F	GCCATCGGCTCTGTCGTTGTA
ZmMYB059-1R	TGCAGCTGGTCTGCTGAGTCC
ZmMYB060-1F	ATGTTGCGGAGTGGTGGAGC
ZmMYB060-1R	AACGCCGTGTTGGGTTCAG
ZmMYB061-1F	CCAGCGTAGAGATGAACTCGGTC
ZmMYB061-1R	GGAAGTCGTAGAGCTCGTCGTGA
ZmMYB062-1F	ACTGACTGCTGCTACGACTGCC
ZmMYB062-1R	GCCTGTTTGCTGTTGCTGGTT
ZmMYB063-1F	TCGGCGAGCGAGGAAGG
ZmMYB063-1R	GAAAGCGAGCCCTTGATCCTG
ZmMYB064-1F	TTCCTCGAAGTGGACGGCAT
ZmMYB064-1R	ATCCAGACTTGTTCATCAAAGCCAT

Name	sequence (5' to 3')
ZmMYB065-1F	CGGCGGTGTGCCTGTGC
ZmMYB065-1R	GCCTGTAAAAAATTCAGAGCCCA
ZmMYB066-1F	GAGGCGATGACTGATACTACTACCG
ZmMYB066-1R	CCATCTGTTCCTTGCAGACTATTGA
ZmMYB067-1F	CGAAGTACCAGATGTTCCTCGACTT
ZmMYB067-1R	CAGCGAGCACGTCGAGCG
ZmMYB068-1F	TACCTGTGGCACTCTCACTGGC
ZmMYB068-1R	GCTGTTCGTGTCTTCGATTCTCG
ZmMYB069-1F	CGACCAACGACGACATGGACT
ZmMYB069-1R	CTAAATAATCTGGGGCAAATTCTGC
ZmMYB070-1F	ACTATGCAGCAAAATCATCATCAGC
ZmMYB070-1R	CTCCAGATGTCGTCGAGGCTC
ZmMYB071-1F	CTAAAACCAGCAACTCCAGAGAGG
ZmMYB071-1R	TGCTGCCTGAGCCTGACTCTG
ZmMYB072-1F	GCAGCTTCAGCGGCCTACTT
ZmMYB072-1R	GTCTTCGGACAGATCCATGAGGTC
ZmMYB073-1F	CCAGCCCAGCCCCAACAC
ZmMYB073-1R	AGGAAGTGGTGCCCGTTGC
ZmMYB074-1F	CGTGTCCCTGCACGGTACGT
ZmMYB074-1R	CCGAAGTAGTACTCGCCAATGGA
ZmMYB075-1F	CGATTGGTGGCTGGAGAATTTG
ZmMYB075-1R	GTCTGGAAGTAGGTGGAGACTGGG
ZmMYB076-1F	TGAGTATTGCTGAAGGGAAAGAGAAG
ZmMYB076-1R	TCCAGAATATCTCAGGGCATTTGTAG
ZmMYB077-1F	AGGAGGAAGAGGACAAGGGCTACT
ZmMYB077-1R	GGGTCCGTGACAGCATCGG
ZmMYB078-1F	GCTCCAGCGGGGACCAC
ZmMYB078-1R	CTAGTAGAAAGCGAGCCCTTGATCC
ZmMYB079-1F	GCCTCTCCCTCCTCGCCA
ZmMYB079-1R	CGGTCTTCAAGTCCACGGTG
ZmMYB080-1F	GACGAACCAGCAGACGGAGACT
ZmMYB080-1R	CACGGGCGACGACGAGTG
ZmMYB081-1F	CGTTTATCAACAGACCCCAAACTTA
ZmMYB081-1R	TCTGAGATTTTGGCTGAATGGGT
ZmMYB082-1F	TTCGGGGACCAGATGAGCG
ZmMYB082-1R	CCACCGTAGATGTCTCCCATATTG
ZmMYB083-1F	GCTTCTGGTCCGAGACGCTG
ZmMYB083-1R	AATCTGGGGCGAATTCTGCA
ZmMYB084-1F	GAGCCCATTGGTTTGCCCTA
ZmMYB084-1R	GGCTCATCAACAACAACGCAGTA
ZmMYB085-1F	CGCTCACCTCGCTCTCCCT
ZmMYB085-1R	AGGGAAGGCGGGTGGAGTAG
ZmMYB086-1F	GAAAATTCCCTTCCGCCGC

Name	sequence (5' to 3')
ZmMYB086-1R	ACTGTTCATCAAACAACTCGGGC
ZmMYB087-1F	CTAACCCGAGTATGGTCAAGGTCTC
ZmMYB087-1R	ACCACGGTGGAGGTCCTGTC
ZmMYB088-1F	AGGGGTCCGTTCAGGTGGAT
ZmMYB088-1R	TCATGGAGAGCAGGTCGCAGA
ZmMYB089-1F	CGATGATGATGATGTGGGACGA
ZmMYB089-1R	GGTCCATCTCCTCCTTCACGC
ZmMYB090-1F	TACGATTTTCTCCAAGTGAATGCGA
ZmMYB090-1R	CCCCAACCTCCAGGAAGTCTATGA
ZmMYB091-1F	CGCAGGAGTGCTACGACTTCC
ZmMYB091-1R	AGTAATAACAGTGACCACCAACAGCA
ZmMYB092-1F	CTGCGGCTCTGTTTCCTCTG
ZmMYB092-1R	CCCCAGTCGGCGTCCAT
ZmMYB093-1F	GGGTACGGCGACTTCTTCGG
ZmMYB093-1R	CCATATCTCGGCCACGTTGG
ZmMYB094-1F	CACGCCCACCTCGCAGTC
ZmMYB094-1R	TAACGACTCTCCTCTCGGTTCTGA
ZmMYB095-1F	ACCGCCCTCAGCCTCTCG
ZmMYB095-1R	GCCATGTACCGCTGCACCT
ZmMYB096-1F	CGAATCTTGAGCAGAATCCGTG
ZmMYB096-1R	CGTGACCTGCCGCATCTCC
ZmMYB097-1F	ACAATGAGGTCGAATCTTGGGC
ZmMYB097-1R	GCGGCATCTCCTGCATCTTC
ZmMYB098-1F	TTTTCTGATCTTGGGATCTCGGATA
ZmMYB098-1R	TGCTCCTCCAAGTGATGTATCATTG
ZmMYB099-1F	CGATGCCGCCACCTTTGT
ZmMYB099-1R	CAACTCTCTCCAACTGTCGCTGTT
ZmMYB100-1F	TGTCGGAGAACCTGGGATACG
ZmMYB100-1R	GGGCAGCAAGAACCTATGATGC
ZmMYB101-1F	GGCAAGCCAGCGTCAACAC
ZmMYB101-1R	AGGAGTCGAGCATCTTCTCGTAGTAC
ZmMYB102-1F	CCGACCCGAGCATGGTCAAG
ZmMYB102-1R	TGTCCTCTCCCAGAGGGCAGTG
ZmMYB103-1F	ACAAAATGATGGTGGTGAGGGTG
ZmMYB103-1R	TCTCCTTGTCTTCATTGTCAACGTG
ZmMYB104-1F	GCCGAACAGACAATGAAATCAAGAAC
ZmMYB104-1R	TCCATCCAGTCGCCGTTGC
ZmMYB105-1F	CCATCTGCTGGATCGTCCTAAGG
ZmMYB105-1R	GACAGCGAACGATGCCGATG
ZmMYB106-1F	CATCAACGAGCACACGTCCAAC
ZmMYB106-1R	CTGATGCAGAGGTCCAGGTTCAG
ZmMYB107-1F	GGAGCAGCATGATCACAAGCAG
ZmMYB107-1R	TGGCGGTGGTGGAGTGATTC

Name	sequence (5' to 3')
ZmMYB108-1F	CGAGTGCTCGACGGAGACCAG
ZmMYB108-1R	CACCTCAGCGTGCCCCATG
ZmMYB109-1F	GATCGAGGTACGGAACTACATGTCC
ZmMYB109-1R	CCATACCCATCATACGCTTGCTC
ZmMYB110-1F	GGGCCGGTCTTCGAATACG
ZmMYB110-1R	CTGGACAGCATGGTGCAAGTG
ZmMYB111-1F	GAACGACGACGCCGTGATAAG
ZmMYB111-1R	TCTTCCAAAAGCCACTTCACCAG
ZmMYB112-1F	GCTGTCTCCCTCTTCTTCTCCTTC
ZmMYB112-1R	GCCTGGCATGTGAGTCCAGAG
ZmMYB113-1F	CGACTCAGCAGGAGCAGGACAC
ZmMYB113-1R	CGTCGATGTCGTCAAGGAGGTG
ZmMYB114-1F	GACCCGGCCACGCACAAG
ZmMYB114-1R	CCGACGACGACAGGATGAGC
ZmMYB115-1F	TCACGGCGATCACGTCAGG
ZmMYB115-1R	GCGAGTAAACAGTGCCGAAGC
ZmMYB116-1F	GCAGGGATGGAAGTGGCTCAG
ZmMYB116-1R	ACCGCCACCGTTCATCACAG
ZmMYB117-1F	CAGCAGGTGTTCGACCCGTTC
ZmMYB117-1R	GCCGTCGAGCGCATTGTTG
ZmMYB118-1F	TCTTCGCTCGCCGAGTTCTC
ZmMYB118-1R	GGCAGGAACATGTCGTCGAGTAG
ZmMYB119-1F	TGCTGAAACAATCTGGCAGTGC
ZmMYB119-1F	TGACGATGTGATTTCCTCGGTTG
ZmMYB119-1R	GCGTTGGCTTCATTGAGATAACC
ZmMYB119-1R	AGCGTTGGCTTCATTGAGATAACC
ZmMYB120-1F	GCGACGAGCGTTGACATGTTC
ZmMYB120-1R	CAGATGTCCTCCAAACTCCACCAG
ZmMYB121-1F	AAGGGCAGGAACAACGTAGCAG
ZmMYB121-1R	GGAGGCGCAGTGAGACTGAGTC
ZmMYB122-1F	TGCACCCCTCGCACAACATC
ZmMYB122-1R	ACGACCCACCTTAGTTCGGATTC
ZmMYB123-1F	GTGACCGACTTCGGCTTCGA
ZmMYB123-1R	CGACACGTCCAGCACGCTG
ZmMYB124-1F	TCCACGACACAAGGAAGGTCG
ZmMYB124-1R	TGGTGGGTATGGTGGATGTCG
ZmMYB125-1F	TCGGGCAAGGCACATCACAG
ZmMYB125-1R	CTTGTTGGGTAGGTTCCGTGTGTC
ZmMYB126-1F	TATTTCCCTTTCCAACAGCCTTTAG
ZmMYB126-1R	GAAACTGGCGGCATCACTGTAG
ZmMYB127-1F	ACGCCCACACGCAGTACACG
ZmMYB127-1R	CCACGTCGTAGCCACTCTGGTAG
ZmMYB128-1F	ATTTGGCAGAGGCATCGTTTC

Name	sequence (5' to 3')
ZmMYB128-1R	GTCCATCGCCGCTGCCT
ZmMYB129-1F	CACCAGTACTACAGCGAAACCG
ZmMYB129-1R	CGATCATGGATGAGCTGGAGTC
ZmMYB130-1F	GGACGACGCCACCTTCTCC
ZmMYB130-1R	CTCCACCCTGGTCGTTCCC
ZmMYB131-1F	ACGACCATCTGCCTGTGCTACC
ZmMYB131-1R	TATATGTACTGGCCCTCCTCGAGC
ZmMYB132-1F	ACATCAAACGCAAGCTCCTGG
ZmMYB132-1R	AACGGACAGGTCGAGGTTGAG
ZmMYB133-1F	TACTCTGAACATGAAAGCTCTCCGA
ZmMYB133-1R	GAAGAAACTTGCCCCTAGCTCTGT
ZmMYB134-1F	GCGACGGTGACTGCGACTG
ZmMYB134-1R	TCTGACAGCGGAGCCAGTCC
ZmMYB135-1F	GAGCAGCAGAACGACCACAGC
ZmMYB135-1R	ACGAGTCGGACAGGAGCCAA
ZmMYB136-1F	ACTCCCTCTCTGGGCTCCTCA
ZmMYB136-1R	ACGTTGCTCCCGGTGGTG
ZmMYB137-1F	CCATGATGGACAGCCTGCAAA
ZmMYB137-1R	CCGTCCACCAGCCTCCACA
ZmMYB138-1F	AAGCTGCAGACCGACATCAACC
ZmMYB138-1R	CACCCGTCCAGCATCATCCT
ZmMYB139-1F	CAACAATGGTCGTGCCTGCTC
ZmMYB139-1R	GTGCTCCAGATGCTCTCCACG
ZmMYB140-1F	CAACCGCAACAAGACATGAACTG
ZmMYB140-1F	TTCAAAGGTTTTGTTGGAACCACTA
ZmMYB141-1F	CTCAGATTGGAGGCACTGGTTTTC
ZmMYB141-1R	GGTGGGTCATAAAACAATGTTGAGAGT
ZmMYB142-1F	GACCGTAGCGACCTGAGCCAC
ZmMYB142-1R	CATGAACACGGGGCTGAACG
ZmMYB143-1F	CACCTCGCCCCTCCGAAC
ZmMYB143-1R	CATCAGCCGCTGGTGTGCT
ZmMYB144-1F	CGACACGTTCTGGTCGGAGAC
ZmMYB144-1R	AACACTCCGAGCCAGTAGTCCAAG
ZmMYB145-1F	AAGGCTACGAACAAACTACGACTGAC
ZmMYB145-1R	CCGTACTCGAAATCCTCAAACCC
ZmMYB146-1F	GCCAGCAGTACATGAGCAGGAAT
ZmMYB146-1R	TCAAGAAAAGGCAAGGATGACACA
ZmMYB147-1F	CCACGGCTTATCGACACCAAG
ZmMYB147-1R	ATGTCCTTCCAGAAATGATCTTCG
ZmMYB148-1F	GCGGAGGGAAAGGAGTGGTG
ZmMYB148-1R	GCCCATTGGGTACTGGTAGTCC
ZmMYB149-1F	ATCGACATCGGCCATGAGCT
ZmMYB149-1R	CCCCACAGGCCCAACTCC

Name	sequence (5' to 3')
ZmMYB150-1F	AGCACCGAGAGAACCTCAGGG
ZmMYB150-1R	CATACACGGGCATCAGCATTG
ZmMYB151-1F	TTCACTTGGCTCCCGTTTGGT
ZmMYB151-1R	CATGAAGAACTCCCTCGACGCT
ZmMYB152-1F	GCTGTCCTCAATAATGGAGAACTGG
ZmMYB152-1R	AGCCGTCGGAGTCGGAGAG
ZmMYB153-1F	CTTTCTTTTCACTCGACCAAACGTC
ZmMYB153-1R	CCAGTTCTCCATTATTGAGGACAGC
ZmMYB154-1F	CGACCCCATCACGCACAAGT
ZmMYB154-1R	GTGAGGAGGTGGAGCATCTCGT
ZmMYB155-1F	GACGGACAACGACGTGAAGAAC
ZmMYB155-1R	GCAATGTGTGCTGCAACGTG
ZmMYB156-1F	GCACTGCCCCTTCTTCATGTTC
ZmMYB156-1R	ACCAAGGGCGGCAGATGG
ZmMYB157-1F	CCACCAATACTACAGCGAGCCG
ZmMYB157-1R	GCCTTGTTGGTCGTCTGTCGT
Actin-lF	TCACTACGACTGCCGAGCGAG
Actin-lR	GAGCCACCACTGAGGACAACATTAC
